# Progestogens and androgens influence root morphology of angiosperms in a brassinosteroid‐independent manner

**DOI:** 10.1111/tpj.70459

**Published:** 2025-09-09

**Authors:** Karl Ludwig Körber, Sudip Paul, Jana Oklestkova, Emanuel Barth, Felix Feistel, Henk Oppermann, Ceren Oktay, Maja Dorfner, Miroslav Strnad, Jennifer Munkert, Alexandra C. U. Furch, Jan Klein

**Affiliations:** ^1^ Plant Physiology, Matthias Schleiden Institute for Genetics, Bioinformatics and Molecular Botany Friedrich Schiller University Jena 07743 Jena Germany; ^2^ Plant Defense Genetics, Department of Plant and Environmental Sciences University of Copenhagen 1871 Frederiksberg Copenhagen Denmark; ^3^ Laboratory of Growth Regulators, Faculty of Science Palacký University & Institute of Experimental Botany, Czech Academy of Sciences Šlechtitelů 27 CZ‐77900 Olomouc Czech Republic; ^4^ Bioinformatics Core Facility Friedrich Schiller University Jena 07743 Jena Germany; ^5^ Department for Biochemistry Max Planck Institute for Chemical Ecology 07743 Jena Germany; ^6^ Department of Biology University of Erlangen‐Nuremberg 91058 Erlangen Germany

**Keywords:** *Arabidopsis thaliana*, root development, progestogens, androgens, brassinosteroids, phytohormones, signalling molecules, DET2

## Abstract

Progestogens and androgens are steroids found in a wide range of plants, but little is known about their physiological functions. In this study, we sowed seeds of angiosperms on progestogen‐ and androgen‐containing medium and analysed their morphological effects. We further investigated the effects of progesterone and testosterone on brassinosteroid profiles and gene expression in *A. thaliana*. Additionally, we examined the effects of progesterone and testosterone on *A. thaliana* plants overexpressing the steroid 5α‐reductase DET2. We found that progestogens and androgens have strong negative effects on root length, especially in *Brassicaceae* species. In addition, these steroids led to uncoordinated cell growth and increased lateral root formation. We failed to detect an effect on endogenous brassinosteroid levels and gene expression of brassinosteroid‐regulated genes. The overexpression of DET2 led to increased root growth, but the effects of progesterone and testosterone were not reduced. We conclude that progestogens and androgens act in a brassinosteroid‐independent manner. This suggests that progestogens and androgens could represent a potential new class of plant steroid signalling molecules.

## Significance Statement

Plants produce progesterone and androgens, which alter root morphology in *A. thaliana* and 60% of tested angiosperms. Brassinosteroids seem uninvolved. Understanding the mechanism is like navigating an unknown city—exemplarily Dittelbrunn (a small village in Bavaria)—where the expected shortcut (interaction with brassinosteroids) is blocked, requiring a longer scientific route.

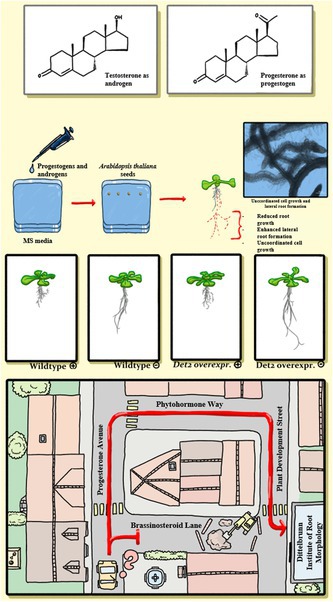

Brassinosteroids are well‐characterised phytohormones involved in plant growth, yet plants also synthesise other steroids such as progestogens and androgens, whose physiological roles remain largely unknown. Here, we demonstrate for the first time that exogenous progesterone and testosterone alter root morphology in *Arabidopsis thaliana* and various angiosperms, independently of brassinosteroid biosynthesis or signalling. These findings prompt a reconsideration of the functional relevance of non‐canonical plant steroids and lay the foundation for future investigations into their biological significance.

## INTRODUCTION

**Figure 1:**
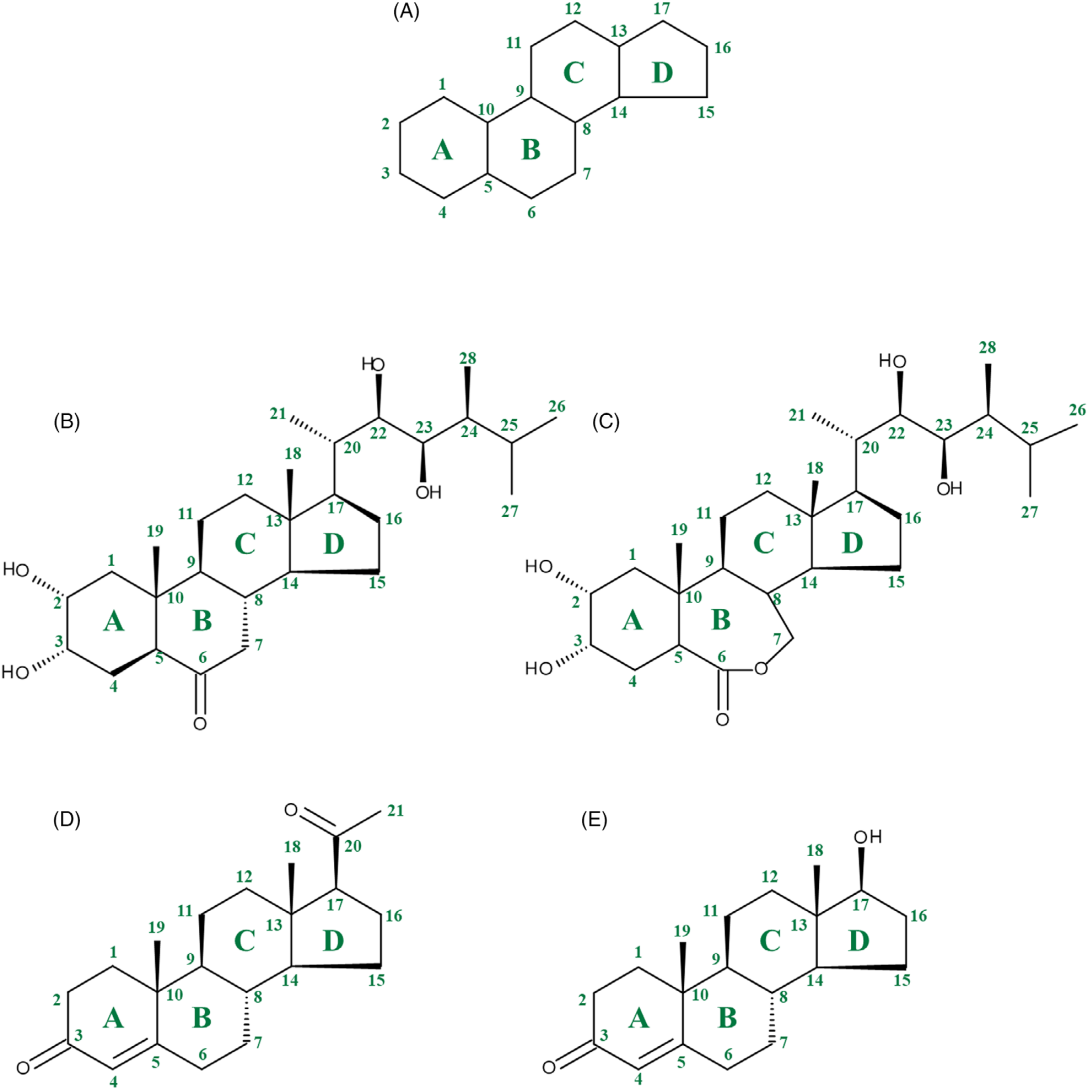
Chemical structures of sterane (A), and representative brassinosteroids, progestogen and androgen. The chemical structures of the brassinosteroids castasterone (B) and brassinolide (C) are also illustrated as are the structures of the progestogen progesterone (D) and the androgen testosterone (E). Carbon numbering and ring annotation (shown in green) follow IUPAC.

Steroids are lipophilic molecules, generally not water‐soluble and structurally defined as tetracyclic triterpenoids with a sterane backbone (Figure 1). They play a crucial role in controlling cell membrane flexibility across eukaryotic organisms. In plants, brassinosteroids (BRs), such as castasterone (CS) and brassinolide (BL), function as phytohormones involved in their growth and development, stress responses and biochemical processes (Peres et al., 2019). BR biosynthetic pathways are highly conserved among land plants, originating from phytosterols (Vriet et al., 2013; Klein et al., 2025).

The presence of mammalian‐like steroids, including progestogens and androgens, has been reported in plants for over 60 years (Bennett & Heftmann, 1965; Gawienowski & Gibbs, 1968; Bennett et al., 1969; Simons & Grinwich, 1989; Shiko et al., 2023; reviewed in Klein, 2024). Progesterone, for example, serves as a precursor for pharmaceutically valuable 5β‐cardenolides (Kreis, 2017; Leykauf et al., 2023; reviewed in Klein, 2024). Recent studies confirmed that progestogens and androgens are widespread in land plants, independent of their ability to produce cardenolides (Shiko et al., 2023).

Unlike BRs, the signalling pathways and physiological roles of these mammalian‐like steroids in plants are largely unknown. While the *Arabidopsis* genome lacks close homologues to animal nuclear steroid receptors, evidence suggests alternative steroid signalling mechanisms in plants (Bishop & Koncz, 2002). A noteworthy finding is the identification of *Arabidopsis* membrane steroid‐binding protein 1 (MSBP1), which binds to various steroids, including progesterone and brassinolide, influencing BR responses and root growth (Yang et al. 2005; reviewed in Li et al., 2022).

Due to the absence of well‐defined signalling pathways, most insights into plant steroid functions derive from exogenous applications followed by physiological assessments. Progesterone, for instance, has been shown to affect root and shoot growth in *Arabidopsis* and other species at concentrations of 0.01 to 1 μM (Erdal & Dumlupinar, 2011; Iino et al., 2007). In addition, some studies indicate a role in stress responses, such as reducing necrosis caused by pathogens (Janeczko et al., 2013).

**Figure 2:**
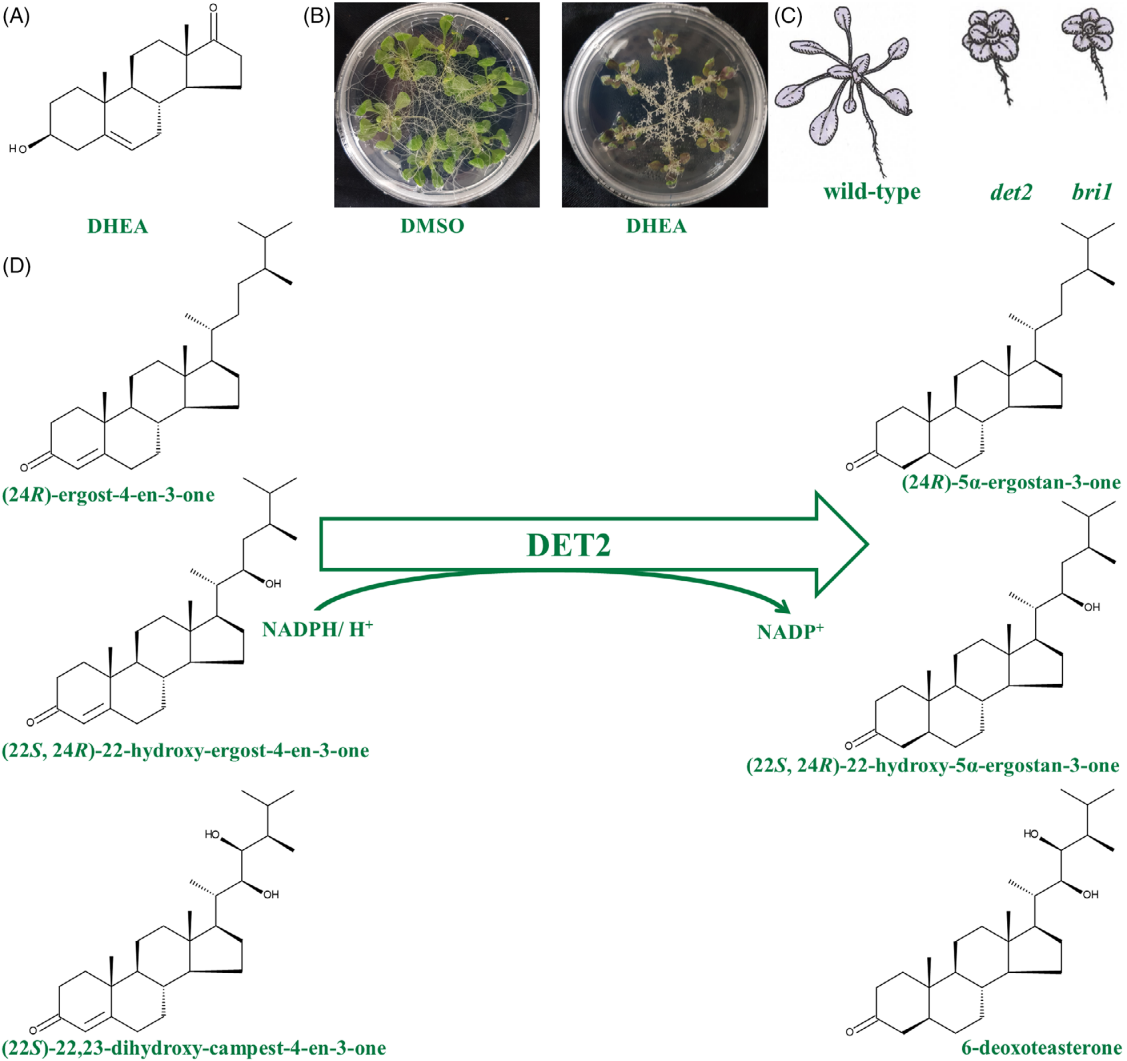
DHEA‐induced changes in root morphology and hypothesis of its mechanism of action. DHEA (A) is a typical C_21_ steroid. (B) MS medium supplemented with 0.03 mM androgen DHEA was shown to cause severe changes in root morphology of *Arabidopsis thaliana* (Oktay et al., 2025). (C) These morphological changes were compared with *A. thaliana* mutants deficient in brassinosteroid biosynthesis (e.g. DET2) or signalling (e.g. BR1). Drawings were prepared according to the pictures of Xu et al. (2019). (D) The fact that DET2 is essential for brassinosteroid production and is involved in the conversion of progestogens and androgens leads to the hypothesis that progestogens and androgens are competitive inhibitors of DET2. This could be used to fine‐tune of endogenous brassinosteroid concentrations.

Oktay et al. (2025) observed significant effects of dehydroepiandrosterone (DHEA) on *Arabidopsis* root morphology (Figure 2A), suggesting possible interference with BR biosynthesis or signalling. Given the structural similarities among steroids, DHEA may competitively inhibit DET2, a key enzyme in BR biosynthesis (Noguchi et al., 1999), thereby altering root growth patterns (Figure 2B). This enzyme's evolutionary conservation across eukaryotes implies that plant steroid 5α‐reductase shares functional similarities with its animal counterparts (Ali et al., 2024).

Despite being traditionally considered animal hormones, the detection of steroid‐binding proteins in plants raises the possibility that progestogens and androgens may act as signalling molecules in plants. This study addresses the following questions:

1. Is the effect of DHEA on root morphology specific to this androgen, or do other plant‐identified progestogens and androgens produce similar outcomes (Shiko et al., 2023)?
2. Is the influence of these steroids on root morphology unique to *Arabidopsis*, or is it observed in other angiosperms as well?
3. Are these effects mediated by alterations in BR biosynthesis and signalling, or do progestogens and androgens function independently as signalling molecules or even as phytohormones in plants?

## RESULTS

### The enzymatic machinery of progesterone biosynthesis is found in all angiosperms

**Figure 3:**
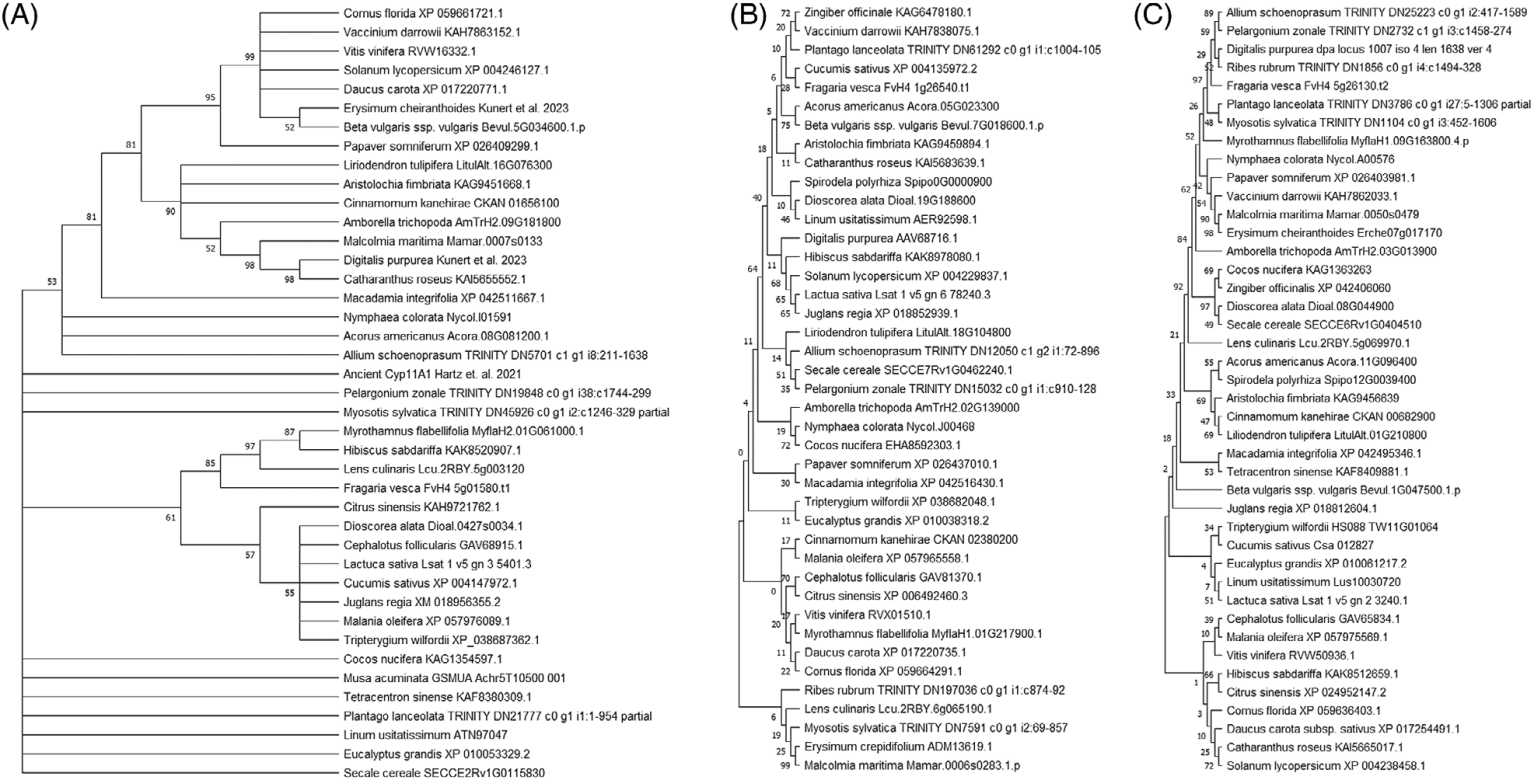
Evolutionary analysis by maximum likelihood method of sterol side‐chain cleavage enzymes, 3β‐hydroxysteroid dehydrogenases and ketosteroid isomerases in angiosperms. Evolutionary analyses were conducted in MEGA11 (Tamura et al., 2021). The evolutionary history of sterol side‐chain cleavage enzymes (SCCEs; A), 3β‐hydroxysteroid dehydrogenases (3β‐HSDs; B) and ketosteroid isomerases (KSIs, C) were inferred by using the maximum likelihood method and JTT matrix‐based model (Jones et al., 1992). The bootstrap consensus trees inferred from 1000 replicates (Felsenstein, 1985) are taken to represent the evolutionary history of the taxa analysed. Branches corresponding to partitions reproduced in less than 50% bootstrap replicates are collapsed. Initial tree(s) for the heuristic search were obtained automatically by applying Neighbour‐Join and BioNJ algorithms to a matrix of pairwise distances estimated using the JTT model and then selecting the topology with superior log likelihood value. (A) The analysis of SCCEs involved 41 amino acid sequences. This includes 40 plant SCCEs and the estimated ancestral CYP11A1 of mammalians designed by Hartz et al., 2021. There was a total of 494 positions in the final dataset. (B) The analysis of angiosperm 3β‐HSDs involved 41 plant amino acid sequences. There was a total of 352 positions in the final dataset. (C) The analysis of angiosperm KSIs involved 42 amino acid sequences. There was a total of 514 positions in the final dataset.

A pathway for progesterone biosynthesis in plants has recently been discovered (Herl et al., 2007; Munkert et al., 2014; Meitinger et al., 2015; Meitinger et al., 2016; Carroll et al., 2023; Kunert et al., 2023; Leykauf et al., 2023; Younkin et al., 2024; reviewed in Klein, 2024). First, we observed that these enzymes are not restricted to plants that produce cardenolides via PO (Figure 3). Moreover, SCCEs (Figure 3A), 3β‐HSDs (Figure 3B) and KSIs (Figure 3C) can be found in all orders of angiosperms where sequence data are available. These include the basal orders of angiosperms, monocots and eudicots. Nevertheless, we must mention that we could not identify SCCEs (CYP87A4‐like proteins) within *Spirodela polyrhiza*, *Zingiber officinalis*, *Borago officinalis* and *Ribes rubrum*.

Following the Arabidopsis eFP Browser (https://bar.utoronto.ca/efp/cgi‐bin/efpWeb.cgi [25 February 2025]), these three enzymes are expressed with an enhanced intensity in roots compared with shoots (SCCE [At1g12740]: total expression level of 1 in shoots, while 24 in roots; 3β‐HSD [At2g47140]: total expression level of 7 in shoots, while 74 in roots; KSI [At2g33630]: total expression level of 131 in shoots and 404 in roots). This nicely fits to the steroid levels in *Arabidopsis thaliana*. Within this study, we found 8 times higher levels of progestogens and androgens (the conversion of progestogens into androgens is conserved in plants [Shiko et al., 2023]) in *Arabidopsis thaliana* roots compared with *Arabidopsis thaliana* shoots (Table S8).

Therefore, we asked if progestogens and androgens have a physiological influence on root development and root morphology. We determined progesterone (PO) and 5α‐dihydroprogesterone (5α‐DHP) combined in a concentration of 3.4 ng mg^−1^ dry weight in 18‐day‐old *A. thaliana* roots. Estimating that 95% of a plant cell is water, this would be a total of 0.17 ng mg^−1^ progestogens in fresh root material. Testosterone (TO) and 5α‐dihydroprogesterone (5α‐DHT) were detected in combined levels of 1.5 ng mg^−1^ in *A. thaliana* roots (dry weight), which leads to an estimated amount of 0.075 ng mg^−1^ fresh weight (Data can be found in the supporting information; Table S8). We have taken a conscious decision to use higher concentrations of progesterone (3.1–9.4 μg mL^−1^ = 10–30 μM) and testosterone (2.9–8.6 μg mL^−1^ = 10–30 μM) within this study to disturb steroid homeostasis and analyse the effects. This strategy is often used in the literature. Exemplarily, the phytohormone concentrations in *A. thaliana* (Meents et al., 2019) differ strongly from those used for plant treatment (see exemplarily the concentrations used in the Arabidopsis eFP Brower; https://bar.utoronto.ca/efp/cgi‐bin/efpWeb.cgi?dataSource=Hormone [12 May 2025]).

To ensure that the effects are caused by these both steroidal substances, the uptake of PO and TO was analysed by quantification of steroid levels in *A. thaliana*. The amount of progestogens and androgens in total raised after an 8‐day progesterone treatment from 5.82 ng mg^−1^ dry weight to 4600 ng mg^−1^ dry weight, while an 8‐day treatment with testosterone resulted in total progestogen and androgen values of 130 ng mg^−1^ dry weight. These data are given in the supporting information (Table S8).

### Progestogens and androgens change the root morphology in various angiosperms

**Figure 4:**
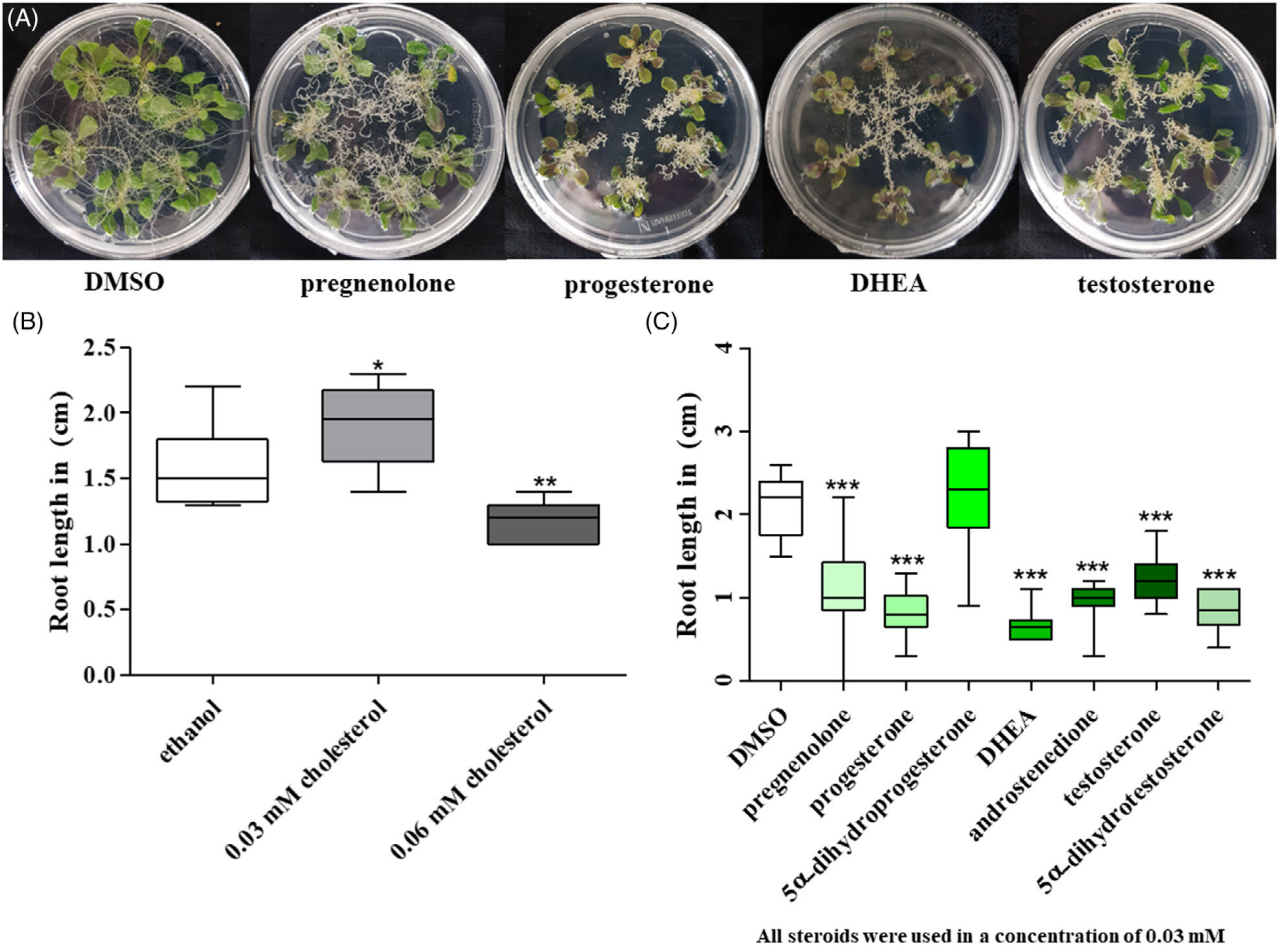
Effects of progestogens and androgens on root length of *A. thaliana*. Cholesterol was dissolved in ethanol (25 mM stock solutions), while steroids were dissolved in DMSO (25 mM stock solutions). Cholesterol and steroid solutions were added to MS medium under sterile conditions (final concentration: 0.03 mM). MS medium supplemented with pure EtOH or DMSO was used as a mock treatment. Six 10‐day‐old plants were transferred on one round petri dish (⌀ = 8.5 cm), and the root morphology was detected after an additional 7 days. (A) Roots of DMSO treated control, pregnenolone, progesterone, DHEA and testosterone. (B) To analyse the effects of cholesterol on root length, 10 *A. thaliana* seeds were sown on square plates (12 × 12 cm) MS medium supplemented with 0.03 or 0.06 mM cholesterol. (C) Steroids (including: 5α‐dihydroprogesterone and5α‐dihydrotestosterone) dissolved in DMSO were added to MS medium (final concentration = 0.03 mM). Root lengths of the longest root of a seedling in the parts F and G were determined after 9 days of culture. Data are given as mean ± SEM; *n* ≥ 8; *P* ≤ 0.001; ANOVA test was performed using Bonferroni correction. *P* < 0.05: *; *P* < 0.01: **; *P* < 0.001: ***

The occurrence of PO biosynthesis genes and the conserved mechanism for the conversion of progestogens (e.g. PO) into androgens (e.g. TO) raise the question of whether these steroids have a conserved function in plants. As reported previously, Oktay et al., 2025 showed a strong root reduction caused by DHEA. Therefore, we tested whether cholesterol, PR, PO, AD, TO, 5α‐DHP and 5α‐DHT resulted in the same effects (Figure 4).

Cholesterol (0.03 mM) lead to a slight, but significant enhancement of the root length of *A. thaliana* (120% root length compared with the ethanol control), while treatment with a concentration of 0.06 mM resulted in a slightly reduced root length (74% compared with ethanol control).

All progestogens and androgens (except 5α‐DHP) led to a significantly reduced root length (PR: 47% root length; PO: 31.5% root length; AD: 37.8% root length; DHEA: 25.7% root length; 47.5% root length; 5α‐DHP: 86.0% root length; 5α‐DHT: 32.3% root length). Additionally, a significant, but smaller decrease in root length could be detected for the oestrogen estradiol (ER). After a treatment with 30 μM estradiol, *Arabidopsis* roots still showed 84% of the root length of the mock‐treated control (Figure S1).

The dose‐dependent effects of progestogens, androgens and oestradiol on the root length of *Arabidopsis thaliana* can be found in the Figure S1.

**Figure 5:**
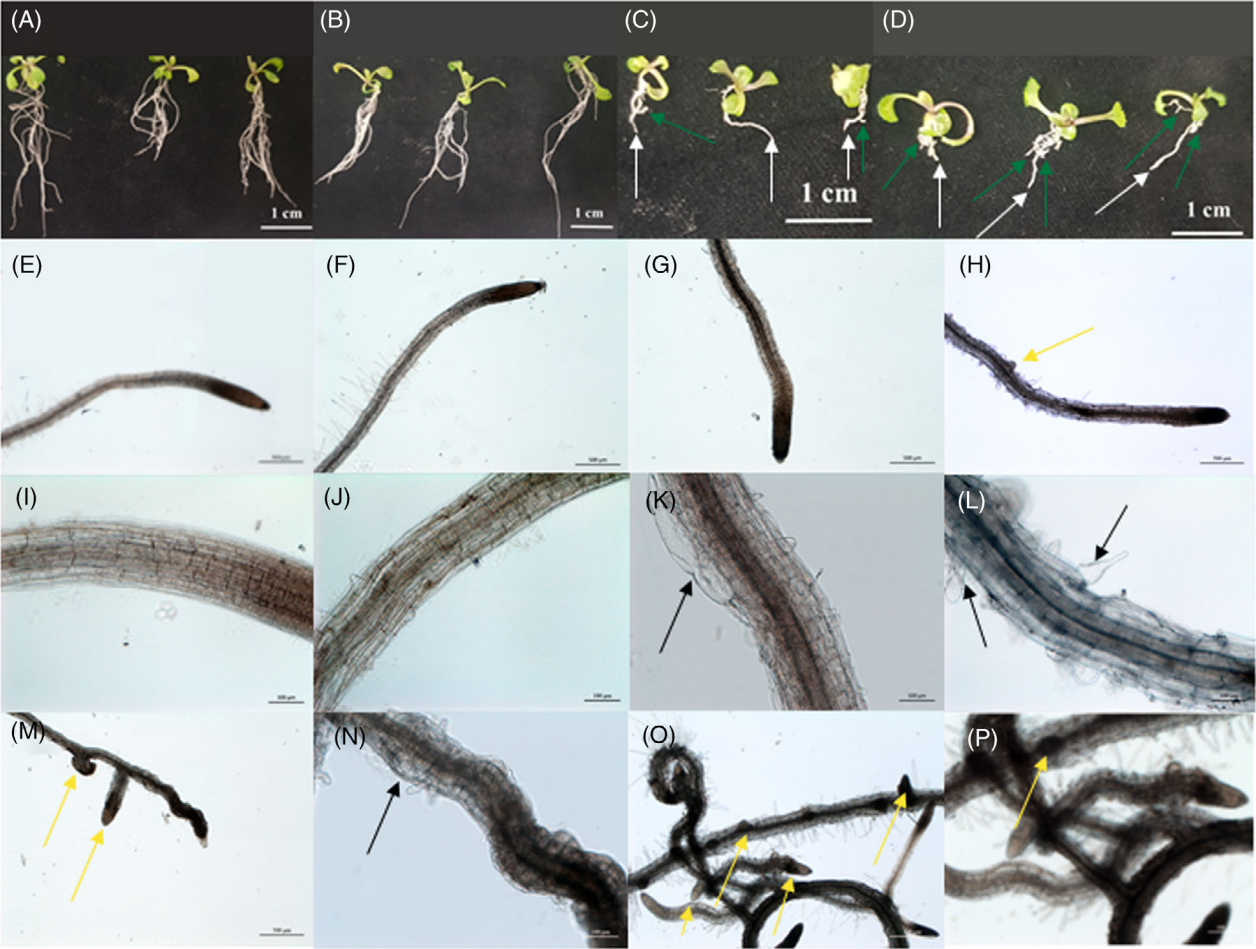
Effects of progestogens and androgens on root morphology of *A. thaliana*. *A. thaliana* plants germinated on MS medium supplemented with progesterone and testosterone showed reduced root length and altered root morphology. The figure shows 14 –day‐old *A. thaliana* seedlings germinated on MS medium (A), MS medium and DMSO as a control (B), MS medium supplemented with 0.03 mM progesterone (C) and 0.03 mM testosterone (D). Main (white arrows) and lateral (arrows in green) roots of progesterone‐ and testosterone‐treated seedlings are marked. A size indicator gives 1 cm (white). Microscopic analysis showed an uncoordinated growth of cells in progesterone‐ and testosterone‐treated roots (black arrows) and enhanced formation of lateral roots (yellow arrows). The figure shows the main roots of plants germinated on MS medium (E, I), MS medium with DMSO (F, J), MS medium containing 0.03 mM progesterone (G, K) and 0.03 mM testosterone (H, L). The effect was visible stronger on lateral roots of plants treated with progesterone (M, N) and testosterone (O, P). Size indicators for microscopic pictures are given in black or white. They are 500 μM for E, F, G, H, M and O and 100 μM for I, J, K, L, N and P.

Moreover, the applied steroids induced a root phenotype characteristic of this group of substances. This phenotype shows increased lateral root formation and uncoordinated cell growth. This effect was more pronounced in lateral roots (Figure 5).

We showed in 3.1 that enzymes needed for PO biosynthesis are strongly conserved in angiosperms, and we found them in nearly all analysed angiosperms (except *B. officinalis*, *R. rubrum*, *S. polyrhiza* and *Z. officinale*). This is why the presence of PO could be found in a broad range of angiosperms (Simons & Grinwich, 1989; Shiko et al., 2023), and also, the conversion of PO into TO seems to be conserved (Shiko et al., 2023). Additionally, the occurrence of progestogens and androgens were shown for a moiety of angiosperms (Simons & Grinwich, 1989; Shiko et al., 2023). This indicates an evolutionary pressure on progestogen/androgens biosynthesis. We wondered, if a conserved physiological function in plants causes this pressure. That is why we analysed the effect of PO and TO on root development of 13 additional *Brassicacea* species (Table 1; Figure S2).

**Table 1:** Effect of progesterone and testosterone on root morphology of *Brassicacea* species.

Species	Further information	Effect of PO	Effect of TO	PO biosynthesis genes available
*Arabidopsis thaliana* (L.) HEYNH.	Figures 4 and 5	RRL	RRL	Species
*Aurinia saxatilis* (L.) DESV.	Figure S2A	RRL	RRL	Family
*Barbarea vulgaris* W.T.Aiton	Figure S2B	RRL	RRL	Family
*Brassica oleracea* convar. *capitata* var. *rubra* L. cv. Schwarzkopf	Figure S2C	RRL	RRL	Species
*Camelina sativa* (L.) CRANTZ	Figure S2D	–	–	Species
*Cochlearia officinalis* L.	Figure S2E	–	–	Family
*Diplotaxis tenuifolia* (L.) DC.	Figure S2F	RRL	RRL	Family
*Eruca vesicaria* subsp. *sativa* (L.) CAV.	Figure S2G			Species
*Erysimum cheiri (L.) CRANTZ	Figure S2H	RRL	RRL	Genus
*Erysimum crepidifolium RCHB.	Figure S2I	RRL	RRL	Species
*Hesperis matronalis* L.	Figure S2J	–	–	Family
*Isatis tinctoria* L.	Figure S2K	RRL	RRL	Species
*Lepidium sativum* L.	Figure S2L	ERL		Species
*Lobularia maritima* (L.) DESV. cv. Schneeteppich	Figure S2M	–		Family
*Malcolmia maritima* (L.) W.T. AITON	Figure S2N			Species
*Matthiola incana* (L.) W.T.AITON	Figure S2O	RRL	RRL	Family
*Nasturtium officinale* W.T.AITON	Figure S2P	RRL	RRL	Family
*Raphanus sativus* var. *sativus* L. cv. Riesenbutter	Figure S2Q	RRL	RRL	Family
*Sinapsis alba* L.	Figure S2R	RRL	RRL	Species
*Sisymbrium officinale* (L.) SCOP.	Figure S2S	RRL	RRL	Genus

A strong effect of progesterone (PO) and testosterone (TO) on root morphology of *A. thaliana* was found. The occurrence of biosynthesis genes (Figure 4) and steroids (Shiko et al., 2023) raised the question of how strongly this effect is conserved. From this reason, we germinated 13 additional *Brassicacea* species on MS medium containing 30 μM progesterone or 30 μM testosterone. Whenever possible, experiments were conducted with edible or medicinal plant species. We analysed the effects in comparison with pure MS medium and MS medium supplemented with DMSO. Rooth lengths, pictures of the seedlings and microscopic images of root tips can be found in the supplement (Figure S2A–S). Species containing cardenolides are marked by an asterisk.

ERL, enhanced root length compared with DMSO control; Family, PO biosynthesis enzyme sequences are available in species of the same family; Genus, PO biosynthesis enzyme sequences are available in species of the same genus; Order, PO biosynthesis enzyme sequences are available in species of the same order; RRL, reduced root length compared to DMSO control; Species, PO biosynthesis enzyme sequences of this species are available.

Effects of PO and TO on root length were observed in most of the *Brassicaceae* species analysed (14 of 20 for PO; 13 of 20 for TO; Table 1; Figure S2). These include the wallflower species *Erysimum crepidifolium* and *Erysimum cheiri*, which form 5β‐cardenolides from progesterone. Interestingly, *Malcolmia maritima* from the genus *Malcolmia* (the sister genus of wallflowers) did not show any shortened root length in response to PO and TO, but morphological changes could also be detected in *Malcolmia maritima* (unstructured cell growth in the area of the cell elongation zone; Figure S2N). In our experiments, four of the six Brassicaceae species that do not show a reduced root length in response to PO or TO (*Cochlearia officinalis*, *Eruca vesicaria* subsp. *sativa*, *Lobularia maritima* and *Malcolmia maritima*) have in common that even untreated plants show the formation of root hairs comparable near to the root tip (Figure S2E,G,M,N). This is not the case for *Camelina sativa* (gold‐of‐pleasure) and *Hesperis matronalis* (dame's rocket). If the modus of root hair formation is connected to the physiological effects of PO and TO in plants, it has to be unravelled in further studies. Data on root length, seedling morphology and root tips of all analysed Brassicaceae species can be found in Figure S2.

Thus, we found that the negative effect of steroids is highly conserved in *Brassicaceae* species; we wanted to analyse whether it is possible to observe a comparable effect in other angiosperms outside the order Brassicales. Therefore, we analysed the consequences of PO and TO on root development of 23 non‐Brassicales species from 22 angiosperm orders (Table 2; Figure S3). Two Plantaginaceae species were analysed to compare the effects of close relatives (both members of the *Plantaginaceae* family, order Lamiales), which are cardenolide producers (*Digitalis purpurea* L.) or not (*Plantago major* L.).

**Table 2:** Effect of progesterone and testosterone on root morphology in non‐Brassicales angiosperm species.

Supporting information		Systematics	Species	Effect of PO	Effect of TO	PO biosynthesis genes available
Basal orders	Figure S3A	Nymphaeales Nymphaeaceae	*Nymphaea colorata* PETER.	RRL	RRL	Species
	Figure S3B	Alismatales *Lemnaceae*	*Spirodela polyrhiza* (L.) SCHLEID. clone 9256	RRL	RRL	Species, but no SCCE homologue could be detected
Monocots	Figure S3C	Asparagales *Amaryllidaceae*	*Allium schoenoprasum* L. cv. Nelly	RRL	–	Species
	Figure S3D	Poales *Poaceae*	*Secale cereal* L. cv. Dukato	RRL	RRL	Species
– Eudicots	Figure S3E	Apiales Apiaceae	*Petroselinum crispum* (MILL.) FUSS cv. Mooskrause	RRL		Family
	Figure S3F	Asterales *Asteracea*	*Taraxacum officinale* L. – wild population Dittelbrunn	–	–	Family
	Figure S3G	Boraginales Boraginaceae	*Myosotis sylvatica* EHRH. EX HOFFM. cv. Heavenly Blue	–	–	Species
	Figure S3H	Caryophyllales *Amaranthaceae*	*Beta vulgaris subsp. vulgaris CONDITIVA* group L.	–	–	Species
	Figure S3I	Cucurbitales *Cucurbitaceae*	*Cucumis sativus* L. cv. Vorgebirgstraube	RRL		Species
	Figure S3J	Dipsacales *Caprifoliaceae*	*Valerianella carinata* LOISEL.	RRL	RRL	Order
	Figure S3K	Ericales *Ericaceae*	*Vaccinium myrtillus* L.	–	–	Genus
	Figure S3L	Fabales *Fabaceae*	*Trigonella foenum‐graecum* L.	RRL	ERL	Family
	Figure S3M	Gentianales *Gentianaceae*	*Centaurium erythraea* RAFN.	RRL	RRL	Order
	Figure S3N	Geraniales *Geraniaceae*	*Pelargonium zonale* (L.) L'Hér.	RRL	–	Species
	Figure S3O	Lamiales *Plantaginaceae*	*Digitalis purpurea L.	–	–	Species
	Figure S3P	Lamiales *Plantaginaceae*	*Plantago major* L. – wild population Dittelbrunn	RRL	RRL	Genus
	Figure S3Q	Malpighiales *Linaceae*	*Linum usitatissimum* L.	RRL	–	Species
	Figure S3R	Malvales Malvaceae	*Hibiscus sabdariffa* L.	–	–	Species
	Figure S3S	Myrtales *Onagraceae*	*Oenothera speciosa* NUTT.	–	–	Order
	Figure S3T	Ranunculales *Papaveraceae*	*Papaver rhoeas* L.	RRL	RRL	Genus
	Figure S3U	Rosales *Rosaceae*	*Fragaria vesca* L. cv. Rügen	–	–	Species
	Figure S3V	Sapindales *Rutaceae*	*Ruta graveolens* L.	–	–	Family
	Figure S3W	Solanales *Solanaceae*	*Solanum lycopersicum* L. cv. Harzfeuer	RRL		Species

A strong effect of progesterone (PO) and testosterone (TO) on root morphology of *Brassicacea* was detected. The occurrence of biosynthesis genes (Figure 4) and steroids (Shiko et al., 2023) raised the question of whether this effect is conserved within non‐*Brassicales* angiosperms. Therefore, we germinated 23 additional non‐Brassicales species on MS medium containing 30 μM progesterone or 30 μM testosterone. Whenever possible, the experiments were conducted with edible or medicinal plant species, or plants available in wild populations in Germany (Dittelbrunn, Bavaria, Germany). We analysed the effects compared with pure MS medium and MS medium supplemented with DMSO. Root lengths, pictures of the seedlings and microscopic images of root tips can be found in the Figure S3. Species containing cardenolides are marked by an asterisk.

ERL, enhanced root length compared with DMSO control; Family, PO biosynthesis enzyme sequences in species of the same family are available; Genus, PO biosynthesis enzyme sequences in species of the same genus are available; NDA, No PO biosynthesis enzyme sequences of species in this order are available; Order, PO biosynthesis enzyme sequences in species of the same order are available; RRL, reduced root length compared with DMSO control; Species, PO biosynthesis enzyme sequences of this species are available.

The effects of progestogens and androgens on non‐Brassicales angiosperms' root morphology are more complicated than on *Brassicaceae* species. Data on root length, seedling morphology and root tips of all analysed Brassicaceae species can be found in Figure S3. While 60% (14 of 23) of the analysed non‐Brassicales angiosperms showed reduced root length when treated with PO (Table 2; Figure S3), only 7 species (*Nymphaea colorata*, Nymphales, basal orders of angiosperms; *Spirodela polyrhiza*, Alismatales, Monocot; *Secale cereale* cv. Dukato, Poales, Monocot; *Valerianella carinata* LOISEL., Dipsacales, Eudicot; *Centaurium erythraea* RAFN., Gentianales, Eudicot; *Plantago major* L., Lamiales, Eudicot; *Papaver rhoeas* L., Ranunculales, Eudicot) showed reduced root length in response to TO. We conclude that the species outside the Brassicales respond more strongly to PO compared with TO and that the TO response is somewhat species‐dependent. Interestingly, some non‐Brassicales plants that did not show a significant change in root length had altered root morphology. For example, cucumber (*Cucumis sativus* L. cv. Vorgebirgstraube) showed typical uncoordinated cell growth in the regions of cell elongation and the early root hair zone, as well as increased lateral root formation (Figure S3I) for both PO and TO treatment, but the effect was much stronger for PO compared with TO. Even the herb‐of‐grace (*Ruta graveolens* L.; Sapindales), an important medicinal plant and abortifacient since ancient times (Nelson, 2009), showed no differences in root length, but uncoordinated cell growth in PO‐treated plants, but not in TO‐treated plants (Figure S3V). Conversely, in fenugreek (*Trigonella foenum‐graecum* L.), TO treatment resulted in enhanced root length and promoted the formation of lateral roots. At the same time, PO‐treated plants showed reduced root length, but no additional morphological difference from controls (Figure S3L). Nevertheless, we detected non‐Brassicales species with reduced root length and altered root morphology (e.g. the corn salad species *Valerianella carinata* LOISEL. or the medicinal plant species *Centaurium erythraea* RAFN.), too.

Overall, we observed a conserved effect of progestogens and androgens on angiosperm root morphology. Nevertheless, the potency and activity of these steroids seem to be species‐dependent.

### Steroid 5α‐reductases metabolise progesterone and testosterone in plant roots, but morphological changes of *A. thaliana* roots are not caused by competitive inhibition of the steroid 5α‐reductase DET2 by exogenous progesterone and testosterone

Based on the effects of PO and TO on root morphology, we wanted to determine whether this effect is mediated by the action of exogenous PO or TO on endogenous BR levels in plants or whether these progestogens and androgens act in a BR‐independent manner. A putative way how PO and TO can have an impact on BR levels is by competitive inhibition of enzymes needed for BR biosynthesis. An essential enzyme of BR biosynthesis is DET2. The steroid 5α‐reductase DET2 is an enzyme highly conserved in eukaryotes (Li et al., 1997). Consequently, the SRD5A1, the steroid 5α‐reductase of *Homo sapiens* (mammals) and DET2 of *A. thaliana* (eudicots) can be exchanged. That means the expression of the human SRD5A1 in DET2 knock‐out lines cures the morphological effects of the DET2 knock‐out (Li et al., 1997).

**Figure 6:**
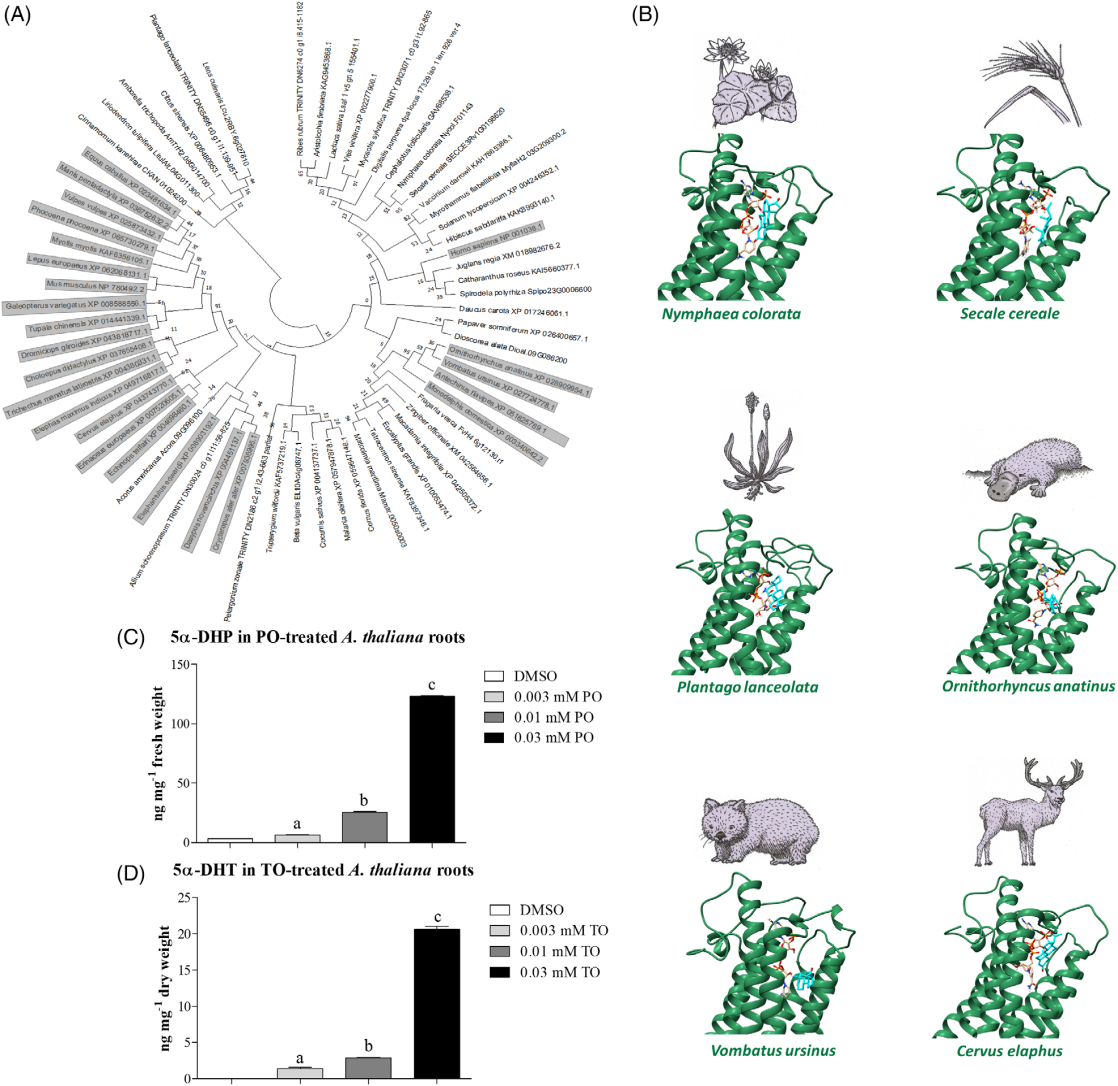
Steroid 5α‐reductases are strongly conserved in mammals and angiosperm plants, and PO‐ and TO‐treated *A. thaliana* roots accumulate 5α‐reduced steroid derivates. The figure depicts that steroid 5α‐reductases are strongly conserved in eukaryotes. (A) A bootstrap consensus tree shows the high conservation of steroid 5α‐reductases of plant and mammal origin (grey). This evolutionary history was inferred by using the maximum likelihood method and JTT matrix‐based model (Jones et al., 1992). The bootstrap consensus tree inferred from 1000 replicates (Felsenstein, 1985) represents the evolutionary history of the taxa analysed. Branches corresponding to partitions reproduced in less than 50% bootstrap replicates are collapsed. Initial tree(s) for the heuristic search were obtained automatically by applying Neighbour‐Join and BioNJ algorithms to a matrix of pairwise distances estimated using the JTT model and then selecting the topology with superior log likelihood value. This analysis involved 63 amino acid sequences (24 mammal sequences and 39 plant sequences). There was a total of 322 positions in the final dataset. Evolutionary analyses were conducted in MEGA11 (Tamura et al., 2021). (B) Moreover, the high grade of conservation is demonstrated here by a docking experiment. This shows that the position of progesterone in the binding pocket of steroid 5α‐reductases from *Ornithorhynchus anatinus* SHAW (Protheria), *Vombatus ursinus* (Metatheria) and *Cervus elaphus* (Eutheria) is very close to the position of progesterone in the binding pocket of steroid 5α‐reductases from *Nymphaea colorata* (Basal orders of angiosperms), *Secale cereale* (Monocots) and *Plantago lanceolata* (Eudicots). (C, D) In line with this, we show here that *A. thaliana* roots can convert progesterone or testosterone to 5α‐dihydroprogesterone and 5α‐dihydrotestosterone, respectively. A one‐way ANOVA ensured statistical differences between all used steroid concentrations. This was seen as a fact for the dose‐dependent conversion. The graph shows mean ± SEM, *n* = 3.

In mammals, steroid 5α‐reductases participate in steroidogenesis by the conversion of PO and TO into 5α‐DHP or 5α‐DHT (Normington & Russell, 1992; Sinnecker et al., 1996), while plant steroid 5α‐reductases participate in BR biosynthesis by the conversion of (24R)‐ergost‐4‐en‐3‐one, (22S, 24R)‐22‐hydroxy‐ergost‐4‐en‐3‐one or (22S)‐22,23‐dihydroxy‐campest‐4‐en‐3‐one into (24R)‐5α‐ergostan‐3‐one, (22S, 24R)‐22‐hydroxy‐5α‐ergostan‐3‐one or 6‐deoxoteasterone (Figure 2). In this study, we performed docking of DET2 homologues from basal orders of angiosperms (*N. colorata*), monocots (*S. cereale*) and dicots (*P. lanceolata*), as well as docking of mammal steroid 5α‐reductases from Protheria (*Ornithorhynchus anatinus* SHAW.), Metatheria (*Vombatus ursinus* É. GEOFFROY SAINT‐HILAIRE.) and Eutheria (*Cervus elaphus* Linnaeus) with PO substrate and NADP co‐substrate (Figure 6B). First of all, we discovered that PO and its co‐substrate are localised and bound in very similar ways in plant and animal enzymes. This is documented by comparable, empirical binding free energies for the docking with PO (angiosperms: between −5.6 and − 7.8 kcal mol^−1^; mammals: between −3.9 and − 8.9 kcal mol^−1^) and the docking with the co‐substrate (angiosperms: between −10.5 and − 11.9 kcal mol^−1^; mammals: between −11 and − 11.3 kcal mol^−1^). Moreover, we could see familiar distances between the substrate and the co‐substrate (angiosperms: between 4.3 and 6 Å; mammals: between 4.9 and 6.7 Å). Therefore, we are confident that all functional DET2 homologues from angiosperms can reduce PO to 5α‐DHP. It is very likely that a similar conversion also occurs in the case of TO.

Secondly, we analysed if steroid 5α‐reductase activity of plant roots can convert PO or TO when these steroids are applied exogenously. As shown here (Figure 6C,D), we confirmed the presence of steroid 5α‐reductase activity in the roots of *A. thaliana*. This effect is dose‐dependent, leading to the formation of 5α‐DHP and 5α‐DHT, respectively. These data could support the theory that PO and TO alter root morphology by competitively inhibiting DET2, leading to subsequent inhibition of BR biosynthesis. However, recalling that steroid 5α‐reductases are highly conserved in angiosperms (Figure 6A,B), the question arises as to why the effect of PO and TO on root morphology is less conserved (see Table 2).

**Figure 7:**
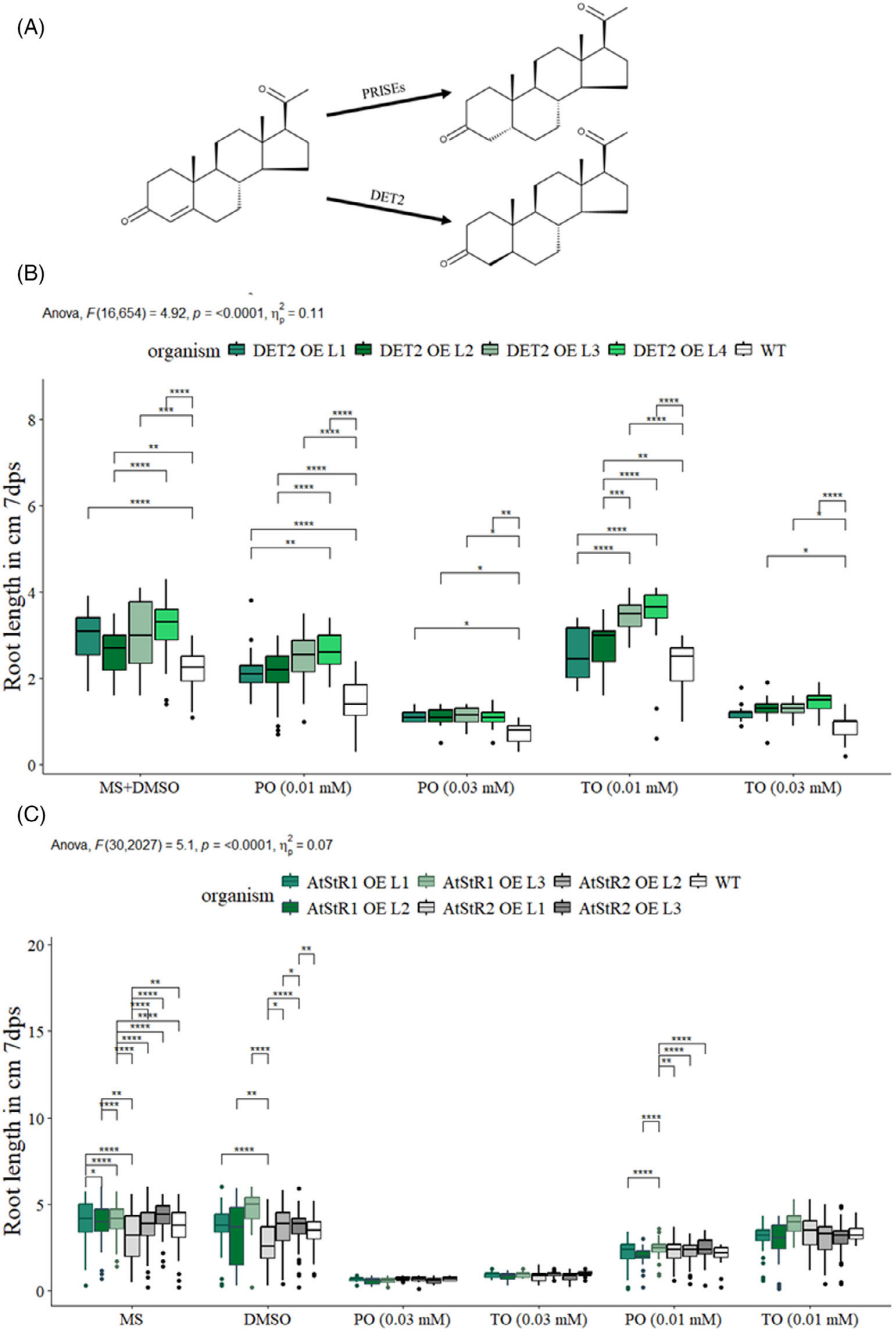
Root length of *A. thaliana* lines overexpressing steroid 5α‐reductases or steroid 5β‐reductases (PRISEs). (A) Plants can convert progestogens and androgens via both the 5α‐ and 5β‐reduction pathways. To challenge the hypothesis that the competitive inhibition of DET2 causes impaired root morphology in *A. thaliana*, we overexpressed both the steroid 5α‐reductase DET2 and the steroid 5α‐reductases AtStR1 and AtStR2 in *A. thaliana*. (B) We found that all four DET2 overexpressing lines analysed had significantly longer roots compared with wild‐type plants. This can also be observed in individuals treated with progesterone or testosterone, but the relation between mock and steroid treatment is not altered. Data are given as mean ± SEM; *n* ≥ 8; *P* ≤ 0.001; ANOVA test was performed with Bonferroni correction. *P* < 0.05 : *; *P* < 0.01: **; *P* < 0.001: ***; *P* < 0.0001: **** (C) *A. thaliana* lines overexpressing AtStR1 or AtStR2 did not show altered root length upon mock and steroid treatment. Root lengths were determined after 7 days of culture. Data are given as mean ± SEM; *n* ≥ 8; *P* ≤ 0.001; ANOVA test was performed with Bonferroni correction. *P* < 0.05 : *; *P* < 0.01: **; *P* < 0.001: ***; *P* < 0.0001: ****

Finally, to challenge the hypothesis that PO or TO treatment causes competitive inhibition of DET2 leading to the observed phenotypes, we focused on the model plant *A. thaliana*. We designed lines overexpressing DET2 (progesterone 5α‐reductase; cloning and transformation can be found in the Figure S4) as well as AtStR1 and AtStR2 (progesterone 5β‐reductases; PRISEs; see exemplarily: Klein, Horn, et al., 2021; Klein, Ernst, et al., 2021; Dorfner et al., 2024). We hypothesised that overexpression of both classes of enzymes might reduce or even cure the effects of PO and TO treatment on *A. thaliana* roots. The transformation strategies can be found in Figure S3. Here, we present data of 4 independent lines overexpressing DET2 and 3 lines overexpressing AtStR1 or AtStR2 (Figure 7).

While the overexpression of DET2 resulted in longer roots compared with wild‐type plants (Figure 7B), DET2 nor PRISEs overexpression had a positive or even curative effect on root length in *A. thaliana* treated with PO or TO. We have to explain: We cannot exclude a competitive inhibition of DET2 by PO or TO (e.g. in *in vitro* assays), but we conclude that a competitive inhibition of DET2 does not cause the effect of exogenously applied PO or TO. Nevertheless, there are multiple substrate‐promiscuous enzymes within the BR biosynthesis pathway. Therefore, PO and TO treatment could still influence the BR profiles of *A. thaliana* roots. Therefore, we analysed the BR profiles of PO‐ and TO‐treated *A. thaliana* plants.

### Progesterone and testosterone treatment did not alter brassinosteroid profiles in *A. thaliana* in a manner indicative of brassinosteroid deficiency

**Figure 8:**
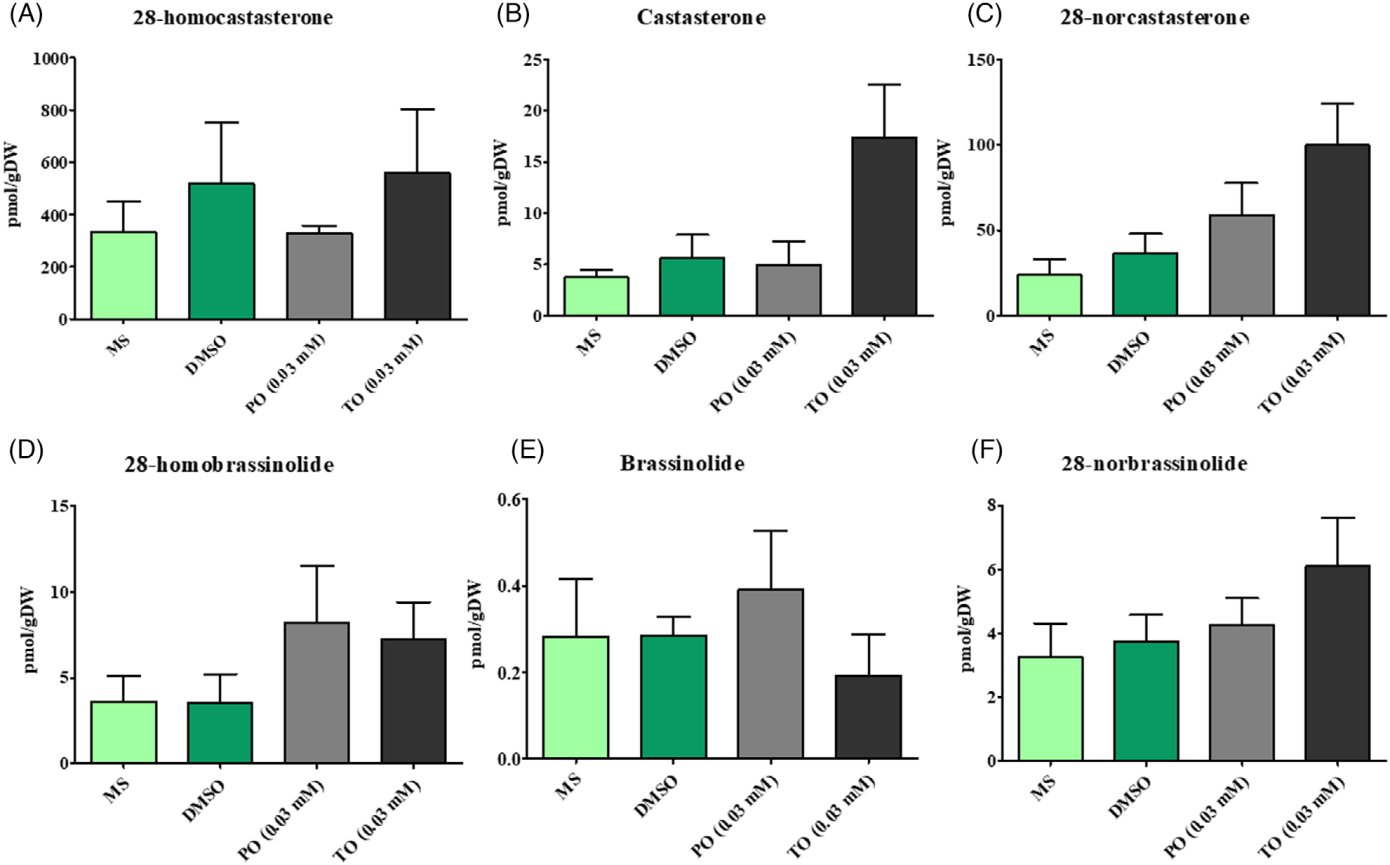
Brassinosteroid profiles of 14‐day‐old *A. thaliana* roots after 4 days of progesterone and testosterone treatment. Here, we show the contents of the endogenous brassinosteroid 28‐homocastasterone (A), castasterone (B), 28‐norcastasterone, 28‐homobrassinolide (D), brassinolide (E), and 28‐norbrassinolide (F) in *A. thaliana* roots after 4 days of with progesterone and testosterone treatment compared with mock and untreated control. The graph shows mean ± SEM (*n* ≥ 3).

Based on the effects of PO and TO on root morphology, we further wanted to determine whether this effect is mediated by the action of exogenous PO or TO on endogenous BR levels in plants or whether these progestogens and androgens act in a BR‐independent manner. Since BR biosynthesis involves several promiscuous enzymes of BR precursor biosynthesis which could also convert progestogens and androgens, we additionally analysed the effects of PO and TO on endogenous BR profiles in *A. thaliana* roots (Figure 8).

We did not observe any significant differences between steroid‐treated roots and controls, and even the total amount of brassinosteroids (the sum of all measured brassinosteroids) was not significantly different. This was interpreted as an indication that progestogens and androgens do not affect BR biosynthesis. Additionally, *A. thaliana* lines overexpressing DET2 were also analysed in response to PO and TO, but no significant changes in endogenous brassinosteroid profiles were detected (Table S4). The raw data and statistical analysis of BR analysis in *A. thaliana* wild‐type plants can be found in the Table S7.

### RNAseq analysis of progesterone and testosterone‐treated *A. thaliana* shoots did not show any alteration in the *A. thaliana* transcriptome caused by BR deficiency or BR excess

To determine whether PO and TO act as disruptors of BR homeostasis, we analysed the gene expression of *A. thaliana* shoots treated for 1 h with PO and TO compared with DMSO (mock treatment) using RNAseq (differentially expressed genes, DEGs, were listed in the Tables S5 and S6). To obtain statistically robust results by GO‐term analysis, two minimum requirements were implemented per gene locus (DEG data): a *P*‐value below 0.05 and a log foldchange (logFC) value outside the range of [−1, 1].

**Figure 9:**
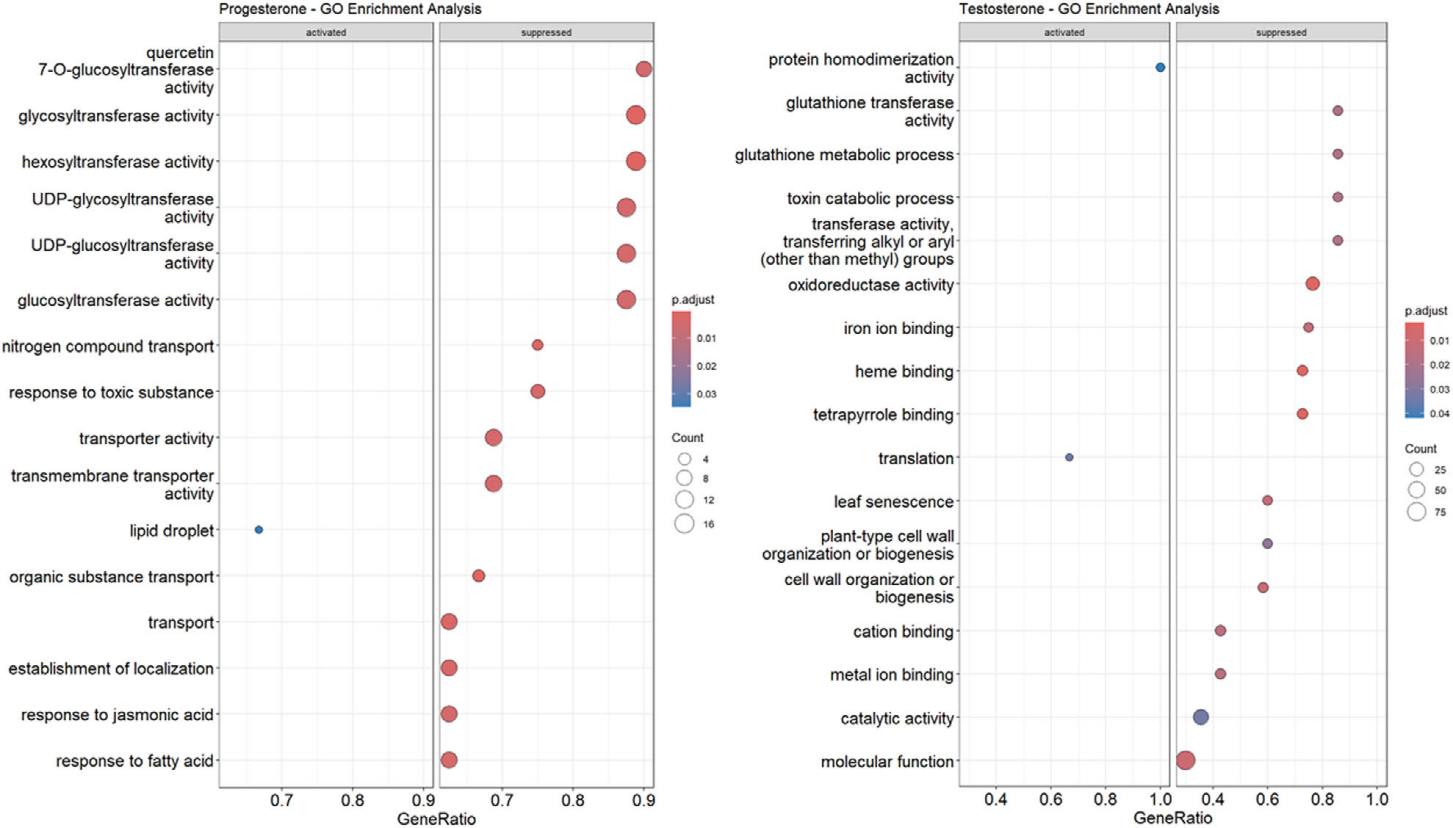
Transcriptomic changes in *A. thaliana* shoots induced by progesterone or testosterone. The figure shows the combined RNA sequencing results of four biological replicates.

One hour of PO treatment resulted in the suppression of various genes that are annotated to various glucosyl‐, hexosyl‐ and glycosyltransferases, indicating major alterations of sugar metabolic pathways in plants. In addition, genes important for jasmonic acid (JA) and fatty acid responses were suppressed. The strong effect of PO on root morphology also indicates a strong effect of this steroid on transport processes in plants. As a result, the expression of genes associated with transport, especially genes associated with organic matter transport and nitrogenous compound transport genes, was suppressed (Figure 9A). GO‐term enrichment analysis of TO treatment produced different displayed GO terms, but again mostly suppressed ones. The significantly dysregulated GO terms of this treatment can be roughly outlined into three suppressed groups: ion binding, cell wall biogenesis and catalytic functions (Figure 9B).

Interestingly, PO and TO alter the *A. thaliana* shoot transcriptome in different ways. Therefore, it is debatable whether progestogens and androgens lead to reduced root length and altered root morphology by separate mechanisms, at least in *A. thaliana*. This could be due to disruption of transport mechanisms by PO, while the TO treatment leads to defects in cell wall formation.

As a second approach, we identified gene loci that were statistically significantly regulated (*P*‐value <0.05). The obtained data frame contained only 624 gene loci, which further processing divided into two subgroups classified by upregulation (286 gene loci) and downregulation (338 gene loci). The subsets were cut down to genes with known protein families. The remaining 107 upregulated gene loci, as well as 163 downregulated genes, were investigated by searching NCBI (https://www.ncbi.nlm.nih.gov/gene/ [15 July 2024]). Table 3 shows the gene loci with *P*‐values <0.05 and log foldchanges −1≤ or ≥1.

**Table 3:** Gene loci enhanced and suppressed by progesterone and testosterone treatment.

Enhanced expression in steroid‐treated shoots				Reduced expression in steroid‐treated shoots			
Gene loci	Gene name	logFCPO	logFCTO	Gene loci	Gene name	logFCPO	logFCTO
AT5G60408	MIR391	4.7	3.9	AT5G07335	U4.1*	−4.9	−4.1
AT5G36970	NHL25	3.3	3.2	AT3G20400	EMB2743	−4.3	−3.6
AT2G37720	TBL15	2.8	2.6	AT3G54340	AP3	−4.1	−4.3
AT1G56250	PP2‐B14	2.4	2.3	AT5G07345	U1a*	−4.1	−2.9
AT4G16820	PLA‐I{beta]2	2.2	1.7	AT3G48010	CNGC16	−3.6	−3.6
ArthCr091	ArthCr091	2.2	2.5	AT5G40210	UMAMIT42	−3.5	−4.1
AT1G56240	PP2‐B13	1.9	2.1	AT4G01970	STS	−3.4	−4.1
AT3G23250	MYB15	1.9	2.3	AT2G36750	UGT73C1	−3.0	−3.0
AT4G39340	EC1.4	1.8	1.8	AT3G28740	CYP81D11	−2.8	−3.0
AT5G10250	DOT3	1.7	1.0	AT1G02230	NAC004	−2.8	−3.4
AT5G47850	CCR4	1.6	2.6	AT1G74080	MYB122	−2.8	−2.9
AT5G64905	PROPEP3	1.6	2.0	AT2G29480	GSTU2	−2.7	−2.9
AT5G42380	CML37	1.5	2.0	AT1G61120	TPS04	−2.4	−2.5
AT1G01560	MPK11	1.4	2.0	AT2G36800	DOGT1	−2.3	−2.0
AT2G18660	PNP‐A	1.4	1.3	AT2G29490	GSTU1	−2.2	−2.3
AT5G01550	LECRKA4.2	1.3	1.3	AT5G65690	PCK2	−2.1	−1.9
AT4G25200	HSP23.6‐MITO	1.3	1.1	AT4G34135	UGT73B2	−2.0	−1.9
AT3G46230	HSP17.4	1.3	1.0	AT1G14080	FUT6	−1.9	−3.2
AT1G05610	APS2	1.3	1.2	AT1G05530	UGT75B2	−1.9	−2.5
AT5G66400	RAB18	1.2	1.0	AT2G39330	JAL23	−1.8	−1.7
AT1G11570	NTL	1.2	1.1	AT3G13170	ATSPO11‐1	−1.7	−1.9
AT5G03210	DIP2	1.2	1.3	AT5G59590	UGT76E2	−1.7	−2.0
AT2G40180	PP2C5	1.1	1.0	AT2G29420	GSTU7	−1.7	−1.9
AT4G04540	CRK39	1.1	1.6	AT3G47420	G3Pp1	−1.7	−1.3
AT2G44910	HB4	1.12	0.7	AT1G17170	GSTU24	−1.6	−1.8
AT2G47140	SDR5	1.0	1.3	AT3G11340	UGT76B1	−1.6	−1.0
				AT1G05560	UGT75B1	−1.6	−1.7
				AT3G14660	CYP72A13	−1.5	−1.8
				AT1G05680	UGT74E2	−1.5	−1.8
				AT2G37770	ChlAKR	−1.5	−1.7
				AT5G16970	AER	−1.5	−1.3
				AT4G22070	WRKY31	−1.4	−2.5
				AT1G12940	NRT2.5	−1.4	−1.9
				AT2G36380	ABCG34	−1.3	−1.1
				AT1G66600	ABO3	−1.2	−1.1
				AT1G64940	CYP89A6	−1.2	−1.2
				AT1G14860	NUDT18	−1.2	−0.8
				AT4G34131	UGT73B3	−1.2	−1.1
				AT1G55850	CSLE1	−1.1	−1.2
				AT1G02520	ABCB11	−1.1	−1.6
				AT1G76690	OPR2	−1.1	−1.2
				AT3G09270	GSTU8	−1.1	−1.2

The table shows the named loci that are differently expressed in progesterone‐ and testosterone‐treated *A. thaliana* shoots compared with mock‐treated controls (DMSO treatment). Here, we list *A. thaliana* loci, gene names and log fold changes. Log fold changes were determined by RNAseq experiments of 4 biological independent replicates. *P*‐values for all shown loci are below 0.05, indicating a statistically significant difference to mock‐treated control.

Investigations of these 68 gene loci in NCBI and additionally using the BAR Arabidopsis eFP Browser (https://bar.utoronto.ca/efp_arabidopsis/cgi‐bin/efpWeb.cgi?dataSource=Hormone [24 February 2024]) could ensure that both PO and TO act in a BR‐independent manner. This can be seen by the fact that the expression of important BR‐related transcription factors that are responsible for BR‐related growth, like BIL7 (Miyaji et al., 2024), is not changed. This is supported by the observation that the loss‐of‐function lines bri1 and bsk1, characterised by inoperable BR signalling, still show reduced root length in response to PO and TO (Figure S5).

Notably, the 3β‐HSD SDR5 (AT2G47140; Figure 3B) is upregulated in response to both progesterone and testosterone treatment.

## DISCUSSION

### Angiosperms have the enzymatic machinery to produce progesterone

In 1989, the first comprehensive study investigating the occurrence of mammalian‐like steroids (androgens, oestrogens and progestogens) in plants using radioimmunoassay was published. A total of 128 plants from more than 50 families were tested, providing a first impression of how abundant these steroids are in the plant kingdom (Simons & Grinwich, 1989). However, the results were critically evaluated due to problems with antibody specificity, which cast doubt not only on the abundance but also on the number of mammalian‐like steroids (Iino et al., 2007; Lindemann, 2015; Klein, 2024). With technical advances, it has become easier to detect progestogens and androgens (exemplarily: using HPLC‐MS/MS or GC–MS systems). Though, a multitude of investigations were conducted concerning progesterone, including in various plants (Iino et al., 2007; Simerský et al., 2009; Pauli et al., 2010). We have just recently published a study (Shiko et al., 2023) that focuses again on the whole concept of steroidogenesis in plants by examining 41 species across the green plant lineage. This study could show that angiosperms produce a broad range of progestogens such as PR, PO and 17α‐OHPR, as well as androgens such as DHEA, AD, TO and DHT. Furthermore, this study found that steroid profiles are species‐ and tissue‐dependent, as already discussed by Simons and Grinwich (1989).

Here, we show that the enzymatic machinery for PO biosynthesis is conserved in angiosperms, independent of the occurrence of cardenolides in the plant. In our analysis, only monocot species *Spirodela polyrhiza*, *Zingiber officinalis* and the eudicot species *B. officinalis* and *Ribes rubrum* did not contain a sterol side‐chain cleaving enzyme from the CYP87A family. As a result, *Spirodela polyrhiza* was one of only 2 species (both monocots) that did not contain progestogens or androgens in the study by Shiko et al. (2023), while *Z. officinalis*, *B. officinalis* and *R. rubrum* were not analysed within this study.

Therefore, we conclude that most angiosperms can form PO and that progestogens and androgens are widespread in angiosperms.

### Progestogens and androgens can modulate plant growth and development

Given that the occurrence of progestogens and androgens is conserved in plants and the enzymatic machinery behind them is also conserved, we wondered if these molecules have a conserved physiological function in plants. We showed that all tested progestogens and androgens (in concentrations of 0.03 mM) led to a dramatically reduced root length of *A. thaliana* seedlings (Figure 4) compared with pure medium and mock treatment (DMSO).

To analyse whether this effect is conserved in angiosperms, we sowed another 42 plant species (19 *Brassicacea* Table 1, Figure S2; 23 non‐Brassicales angiosperms, Table 2, Figure S3) in the presence of PO and TO. 60% of both the *Brassicaceae* species (including *A. thaliana* and *Brassica oleracea* convar. *capitata* var. *rubra*) and the non‐Brassicales angiosperms (including *Plantago major* collected in Dittelbrunn, Schweinfurt, Germany, and the medicinal plant species *Centaurium erythraea*) showed reduced root length. Interestingly, the cardenolide‐containing Brassicaceae species (*Erysimum crepidifolium* and *Erysimum cheiri*), which can use PO as a substrate for cardenolide formation, show a stronger reaction to PO and TO compared with *Malcolmia maritima* (*Malcolmia* is the sister genus of *Erysimum* within the *Brassicaceae* family and should be used as a cardenolide‐free control when working with *Erysimum* species; Hendriks et al., 2023). The two *Plantaginaceae* (Lamiales) plants analysed (the cardenolide‐containing species *Digitalis purpurea* and the cardenolide‐free species *Plantago major*) showed a contrary effect. The cardenolide‐free species *Plantago major* reacted sensitively to both PO and TO, while *Digitalis purpurea* showed no altered root morphology in the presence of these two steroids.

In addition to altered root length, uncoordinated cell growth and increased lateral root formation were also observed in the roots of many plant species treated with PO and TO (Figures S2 and S3). Even some plants that did not show altered root length exhibited disrupted cell organisation (e.g. the PO‐treated Do‐not‐forget‐me *Myosotis sylvatica*).

We conclude that progestogens and androgens influence root development in many plant species, but the effects are species‐dependent. This is consistent with the species‐dependent progestogen and androgen profiles of angiosperms (Simons & Grinwich, 1989; Shiko et al., 2023). Regardless of the question of progestogens and androgens acting as signalling molecules in plants, this seems remarkable. Artificial steroid pollution can be detected on all continents inhabited by humans (see, for example, the following studies; Africa: Damkjaer et al., 2018; Asia: Yao et al., 2018; Europe: Jauković et al., 2022; North America: Goeury et al., 2022; South America: González et al., 2020). In our experiments, we used concentrations of 10–60 μM of the used steroids. This is congruent to 3–9 mg L^−1^. Fortunately, steroid concentrations as high as this are, to our knowledge, not described in nature. The highest concentration of a single progestogen or androgen was found in 1.9 μg L^−1^ androsterone (reviewed in Almazrouei et al., 2023). Nevertheless, the concentrations had a dramatic impact on root length and morphology. Therefore, it is possible that lower concentrations, especially in combinations, will have a negative impact on root development, too. Exemplarily, in Mafisa, Morocco, wastewater contains a total of 2.0 ng L^−1^ of steroids in the dry season (Damkjaer et al., 2018). Even a small disturbance in root formation can have strong impacts on agricultural yields and seed‐plant‐based ecosystems (Calleja‐Cabrera et al., 2020; Lynch, 2019; Lynch, 2022). If steroids in concentrations found in wastewater can disturb root development, especially in stressful environmental conditions (exemplarily heat and drought stress; Calleja‐Cabrera et al., 2020), they have to be analysed in the following studies. Nevertheless, together with increased temperatures and reduced and prolonged drought periods in many areas of this planet, steroid pollution and its potential effects on root growth may be an underestimated threat to stable agricultural yields. Therefore, the physiological effects of progestogens and androgens on plants deserve more attention. Today, comparatively little is known, and most results are obtained by exogenous application with gradual recording of physiological properties. Iino et al. (2007) reported a growth‐promoting effect of progesterone on *A. thaliana* seedlings at low concentrations (0.01–1 μM), but at higher concentrations, its effect was reversed. Concentration‐dependent root and shoot growth‐promoting effects at very low concentrations (below 0.001 mM) of progesterone have also been demonstrated in chickpea (Erdal & Dumlupinar, 2011), and sunflower (Bhattacharya & Gupta, 1981). In winter wheat and *A. thaliana*, the application of AD and PO at similarly low concentrations also had an effect on promoting flower development (Janeczko & Filek, 2002). Progestogens and androgens might also be involved in the response to biotic stresses, as PO treatment has been shown to alleviate necrotic symptoms caused by *Pseudomonas syringae* and *Pseudomonas fluorescens* in *A. thaliana* (Janeczko et al., 2013). In addition, DHEA accumulates in *A. thaliana* tissues infected with *Alternaria brassicicola* (Oktay et al., 2025). Overall, further studies are needed to understand in detail the function of progestogens and androgens in plants and to estimate the consequences of steroid pollution on agriculture and natural ecosystems.

### Progesterone and testosterone influence root morphology in *A. thaliana* in a brassinosteroid‐independent manner

Progestogens, androgens and BRs share the same backbone structure (sterane, see Figure 1). That is why some enzymes involved in BR biosynthesis can also convert progestogens and androgens (see exemplarily: Klein et al., 2025). This is especially true for enzymes that occur in both plants and mammals. The steroid 5α‐reductase DET2 in plants is a homologue of the mammal steroid 5α‐reductase. Therefore, it is not surprising that human SRD5A1 or SRD5A2 expressed in DET2 knock‐out lines cure the dwarf phenotype of DET2 knock‐out (Li et al., 1997). This is why the scientific community has debated whether PO or TO treatment could cause competitive inhibition of DET2 (Li et al., 2022).

Competitive inhibition of DET2 could explain the reduced root length of plants treated with PO and TO. To challenge this hypothesis, we generated DET2 overexpression lines under the control of a strong CamV 35S promoter. The resulting *A. thaliana* plants had significantly longer roots than wild‐type plants (Figure 7). Nevertheless, DET2 overexpression lines did not show any reduced effects after PO and TO treatment compared with controls (Figure 7). Furthermore, PO and TO treatments did not alter either the wild‐type BR profiles (Figure 8) or the BR profiles of *A. thaliana* lines overexpressing DET2 (Table S4). We conclude that exogenous PO and TO treatments do not inhibit DET2 or any other BR biosynthetic enzyme.

Based on the structural similarity of progestogens and androgens to BRs, PO and TO could bind to the brassinosteroid receptor BRI1. This could lead to increased BR signalling (see Figure S5). To challenge this hypothesis, we treated *A. thaliana* plants for 1 h with PO and TO and used the plant material for RNAseq analyses. We observed neither increased nor decreased expression of BR‐regulated genes, which are also related to the biosynthesis and signalling (Figure 9). This is supported by our observation that even a BRI1 loss‐of‐function line, and a knock‐out line of the BR signalling factor BSK1 were progesterone‐ and testosterone‐sensitive (Figure S5).

We conclude that progestogens and androgens act in a BR‐independent way. In contrast to BRs, little is known about the specific physiological functions of mammalian‐like steroids in plants. We showed here that the three enzymes for PO biosynthesis are conserved in angiosperms. It was shown previously that the conversion of progestogens into androgens follows a conserved pathway in plants (Shiko et al., 2023). The described enzymes for PO biosynthesis are, following the Arabidopsis eFP Browser (https://bar.utoronto.ca/efp/cgi‐bin/efpWeb.cgi [25 February 2025]), expressed with enhanced intensity in *Arabidopsis thaliana* roots. This fits the 8 times higher progestogen and androgen levels in *Arabidopsis* roots compared with *Arabidopsis* shoots. We conclude an organ‐specific biosynthesis of progestogens and androgens. Nevertheless, the treatment of *Arabidopsis thaliana* roots with progesterone or testosterone increased steroid levels in shoot material, indicating steroid transport from roots to shoots (Table S8). Plants lack close homologues of animal nuclear steroid receptors, as Bishop and Koncz (2002) stated after a complete search of the *Arabidopsis* genome for related genes (Arabidopsis Genome Initiative 2000). The most considerable aspect was the identification of *Arabidopsis* MSBP1 and its two homologues MAPR2, MAPR3 and MAPR4, which can bind PO, 5α‐DHT, 24‐epibrassinolide and probably also other steroids (Yang et al. 2005, Iino et al., 2007 reviewed by Li et al., 2022). MSBP1 interacts with BAK1 and thereby reduces the BR response (Song et al., 2009). In addition, MSBP1 contributes to root growth by stimulating PIN‐FORMED2 (PIN2) auxin efflux carrier redistribution and therefore provides root gravitropism (Yang et al., 2008). This could explain the effects of exogenous PO and TO treatment on *A. thaliana* roots. Within this study, we treated angiosperm plants with high amounts of progestogens and androgens to analyse the effects of a strongly changed steroid metabolism. Therefore, it must be discussed that physical, physiological and biochemical changes within the roots can cause the described phenotypes. Exemplarily, the amounts of free phytosterols are strictly controlled because of their high influence on membrane fluidity and integrity (Ferrer et al., 2017; Rogowska & Szakiel, 2020; Schaller, 2004). Structurally, comparable progestogens and androgens could induce a changed membrane fluidity or integrity, as described exemplarily for DHEA (Oktay et al., 2025) by direct interaction with cell membranes, or because they are conjugated by the same enzymes that conjugate phytosterols (exemplarily sterol acyltransferases, sterol glycosyl transferases and steryl glycoside acyltransferases; Ferrer et al., 2017; Klein et al., 2025). Nevertheless, it can be asked if a change in the basal physiology of living organisms, such as their membrane properties, can explain the strong differences in PO and TO sensitivity even in angiosperms and even one plant family (*Brassicaceae*). Alternatively, exogenously applied progestogens and androgens could result in feedback regulation of progestogen formation, or even phytosterol biosynthesis. A feedback regulation of progesterone biosynthesis was discussed previously (Klein, Horn, et al., 2021). When in *Digitalis lanata* the conversion of PO by PRISEs was inhibited by RNAi‐mediated PRISE‐knockdown, the resulting plants showed lower PO values. This was seen as a hint for a feedback regulation mechanism (Klein, Horn, et al., 2021). The phytosterol biosynthesis pathway is controlled by feedback regulation, too (exemplarily: Zhang et al., 2020). Keeping in mind that phytosterols, progestogens and androgens are similar in their structure, it can be discussed if an exogenous treatment with high amounts of progestogens and androgens can induce feedback regulation of phytosterol biosynthesis. Moreover, the local biosynthesis and a very strict control of brassinosteroids are necessary for optimal root growth (Retzer et al., 2019; Korbei & Luschnig, 2021; Vukašinović et al., 2021). This fine local distribution could be torpedoed by a treatment with high amounts of progestogens and androgens, exemplarily by feedback regulation of phytosterol biosynthesis. Nevertheless, it remains very unclear if this hypothesis could explain the strong differences in PO and TO sensitivity.

However, more studies are needed to investigate the signalling pathways of PO and TO and the molecular mechanisms of their effect on root growth. If a specific receptor for these types of steroids can be identified, resulting in the discovery of a specific signalling pathway, progestogens and androgens could be seen as additional signalling factors.

## CONCLUSION

- The enzymatic machinery for progesterone biosynthesis is conserved in angiosperms, and progestogens and androgens occur in most angiosperms.
- Progesterone and testosterone influence root morphology in many angiosperms, but the effects are species‐dependent.
- Progesterone and testosterone treatment do not inhibit BR biosynthesis.
- Progesterone and testosterone treatment do not affect BR‐dependent genes.
- We conclude that progesterone and testosterone act in a BR‐independent manner. If these molecules are signalling factors or influence growth and development in alternative ways, this must be clarified in follow‐up studies.

## MATERIAL AND METHODS

### Plant material and culture

Within this study, seeds of the following producers and distributors were used:

Schloss Gut Obbach (Gut Obbach Schäfer GbR, Obbach, Euerbach, Bavaria, Germany): *Secale cereal* L. cv. Dukato.

Philipp Klein GmbH (Miltenberg, Germany): *Lepidium sativum* L.

Magic Garden Seeds GmbH (Regensburg, Germany): *Barbarea vulgaris* W.T.Aiton, *Brassica oleracea* convar. *capitata* var. *rubra* L. cv. Schwarzkopf, *Digitalis purpurea* L., *Diplotaxis tenuifolia* (L.) DC., *Eruca vesicaria* subsp. *sativa* (L.) CAV., *Erysimum cheiri* (L.) CRANTZ, *Hesperis matronalis* L., *Isatis tinctoria* L., *Matthiola incana* (L.) W.T.AITON, *Nasturtium officinale* W.T.AITON, *Petroselinum crispum* (MILL.) FUSS cv. Mooskrause, *Oenothera speciosa* NUTT., *Centaurium erythraea* RAFN., *Ruta graveolens* L.

Quedlinburger Saatgut GmbH (Aschersleben, Germany): *Beta vulgaris* subsp. *vulgaris* CONDITIVA group L., *Camelina sativa* (L.) CRANTZ, *Cucumis sativus* L. cv. Vorgebirgstraube, *Fragaria vesca* L. cv. Rügen, *Pelargonium zonale* (L.) L'HÉR., *Sinapsis alba* L., *Solanum lycopersicum* L. cv. Harzfeuer.

Botanical garden (Friedrich Schiller University, Jena, Germany): *Erysimum crepidifolium* RCHB. and *Nymphaea colorata* PETER.

Templiner Kräutergarten (Templin, Germany): *Aurinia saxatilis* (L.) DESV., *Cochlearia officinalis* L., *Hibiscus sabdariffa* L., *Linum usitatissimum* L., *Malcolmia maritima* (L.) W.T. Aiton, *Sisymbrium officinale* (L.) SCOP., *Vaccinium myrtillus* L., *Valerianella carinata* LOISEL.

Dürr Samen Stephan Schwenk e.K. (Reutlingen, Germany): *Sinapsis alba* L.

Carl Pabst, Samen & Saaten GmbH (Großbeeren, Germany): *Raphanus sativus* var. *sativus* L. cv. Riesenbutter.

Sonnentor Kräuterhandelsgesellschaft mbH (Großgöttfritz, Austria): *Trigonella foenum‐graecum* L.

Bruno Nebelung GmbH (Everswinkel, Germany): *Allium schoenoprasum* L. cv. Nelly, *Lobularia maritima* (L.) DESV. cv. Schneeteppich, *Myosotis sylvatica* EHRH. EX HOFFM. cv. Heavenly Blue.

SPERLI GmbH (Everswinkel, Germany): *Papaver rhoeas* L.

*Plantago major* L. and *Taraxacum officinale* L. seeds were collected from wild populations in Dittelbrunn (Schweinfurt, Bavaria, Germany).

Turions from the *Spirodela polyrhiza* (L.) SCHLEID. clone 9256 were available in the stock collection of the Friedrich Schiller University Jena. They were cultivated as described in Appenroth and Nickel (2010). Turions are dormant, bud‐like structures produced by over 15 families of aquatic fresh water plants as a stress response and overwintering survival strategy (Appenroth & Nickel, 2010; Pasaribu et al., 2023). It has been shown previously that the germination of turions of *Spirodela polyrhiza* and of zygotic seeds share many signalling pathways and genes (Pasaribu et al., 2023). That is why we included turion germination within this study.

Moreover, seeds of *Arabidopsis thaliana* Col. 0 (*A. thaliana*), as well as the BRI1 loss‐of‐function mutant bri1‐4 (*bri*; NASC ID: N3953), and the BSK1 loss‐of‐function line *bsk1* (NASC ID: N806767) were available in the laboratories of the Friedrich Schiller University (Jena, Germany).

All plant seeds were surface sterilised by the following protocol:

Incubation of the seeds in 1 mL 2‐propanol for 30 s, washing the seeds in autoclaved ultrapure water, incubation for 8 min in sterilisation solution (sterilisation solution: 5% chlorine bleach and 20% N‐lauroylsarcosine sodium salt, Merck, Germany) sowing seeds on MS medium (Murashige and Skoog, 1962) containing 4.4 g L^−1^ MS salts (Duchefa Biochemie, Haarlem, Netherlands), 0.5 g L^−1^ MES buffer (Carl Roth GmbH + Co. KG, Karlsruhe, Germany), 13.7 g L^−1^ sucrose (Carl Roth GmbH + Co. KG, Karlsruhe, Germany), 3 g L^−1^ gelrite (Duchefa Biochemie, Haarlem, Netherlands). The pH was adjusted to 5.7–5.8 before autoclaving the MS medium.

After 2 days of stratification (4°C), plants were cultivated in the following conditions: long day (16 h of light and 8 h of dark) with light intensity of 60–70 μM m^−2^ s^−1^ at 22°C.

The protocol was slightly adapted for the following species: For *Myosotis*, *Petroselinum* and *Taraxacum*, 2 weeks of dark culture were conducted after stratification. For *Cucumis*, *Hibiscus*, *Pelargonium* and *Solanum*, no stratification was conducted. *Secale* and *Trigonella* were sown on watered (autoclaved tap water) cotton tissues, and no stratification was conducted. *Nymphaea* was cultivated in sugar‐free, liquid MS medium, and no stratification was conducted.

Turions of *Spirodela polyrhiza* were cultivated in sugar‐free nutrient medium for *Lemnaceae* according to Appenroth (Appenroth et al., 1996; Appenroth et al., 2018).

### Steroid treatment

For steroid treatment, 60 μL of a sterile‐filtered 25 mM steroid stock solution (solved in DMSO) was prepared from pregnenolone (PR), progesterone (PO), 5α‐pregnane 3,20‐dione (5α‐dihydroprogesterone, 5α‐DHP), dehydroepiandrosterone (DHEA), androstenedione (AD), testosterone (TO) and 5α‐dihydrotestosterone (5α‐DHT), and estradiol (ER). 60 μL stock solutions were added to 50 mL MS medium or tap water (after autoclaving), resulting in a 30 μM final concentration. Pure 60 μL DMSO was added to 50 mL MS medium/tap water as a mock treatment.

### Sterol treatment

To analyse the effects of the steroid precursor cholesterol (CO) on root morphology, we prepared 25 mM stock solutions of CO (dissolved in ethanol). We prepared final concentrations of 60 μM by adding 120 μL of stock solution to 50 mL of MS medium. To prepare a final concentration of 30 μM, 60 μL of stock solution and 60 μL of pure ethanol were added to the medium. For mock treatment, 120 μL of pure ethanol was added to the medium.

### Determination of root morphology

The length of the longest root of 9‐day‐old, steroid‐treated *A. thaliana* was determined with a commercially available ruler. Since other plant species have deviating germination periods, this is different for the other observed plants. Pictures and data of the analysed seedlings are available in the Figures S1 and S2, while data and pictures of *A. thaliana* are given in the main text (Figures 4 and 5).

To observe the effect of steroids on the root morphology of germinated seedlings, 10‐day‐old *A. thaliana* seedlings germinated on pure MS medium were transferred to MS medium supplemented with progestogens or androgens (30 μM final concentration), while MS medium supplemented with DMSO was used as a mock treatment.

The entire roots were imaged using an AXIO Imager.M2 (Zeiss Microscopy GmbH, Jena, Germany) equipped with a 10× objective (N‐Achroplan 10×/0.3) or a 40× dipping objective (W N‐Acroplan 40×/0.75). Bright‐field images were recorded with a colour camera AXIOCAM 503 colour (Zeiss Microscopy GmbH). Digital images were processed with the ZEN software (Zeiss Microscopy GmbH), treated with Adobe^®^ PhotoShop to optimise brightness, contrast and colouring.

### Overexpression of AtStR1, AtStR2 and AtDET2 in *A. thaliana*

Constructs for the overexpression of AtStR1 (At4g24220) and AtStR2 (At5g58750) based on pEarleyGate 100 were designed previously (Klein, Ernst, et al., 2021).

The coding sequence of AtDET2 (At2g38050) was amplified by Phusion^®^ High Fidelity DNA Polymerase (New England Biolabs, Ipswich, MA, USA) with the following primers: JK_AtDet2_pFAU27_for: 5′AATT**TCTAGA** ATGGAAGAAATCGCCGATAAAACC3′. The restriction site of XbaI is given in bold letters; the part of the primer that complements the AtDET2 coding sequence is underlined.

JK_AtDet2_pFAU27_rev: 3′TATA**GTCGAC** TCAGTACACAAAAGGAATAACAGC5′. The restriction site of SalI is given in bold letters; the part of the primer that complements the AtDET2 coding sequence is underlined.

Both the PCR amplified AtDET2 coding sequence and vector pFAU27 (donated by Ruth Stadler, Department of Biology, Friedrich Alexander University Erlangen‐Nürnberg, Germany) were digested with XbaI‐HF and SalI‐HF (New England Biolabs, Ipswich, MA, USA) for 16 h at 37°C in rCutSmart™Buffer (New England Biolabs, Ipswich, MA, USA). Digested insert and vector backbone were purified using NucleoSpin^®^ Gel and PCR Clean‐up (Macherey‐Nagel GmbH & Co. KG, Düren, Germany). Insert and vector backbone were combined in a ratio of 4:1 and ligated at 4°C for 16 h using T4 Ligase (New England Biolabs, Ipswich, MA, USA). Ligation products were used for the transformation of NEB® 5‐alpha Competent *Escherichia coli* (New England Biolabs, Ipswich, MA, USA). Plasmids were isolated using GeneJET Plasmid‐Miniprep Kit (Thermo Fisher Scientific Inc., Waltham, MA, USA) and sequenced using the following primer: JK_pFAU27_NOS_seq: 5′TAATCATCGCAAGACCGGCA3′.

Plasmids were used for the transformation of *Agrobacterium tumefaciens* strain GV3101. *A. thaliana* Col‐0 was transformed using the floral‐dip method (Clough & Bent, 1998) as described previously (Klein, Ernst, et al., 2021; Shiko et al., 2023).

Transgenic plants were selected by sowing seeds of dipped plants on MS medium supplemented with 250 mg mL^−1^ cefotaxime and 50 mg mL^−1^ kanamycin. The surviving plants were tested for integrated T‐DNA by PCR against neomycin phosphotransferase II, the kanamycin resistance gene, using the following primers (described by Mußmann et al., 2011): nptII_for: 5′TGAATGAACTGCAGGACGAG3′; nptII_rev: 5′AATATCACGGGTAGCCAACG3′. Overexpression of AtStR1, AtStR2 and DET2 was ensured by quantitative PCR (qPCR) using the following primers:

JK_qAtStR1_for: 5′ATGAAACATCACCATCACCATCA3′;

JK_qAtStR1_rev: 5′GTCAACGTTGGTGAAAGGGC3′;

JK_qAtStR2_for: 5′CAATGGCTCGTGGAGAAGGT3′;

JK_qAtStR2_rev: 5′TTCCGGCCAAATCTCCTTCC3′.

JK_qDET2_for: 5′ACCGATTGCTTGGTTCGTCA3′.

JK_qDET2_rev: 5′CCAGCCTCCTCTCGGTATCA3′.

RPS18_for: 5′GTCTCCAATGCCCTTGACAT3′.

RPS18_rev: 5′TCTTTCCTCTGCGACCAGTT3′.

Primers of AtStR1 and AtStR2 were described previously in Klein, Ernst, et al. (2021) and Klein, Horn, et al. (2021).

Primers against RPS18 (successfully used in previous studies; exemplarily: Shiko et al., 2023) were used for normalisation.

Within this study, we show the data of 3 (DET2) or 4 (AtStRs) independent transgenic lines (generated from different floral‐dipped mother plants).

The results of plant transformation can be found in Figure S3.

### Isolation of genomic DNA and PCR

DNA of *A. thaliana* was isolated using a CTAB (Carl Roth GmbH + Co. KG, Karlsruhe, Germany) based method as described in Klein, Horn, et al. (2021), Shiko et al. (2023). PCRs were performed in a Gene Explorer Advanced (Hangzhou Bio‐Gener Technology Co., Ltd, China). Each reaction was performed in a 20 μL assay composed of the following: 2 μL DNA solution, 2 μL each primer, 4 μL H_2_O and 10 μL FastGene Taq 2× Ready Mix (NIPPON Genetics EUROPE GmbH, Düren, Germany). The PCR program was set as follows: a preincubation at 95°C for 180 s was followed by 40 cycles at 95°C for 30 s, 60°C for 30 s and 68°C for 60 s. PCR was finalised by a final amplification at 68°C for 60 s. For the PCR with Phusion^®^ High Fidelity DNA Polymerase (New England Biolabs, Ipswich, MA, USA), denaturation temperature was enhanced to 98°C and the elongation temperature was set at 72°C.

### RNA isolation and quantitative PCR

RNA of *A. thaliana* was isolated using TRIzol^®^ Reagent (Invitrogen™ by Thermo Fisher Scientific, USA), as described previously (Chomczynski, 1993). RNA was transcribed into cDNA using a RevertAid First Strand cDNA Synthesis Kit (ThermoFisher Scientific, Ipswich, MA, USA). Quantitative real‐time PCR (qPCR) was performed in a Bio‐Rad CFX96 Thermal Cycler (Bio‐Rad Laboratories GmbH, Germany) using 96‐well plates. For every sample, a 20 μL reaction mixture was used, which was composed of 1 μL cDNA, 8.4 μL H_2_O, 2 μL dNTPs (ThermoFisher Scientific, Ipswich, USA), 1 μL EvaGreen^®^ Plus Dye (Biotium, Inc., Fremont, CA, USA), 2 μL of each primer, 0.2 μL DreamTaq DNA Polymerase and 3.4 μL DreamTaq DNA Polymerase buffer (ThermoFisher Scientific, Ipswich, MA, USA). The qPCR reaction was performed as described above, including preincubation at 95°C for 180 s, followed by 40 cycles at 95°C for 10 s, 60°C for 50 s and 72°C for 60 s.

### RNAseq

7‐day‐old *A. thaliana* plants were transferred to PNM medium. After 2 days of adaptation to the new medium, plants were treated with 10 μM PO (dissolved in 0.12% DMSO) or 10 μM TO, while 0.12% DMSO was used as a mock treatment. Leaves were harvested after 1 h and immediately frozen in liquid nitrogen. This point in time was chosen because it was successfully used for DHEA previously (Oktay et al., 2025). Moreover, the changes in the transcriptome in response to brassinosteroids can be observed at early time points (3 h; https://bar.utoronto.ca/efp/cgi‐bin/efpWeb.cgi?dataSource=Hormone [12 May 2025]). Finally, this short time of treatment was chosen to avoid secondary changes in the transcriptome caused by a disturbed metabolism.

RNAseqs was conducted as described previously (Oktay et al., 2025). In brief: RNA was isolated as described above. RNAseq was conducted by GENEWIZ Germany GmbH (Azenta Life Science, Leipzig, Germany) and processed as described in the following:

RNA samples were quantified using Qubit 2.0 Fluorometer (Life Technologies, Carlsbad, CA, USA), and RNA integrity was checked using Agilent Fragment Analyser (Agilent Technologies, Palo Alto, CA, USA). RNA sequencing library preparation was prepared using NEBNext Ultra II RNA Library Prep Kit for Illumina following the manufacturer's instructions (NEB, Ipswich, MA, USA). Briefly, mRNAs were first enriched with Oligo(dT) beads. Enriched mRNAs were fragmented. First strand and second strand cDNA were subsequently synthesised. The second strand of cDNA was marked by incorporating dUTP during the synthesis. cDNA fragments were adenylated at 3' ends, and indexed adapter was ligated to cDNA fragments. Limited cycle PCR was used for library amplification. The dUTP incorporated into the cDNA of the second strand enabled its specific degradation to maintain strand specificity. Sequencing libraries were validated using NGS Kit on the Agilent Fragment Analyser (Agilent Technologies, Palo Alto, CA, USA) and quantified by using the Qubit 4.0 Fluorometer (Invitrogen, Carlsbad, CA, USA). The sequencing libraries were multiplexed and loaded on the flow cell on the Illumina NovaSeq 6000 instrument according to the manufacturer's instructions. The samples were sequenced using a 2 × 150 Pair‐End (PE) configuration v1.5. Image analysis and base calling were conducted by the NovaSeq Control Software v1.7 on the NovaSeq instrument. Raw sequence data (.bcl files) generated from Illumina NovaSeq were converted into fastq files and de‐multiplexed using Illumina bcl2fastq program version 2.20. One mismatch was allowed for index sequence identification. Raw data are available on NCBI (Geo accession ID: GSE286512).

Raw sequencing reads were assessed for quality using FastQC (version 0.11.9; https://www.bioinformatics.babraham.ac.uk/projects/fastqc). Adaptor trimming, quality filtering and read preprocessing were performed using fastp (version 0.23.2; Chen et al., 2018). In detail, 5′ and 3′ bases with a Phred quality score below 28 were cut, and reads were removed if they had more than one ambiguous base, an average quality score below 28 or a length of fewer than 15 bases. Processed reads were aligned to the current *A. thaliana* genome (tair10.1) using Hisat2 (version 2.2.1) with standard parameters (Kim et al., 2019). The aligned reads were sorted and indexed using SAMtools (version 1.11; Li et al., 2009). Read counting was performed using featureCounts (version 2.0.1; Liao et al., 2014) with the tair10.1 annotation as reference. Differential gene expression analysis was performed using DESeq2 (version 1.38.3; Love et al., 2014), and comparisons having a false discovery rate (FDR; Benjamini & Hochberg, 1995) adjusted *P*‐value <0.05 were deemed to be statistically significant.

Genes were categorised according to their function found on The Arabidopsis Information Resource (TAIR) database (February 2024; Berardini et al., 2004). Keywords for each gene with locus identifier numbers were selected from GO‐term classifications using an original code utilising Python and pandas library (McKinney, 2010). The code provided matches for the locus number with functions (GO terms) recorded on TAIR, the number of genes that a function has matched with, as well as a bubble plot with desired variables from the RNAseq results.

RT‐qPCR was not performed to validate the expression data as this study includes robust statistical analysis with the RNAseq data (Coenye, 2021).

### Progesterone and testosterone conversion assay

10‐day‐old *A. thaliana* plants were transferred to MS medium containing 30 μM progesterone or testosterone. Root and shoot material were harvested separately after 8 days of treatment, and the medium contaminations were carefully removed. In the following, the plant material was frozen in liquid nitrogen and lyophilised for 24 h. 20 mg lyophilised plant material were used for steroid extraction. Steroid extraction was performed using 20 mg lyophilised plant material as described in Shiko et al. (2023). In brief, flash‐frozen plant material was lyophilised. The dried material was pulverised, and 20 mg aliquots of each sample were extracted with 1 mL of extraction mixture (80% MeOH in water containing 100 ng mL–^1^ D9‐progesterone as internal standard, Merck, Germany). Samples were directly vortexed for 60 s and sonicated (Sonorex Super RK 100 H, BANDELIN electronic, Germany) for 5 min at 22° C. The crude extracts were centrifuged (HERMLE ZK233‐M2, HERMLE Labortechnik GmbH, Germany) for 60 s at 16000 ***g***, and the supernatant was used for the steroid analysis. It was performed by LC–MS/MS on an Agilent 1260 series HPLC system (Agilent Technologies) with a tandem mass spectrometer QTRAP 6500 (SCIEX, Darmstadt, Germany). A detailed description of the method is presented in the supplement (Table S1). This method was used to analyse the amounts of 5α‐DHP in progesterone‐treated roots or 5α‐DHT in testosterone‐treated roots.

### Brassinosteroid quantification

Extraction and quantification of endogenous BRs were performed as described in Oklešťková et al. (2017). Briefly, 3 replicates of approximately 3 mg (DW) of plant sample were extracted in ice‐cold 60% acetonitrile, and 25 pmol of deuterium‐labelled internal standards of BRs were added to each sample (Olchemim s.r.o. Olomouc, Czech Republic). After 12 h, samples were centrifuged and the supernatants were purified using 50 mg Discovery™ DPA‐6S cartridges (Supelco^®^, Bellefonte, PA, USA). After evaporation to dryness, samples were reconstituted in 40 μL methanol and analysed by liquid chromatography coupled to tandem mass spectrometry (UHPLC–MS/MS) (ACQUITY UPLC^®^ I‐Class System, Waters, Milford, MA, USA) using the Xevo™ TQ‐S MS triple quadrupole mass spectrometer (Waters MS Technologies, Manchester, UK). Detailed parameters of UHPLC–MS/MS analysis are described in Oklešťková et al. (2017) and Tarkowská et al. (2016).

### Evolutionary history

Sequence data were searched by blastp using the following databases: NCBI (https://blast.ncbi.nlm.nih.gov/Blast.cgi [24 February 2024]), Phytozome v13 (https://phytozome‐next.jgi.doe.gov/ [24 February 2024]; Goodstein et al., 2012), EnsemblPlants (https://plants.ensembl.org/index.html [24 February 2024]) and medicinal plant genome database (https://mpgr.uga.edu/ [24 February 2024]). Additionally, transcriptome data from the following NCBI bioprojects were analysed for species without available genome sequences: *Allium schoenoprasum* (PRJEB55612 and PRJNA847160), *Borago officinalis* (PRJEB50033), *Myosotis sylvatica* (PRJNA451174), *Pelargonium zonale* (PRJNA807121), *Plantago lanceolata* (PRJNA636383) and *Ribes rubrum* (PRJNA1131118). Detailed read quality control was performed as described above (RNAseq) and assembled using Trinity (version 2.15.2; PMID: 21572440) with default parameters. Sequences of interest were identified using tblastp (version 2.16.0; Altschul et al., 1990) in the resulting assemblies. These sequences can be found in Table S2. Evolutionary analyses were conducted in MEGA11 (Tamura et al., 2021). The evolutionary histories were inferred by using the maximum likelihood method and JTT matrix‐based model (Jones et al., 1992). The bootstrap consensus trees inferred from 1000 replicates (Felsenstein, 1985) are taken to represent the evolutionary history of the taxa analysed. Branches corresponding to partitions reproduced in less than 50% bootstrap replicates are collapsed. Initial tree(s) for the heuristic search were obtained automatically by applying Neighbour‐Join and BioNJ algorithms to a matrix of pairwise distances estimated using the JTT model and then selecting the topology with superior log likelihood value.

### Modelling of progesterone docking onto steroid 5α‐reductases

Amino acid sequences of steroid 5α‐reductases from angiosperms and mammals were obtained from NCBI, Phytozome13 and EnsemblePlants. 3D models of the respective enzymes were created using SWISS‐MODEL (https://swissmodel.expasy.org/interactive/; Bienert et al., 2017; Waterhouse et al., 2018) using the following templates recommended by SWISS‐MODEL. The Global Model Quality Estimation (GMQE) values for the homology models were all above 0.85, suggesting that the models are reliable (Waterhouse et al., 2018). We give here sequence identifiers from SWISS‐MODEL: *Nymphaea colorata* PETER. DET2 enzyme already existed within the template database (A0A5K1ELJ6.1.A); *Setaria italica* (L.) P. Beauv. DET2 template (A0A368RD67) was used for modelling the DET2 enzyme of *S. cereale* L.; *Striga asiatica* (L.) KUNTZE. DET2 template (A0A5A7RF69) was used for modelling DET2 enzyme of *P. lanceolata* L. *Ornithorhynchus anatinus* SHAW. DET2 enzyme already existed within the template database (A0A6I8NGS6); *Phascolarctos cinereus* GOLDFUSS. DET2 template (A0A6P5LUY6) was used for modelling the DET2 enzyme of *Vombatus ursinus* SHAW.; the *Bos taurus* LINNAEUS. DET2 enzyme template (A5PJS2) was used for modelling the DET2 enzyme of *Cervus elaphus* LINNAEUS. Possible positions of progesterone and NADP^+^ inside the binding pockets were calculated using AutoDock Vina (AutoDock Vina v1.2.5; Trott and Olson, 2010; Eberhardt et al., 2021). The centre of the binding pocket was defined as a three‐dimensional grid with coordinates, *X* = −5, *Y* = −35, *Z* = 20, and dimensions of 16 Å × 20 Å. The energy range of 10 to 50 and the exhaustiveness value of 50 were set as default settings for the docking experiment parameters. The visual evaluation and documentation were performed using ChimeraX (Chimera v1.17.1; https://www.rbvi.ucsf.edu/chimera; Pettersen et al., 2004).

### Statistical analysis

Analyses for root growth data were performed using Rstudio (R. v4.4.0) enhanced with various packages (Table S3). Data were checked for variance homogeneity with Levene's test and for normal distribution within the data frame. Since both statistical properties were denied, an aligned rank transform (ART) ANOVA test was performed to evaluate significant differences between the groups. This test combines the properties of nonparametric tests (e.g. Kruskal–Wallis; Mann–Whitney U) by ranking the data with the possibility of investigating various factors simultaneously, as done by ANOVA (Wobbrock et al., 2011). For visualisation, a post‐hoc test was performed implementing Bonferroni correction, and results were displayed in a boxplot. Graphs were determined with GraphPad Prism version 8.

## Author Contributions

All authors contributed to the study. **JK** designed the study. Material preparation, data collection and analysis were performed mainly by **KLK** and **SP** with help from **JO**, **FF**, **CO**, **M** **D**, **JM**, **ACUF** and **JK**. **EB** was responsible for the bioinformatics analysis of RNAseq experiments, while **HO** designed the scientific drawings and the graphical abstract. **KLK** and **JK** wrote the first draft of the manuscript with the help of **JM**, **MS** and **ACUF**. Further, all authors commented on previous versions of the manuscript. All authors read and approved the final manuscript.

## Conflict of Interest

The authors declare no conflict of interest.

## Supporting information


**Figure S1.** Dose‐dependent effects of steroids on root length of *Arabidopsis thaliana*. We here show the effects of pregnenolone (PR), progesterone (PO), 5α‐dihydroprogesterone (5α‐DHP), DHEA, androstenedione (AD), testosterone (TO), 5α‐dihydrotestosterone (5α‐DHT) and oestradiol (ER) on the root length of *A. thaliana*. *A. thaliana* wild‐type seeds were germinated on MS medium supplemented with these steroids in concentrations between 10 and 30 μM (only for 5α‐DHP, an additional concentration of 60 μM was used). Root length was determined after 9 days. The figure shows the root length in cm (mean ± SEM). Statistical differences, indicated by asterisks (*P ≤ 0.05; **P ≤ 0.01; ***P ≤ 0.001), were determined by one‐way ANOVA and Turkey test.

**Figure S2.** Seedling and root morphology of progesterone‐ or testosterone‐treated *Brassicaceae* species. The figure depicts the morphology of seedlings and roots of the following *Brassicaceae* species: (A) *Aurinia saxatilis* (L.) DESV. (B) *Barbarea vulgaris* W. T. Aiton, (C) *Brassica oleracea* convar. *capitata* var. rubra L. cv. Schwarzkopf, (D) *Camelina sativa* (L.) CRANTZ (E) *Cochlearia officinalis* L., (F) *Diplotaxis tenuifolia* (L.) DC., (G) *Eruca vesicaria* subsp. *sativa* (L.) CAV., (H) *Erysimum cheiri* (L.) CRANTZ, (I) *Erysimum crepidifolium* RCHB., (J) *Hesperis matronalis* L., (K) *Isatis tinctoria* L., (L) *Lepidium sativum* L., (M) *Lobularia maritima* (L.) DESV. cv. Schneeteppich, (N) *Malcolmia maritima* (L.) W. T. Aiton, (O) *Matthiola incana* (L.) W.T. Aiton, (P) *Nasturtium officinale* W. T. Aiton, (Q) *Raphanus sativus* var. *sativus* L. cv. Riesenbutter, (R) *Sinapsis alba* L., (S) *Sisymbrium officinale* (L.) SCOP. (a) gives the root lengths of the analysed plant as mean ± SEM. Statistical differences, indicated by asterisks (*P ≤ 0.05; **P ≤ 0.01; ***P ≤ 0.001), were determined by one‐way ANOVA and Tukey test. (b–e) are pictures of the morphology of the seedlings. (f–h) show microscopic pictures of the root tips of the analysed plant. b and f = MS control; c and g = DMSO mock treatment; d and h = 30 μM progesterone; e and h = 30 μM testosterone. Green arrows indicate uncoordinated cell growth, while white arrows indicate enhanced root hair development.

**Figure S3.** Seedling and root morphology of progesterone‐ or testosterone‐treated non‐Brassicales angiosperms. The figure depicts the morphology of seedlings and roots of the following *Brassicacea* species: (A) *Nymphaea colorata* PETER., (B) Spirodela polyrhiza (L.) SCHLEID., (C) *Allium schoenoprasum* L. cv. Nelly, (D) *Secale cereal* L. cv. Dukato, (E) *Petroselinum crispum* (MILL.) FUSS cv. Mooskrause, (F) *Taraxacum officinale* L.—wild population Dittelbrunn, (G) *Myosotis sylvatica* EHRH. EX HOFFM. cv. Heavenly Blue, (H) *Beta vulgaris* subsp. *vulgaris* CONDITIVA group L., (I) *Cucumis sativus* L. cv. Vorgebirgstraube, (J) *Valerianella carinata* LOISEL., (K) *Vaccinium myrtillus* L., (L) *Trigonella foenum‐graecum* L., (M) *Centaurium erythraea* RAFN., (N) *Pelargonium zonale* (L.) L'Hér., (O) *Digitalis purpurea* L., (P) *Plantago major* L. – wild population Dittelbrunn, (Q) *Linum usitatissimum* L., (R) *Hibiscus sabdariffa* L., (S) *Oenothera speciosa* NUTT., (T) *Papaver rhoeas* L., (U) *Fragaria vesca* L. cv. Rügen, (V) *Ruta graveolens* L. (W) *Solanum lycopersicum* L. cv. Harzfeuer. (a) gives the root lengths of the analysed plant as mean ± SEM. Statistical differences, indicated by asterisks (*P ≤ 0.05; **P ≤ 0.01; ***P ≤ 0.001), were determined by one‐way ANOVA and Turkey test. (b–e) are pictures of the morphology of the seedlings. (f–h) show microscopic pictures of the root tips of the analysed plant. b and f = MS control; c and g = DMSO mock treatment; d and h = 30 μM progesterone; e and h = 30 μM testosterone. Green arrows indicate uncoordinated cell growth, while white arrows indicate enhanced root hair development.

**Figure S4.** Cloning and overexpression of AtDET2 in *A. thaliana*. (A) We here show the used cloning strategy to obtain a construct for AtDET2 overexpression. The original insert of pFAU27 was removed by digestion with XbaI and SalI (highlighted in green). These restriction enzymes do not cut the coding sequence of AtDET2. AtDET2 amplificates with XbaI and SalI restriction sites were designed by PCR and inserted into the digested pFAU27. (B) Seeds of floral dip *A. thaliana* plants were selected using kanamycin. Plants surviving on kanamycin were analysed by PCR against nptII (the T‐DNA located resistance genes). We here show the PCR against nptII for several transgenic plants. (C) The intensity of DET2 expression in T‐DNA containing *A. thaliana* plants was tested by qPCR. As expected, DET2 expression intensity shows a strong variety between the transgenic plants. Lines 1 and 3 show a tremendous upregulation (factor 200 compared with the wild type), while it is still very strong for line 2 (factor 11). That is why we used these lines for following experiments.

**Figure S5.** The effects of progesterone and testosterone on loss‐of‐function mutants in brassinosteroid signalling. (A) We here show a simplified graphic of brassinosteroid signalling following Kim and Russinova (2020). The brassinosteroid receptor Brassinosteroid‐Insensitive1 (BRI1) remains, in the absence of brassinosteroids, inactive due to the autoinhibitory C‐terminus and its association with BRI1 Kinase Inhibitor1 (BKI1). As a consequence, Brassinosteroid‐Insensitive2 (BIN2) is constitutively active and phosphorylates the transcription factors BRI1‐EMS Suppressor1 (BES1)/Brassinazole‐Resistant1 (BZR1). This phosphorylation promotes their 14‐3‐3‐mediated degradation within the cytosol and thereby inhibits their DNA‐binding activities. In the presence of brassinosteroids, the receptor kinases BRI1 and BAK1 are activated. This activation leads to a dissociation of BKI1 from BRI1, as well as the phosphorylation and activation of BR‐Signalling Kinases (BSKs)/Constitutive Differential Growth (CDGs) and BRI1 Suppressor1 (BSU1). After activation, BSU1 dephosphorylates and inactivates BIN2, which will be degraded. These events result in the accumulation of Protein Phosphatase 2A (PP2A) within the nucleus. PP2A dephosphorylates BES1/BZR1, which results in the binding of BES1/BZR1 to Brassinosteroid Response Element (BRRE)/E‐box‐containing promoters. This binding regulates the expression of numerous BR‐responsive genes crucial for plant growth and development. (B and C) We analysed the effects of progesterone and testosterone on the root length in loss‐of‐function lines of BR1 (*bri1*), as well as BSK1 (*bsk1*). Both lines showed the typical dwarf phenotype, caused by inhibited brassinosteroid signalling. When germinated on progesterone‐ or testosterone‐containing medium, these lines showed an additional, and statistically significant, reduction in root length. The graphs give mean ± SEM. Statistical differences, indicated by asterisks (*P ≤ 0.05; **P ≤ 0.01; ***P ≤ 0.001), were determined by one‐way ANOVA and the Turkey test. (D) Phenotypes of treated *bsk1* and *bri1* lines, as well as wild type (WT) were documented for progesterone‐treated, testosterone‐treated, mock‐treated and untreated plants.

**Table S1.** Details of analysis of steroids by LC–MS/MS. A volume of 2 μL was injected into an Agilent 1260 Infinity II LC system, consisting of a binary pump G7112B, an autosampler G7167A and a column thermostat G7116A (Agilent Technologies, Santa Clara, CA, USA) without preconcentration or filtering. Chromatographic separation was carried out on a ZORBAX Eclipse XDB‐C18 column (50 × 4.6 mm, 1.8 μm) from Agilent Technologies (Santa Clara, CA, USA). A binary solvent system was used as mobile phase consisting of (A) 0.05% formic acid in water and (B) acetonitrile with a constant flow rate of 1.1 mL/min at 20°C column temperature. The following gradient was applied: 0.00–0.50 min, 60% A; 0.50–5.00 min, 60% to 10% A; 5.00–5.05 min, 10% to 0% A; 5.05–6.50 min, 0% A; 6.50–6.55 min, 0%–60 % A; 6.55–9.00 min, 60% A. The column outlet was connected to a QTRAP 6500+ triple quadrupole mass spectrometer (AB Sciex LLC, Framingham, MA, USA). The Turbo Spray IonDrive ion source was running in positive ionisation mode with 5500 V ion spray voltage and 650 °C turbo gas temperature. The curtain gas was set to 40 psi; the collision gas to ‘medium’ and both ion source gases 1 and 2 were set to 70 psi. Scheduled multiple reaction monitoring (scheduled MRM) was used to monitor analyte parent ion → product ion fragmentations as described in Table 1. Q1 and Q3 quadrupoles were maintained at unit resolution. Analyst 1.6 software (Applied Biosystems) was used for data acquisition and processing. Nona‐deuterated progesterone (PO‐*d*_9_) was used as internal standard (IS) for quantification. The response factors (analyte × standard^−1^) of individual steroids relative to the internal standard have been experimentally determined. The table shows mass‐to‐charge ratio (*m/z*), retention time (RT), collision energy (CE) and the response factor to the used internal standard (*f*).

**Table S2.** Sequences of steroidogenesis from plants without available genomes. Transcripts from the following NCBI bioprojects were analysed for species without available genome sequences.

**Table S3.** List of packages needed for conducting the statistical root length analysis in Rstudio.

**Table S4.** Brassinosteroid profiles of progesterone‐ or testosterone‐treated *Arabidopsis thaliana*. Here, we show brassinosteroid profiles of shoot and root tissues of wild‐type plants (*A. thaliana* Columbia‐0) and plants overexpressing DET2 (*A. thaliana DET2* OE). In addition to the brassinosteroid profiles of these plant lines, we analysed putative changes in the brassinosteroid profiles caused by progesterone (PO) or testosterone (TO) treatment. For this experiment, we treated wild‐type plants (Col. 0) and the transgenic line (DET2 OE L4) with PO and TO and analysed the resulting brassinosteroid levels after 4 days. Each value represents a separate biological replicate which was methodologically divided into technical replicates (*n* ≥ 3), resulting in the listed SD. MS—plants grown on MS medium (mock treatment); DMSO—MS medium with DMSO (untreated control); PO—MS medium containing 0.03 mM progesterone; TO—MS medium with 0.03 mM testosterone. Col‐0—*A. thaliana* Columbia‐0; DET2 OE L2 – L7—*A. thaliana DET2* OE lines 2–7; NQ—no quantification possible.

**Table S5.** RNAseq raw data progesterone versus DMSO.

**Table S6.** RNAseq raw data testosterone versus DMSO.

**Table S7.** Brassinosteroid values and statistical analysis.

**Table S8.** Progestogen and androgen levels in *A. thaliana* roots after 8 days of treatment. We here give the levels of progestogens and androgens in *A. thaliana* roots in ng mg^−1^ dry weight after 8 days of treatment with DMSO (mock control), progesterone (30 μM) and testosterone (30 μM). Data are given as mean ± standard deviation; *n* ≤ 3.

## Data Availability

The data that supports the findings of this study are available in the supplementary material of this article.
