# Supplementary material for: Progestogens and androgens influence root morphology of angiosperms in a brassinosteroid‐independent manner
Source: Plant J. 2025 Sep 9;123(5):e70459. doi: 10.1111/tpj.70459 (PMC12419790; doi:10.1111/tpj.70459)
Supplement: Supplementary file 1 — Figure S1. Dose‐dependent effects of steroids on root length of Arabidopsis thaliana. We here show the effects of pregnenolone (PR), progesterone (PO), 5α‐dihydroprogesterone (5α‐DHP), DHEA, androstenedione (AD), testosterone (TO), 5α‐dihydrotestosterone (5α‐DHT) and oestradiol (ER) on the root length of A. thaliana. A. thaliana wild‐type seeds were germinated on MS medium supplemented with these steroids in concentrations between 10 and 30 μM (only for 5α‐DHP, an additional concentration of 60 μM was used). Root length was determined after 9 days. The figure shows the root length in cm (mean ± SEM). Statistical differences, indicated by asterisks (*P ≤ 0.05; **P ≤ 0.01; ***P ≤ 0.001), were determined by one‐way ANOVA and Turkey test. [file TPJ-123-0-s012.pdf]

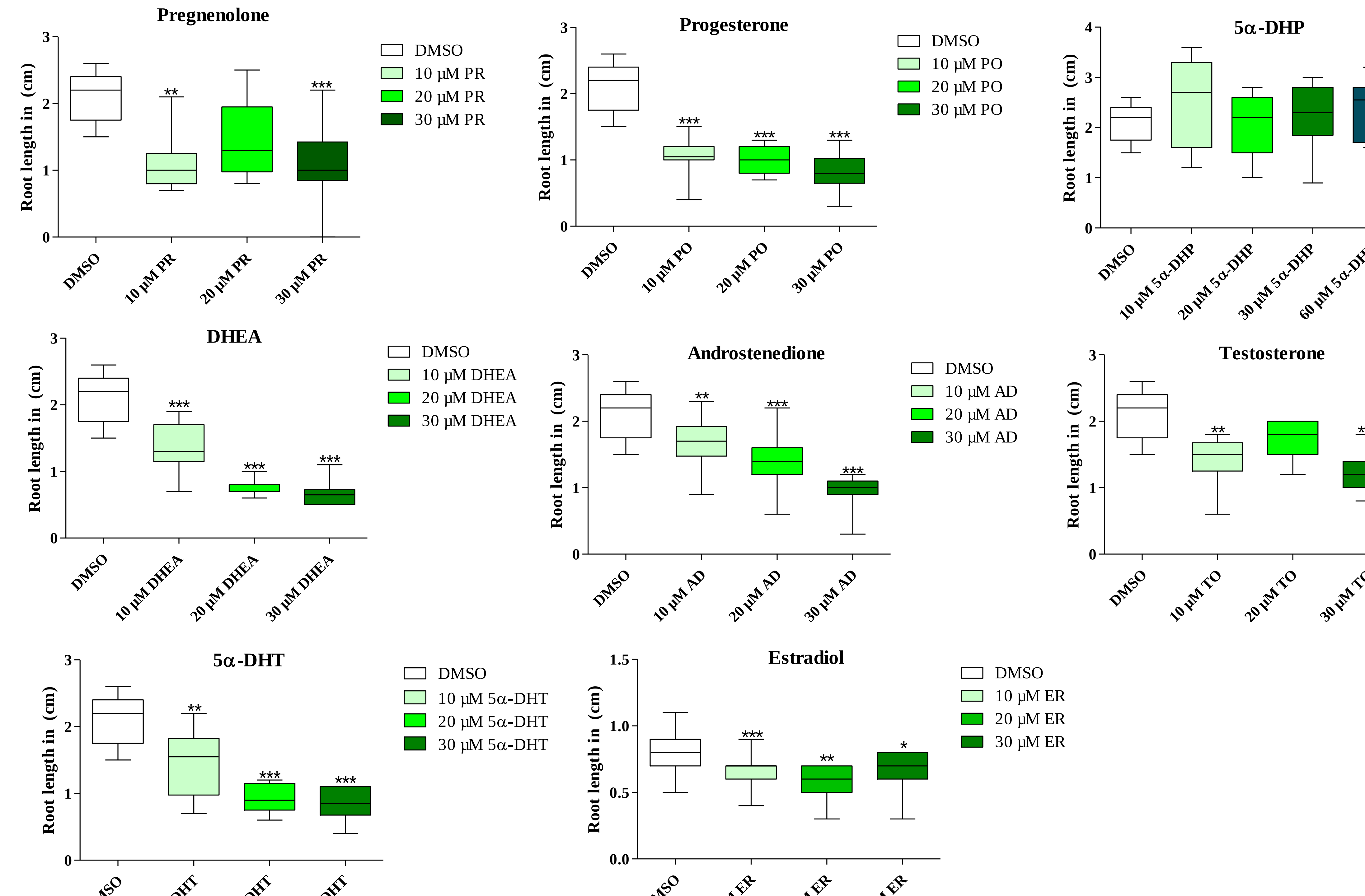

Pregnenolone
Root length in (cm)
DMSO
10 µM PR
20 µM PR
30 µM PR
**
***
Progesterone
DMSO
10 µM PO
20 µM PO
30 µM PO
***
5α-DHP
10 µM 5α-DHP
20 µM 5α-DHP
30 µM 5α-DHP
DHEA
10 µM DHEA
20 µM DHEA
30 µM DHEA
Androstenedione
10 µM AD
20 µM AD
30 µM AD
Testosterone
10 µM TO
20 µM TO
5α-DHT
10 µM 5α-DHT
20 µM 5α-DHT
30 µM 5α-DHT
Estradiol
10 µM ER
20 µM ER
30 µM ER
*

SI Figure S1: Dose-dependent effects of steroids on root length of *Arabidopsis thaliana*. We here show the effects of pregnenolone (PR), progesterone (PO, 5α-dihydroprogesterone (5α-DHP), DHEA, androstenedione (AD), testosterone (TO), 5α-dihydrotestosterone (5α-DHT), and estradiol (ER) on the root length of *A. thaliana*. *A. thaliana* wild-type seeds were germinated on MS medium supplemented with these steroids in concentrations between 10 and 30 µM (only for 5α-DHP an additional concentration of 60 µM was used). Root length was determined after 9 days. The figure shows the root length in cm (mean ± SEM). Statistical differences, indicated by asterisks (* = $p \leq 0.05$; ** = $p \leq 0.01$; *** = $p \leq 0.001$), were determined by one-way ANOVA and Turkey test.
