# Supplementary material for: Progestogens and androgens influence root morphology of angiosperms in a brassinosteroid‐independent manner
Source: Plant J. 2025 Sep 9;123(5):e70459. doi: 10.1111/tpj.70459 (PMC12419790; doi:10.1111/tpj.70459)
Supplement: Supplementary file 2 — Figure S2. Seedling and root morphology of progesterone‐ or testosterone‐treated Brassicaceae species. The figure depicts the morphology of seedlings and roots of the following Brassicaceae species: (A) Aurinia saxatilis (L.) DESV. (B) Barbarea vulgaris W. T. Aiton, (C) Brassica oleracea convar. capitata var. rubra L. cv. Schwarzkopf, (D) Camelina sativa (L.) CRANTZ (E) Cochlearia officinalis L., (F) Diplotaxis tenuifolia (L.) DC., (G) Eruca vesicaria subsp. sativa (L.) CAV., (H) Erysimum cheiri (L.) CRANTZ, (I) Erysimum crepidifolium RCHB., (J) Hesperis matronalis L., (K) Isatis tinctoria L., (L) Lepidium sativum L., (M) Lobularia maritima (L.) DESV. cv. Schneeteppich, (N) Malcolmia maritima (L.) W. T. Aiton, (O) Matthiola incana (L.) W.T. Aiton, (P) Nasturtium officinale W. T. Aiton, (Q) Raphanus sativus var. sativus L. cv. Riesenbutter, (R) Sinapsis alba L., (S) Sisymbrium officinale (L.) SCOP. (a) gives the root lengths of the analysed plant as mean ± SEM. Statistical differences, indicated by asterisks (*P ≤ 0.05; **P ≤ 0.01; ***P ≤ 0.001), were determined by one‐way ANOVA and Tukey test. (b–e) are pictures of the morphology of the seedlings. (f–h) show microscopic pictures of the root tips of the analysed plant. b and f = MS control; c and g = DMSO mock treatment; d and h = 30 μM progesterone; e and h = 30 μM testosterone. Green arrows indicate uncoordinated cell growth, while white arrows indicate enhanced root hair development. [file TPJ-123-0-s003.pdf]

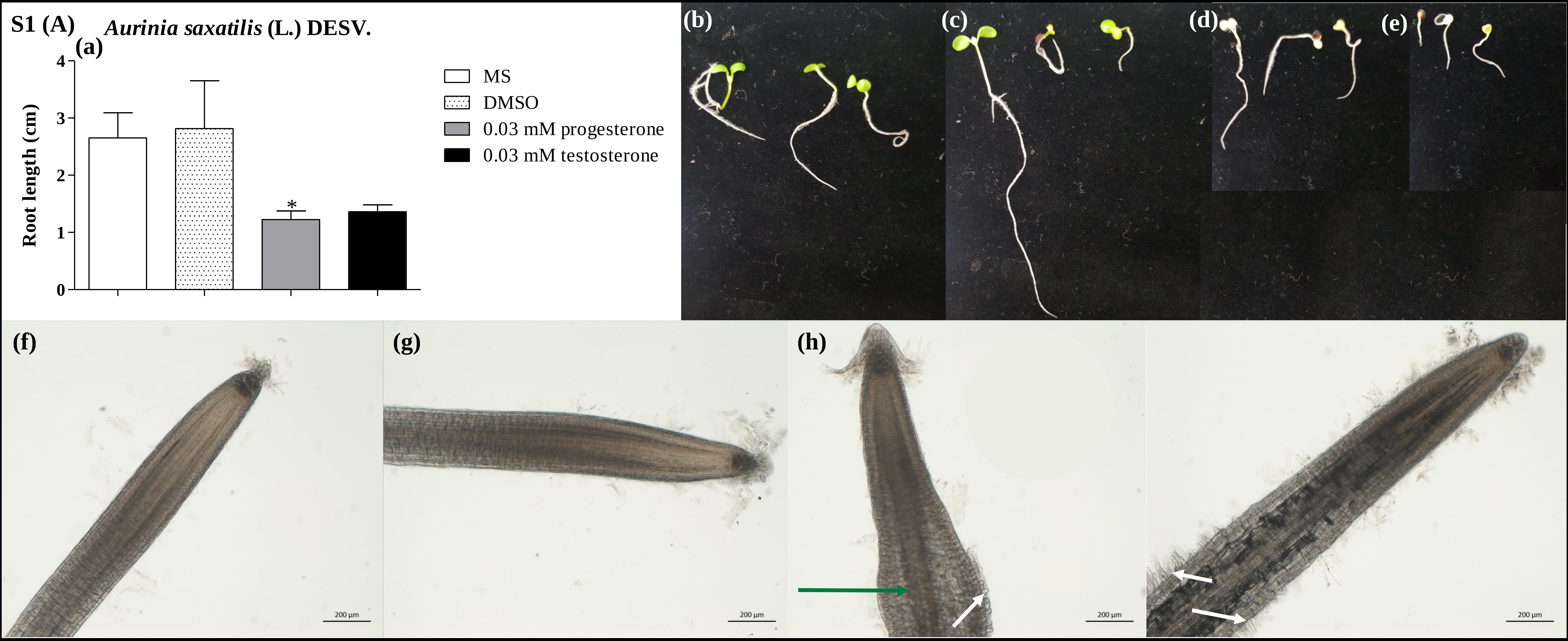
S1 (A)
Aurinia saxatilis (L.) DESV.
(a)
Root length (cm)
0
1
2
3
4
*
MS
DMSO
0.03 mM progesterone
0.03 mM testosterone
(b)
(c)
(d)
(e)
(f)
(g)
(h)
200 µm
200 µm
200 µm
200 µm

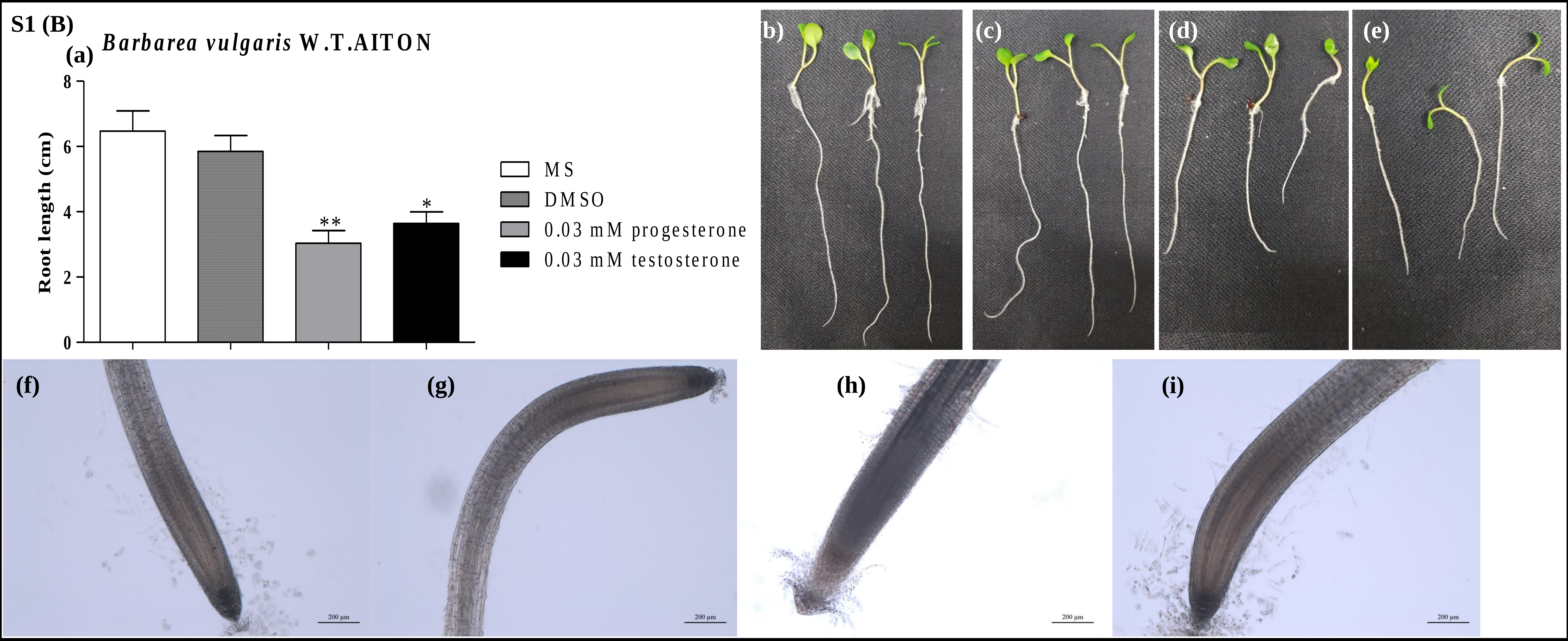
S1 (B)
(a)
Barbarea vulgaris W.T.AITON
Root length (cm)
8
6
4
2
0
**
*
MS
DMSO
0.03 mM progesterone
0.03 mM testosterone
(b)
(c)
(d)
(e)
(f)
(g)
(h)
(i)
200 µm
200 µm
200 µm
200 µm

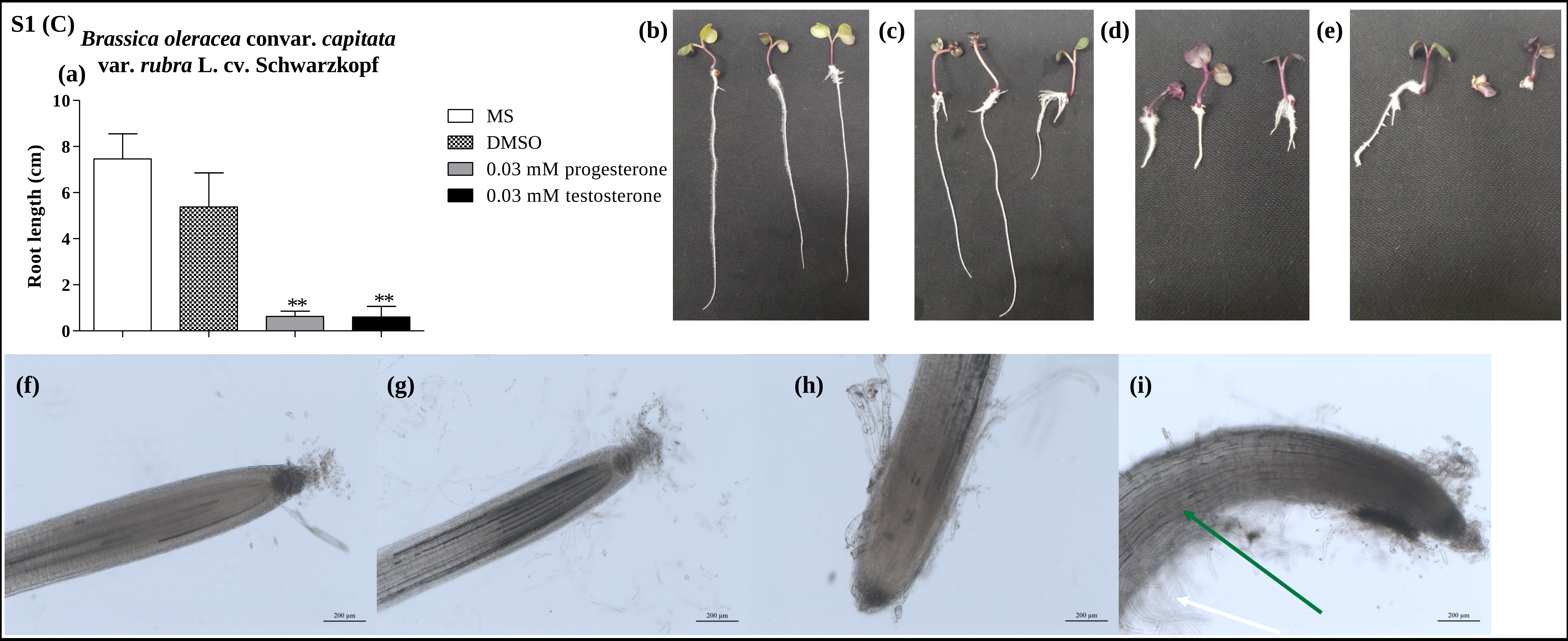
S1 (C)
Brassica oleracea convar. capitata var. rubra L. cv. Schwarzkopf
(a)
Root length (cm)
10
8
6
4
2
0
**
**
MS
DMSO
0.03 mM progesterone
0.03 mM testosterone
(b)
(c)
(d)
(e)
(f)
(g)
(h)
(i)
200 µm
200 µm
200 µm
200 µm

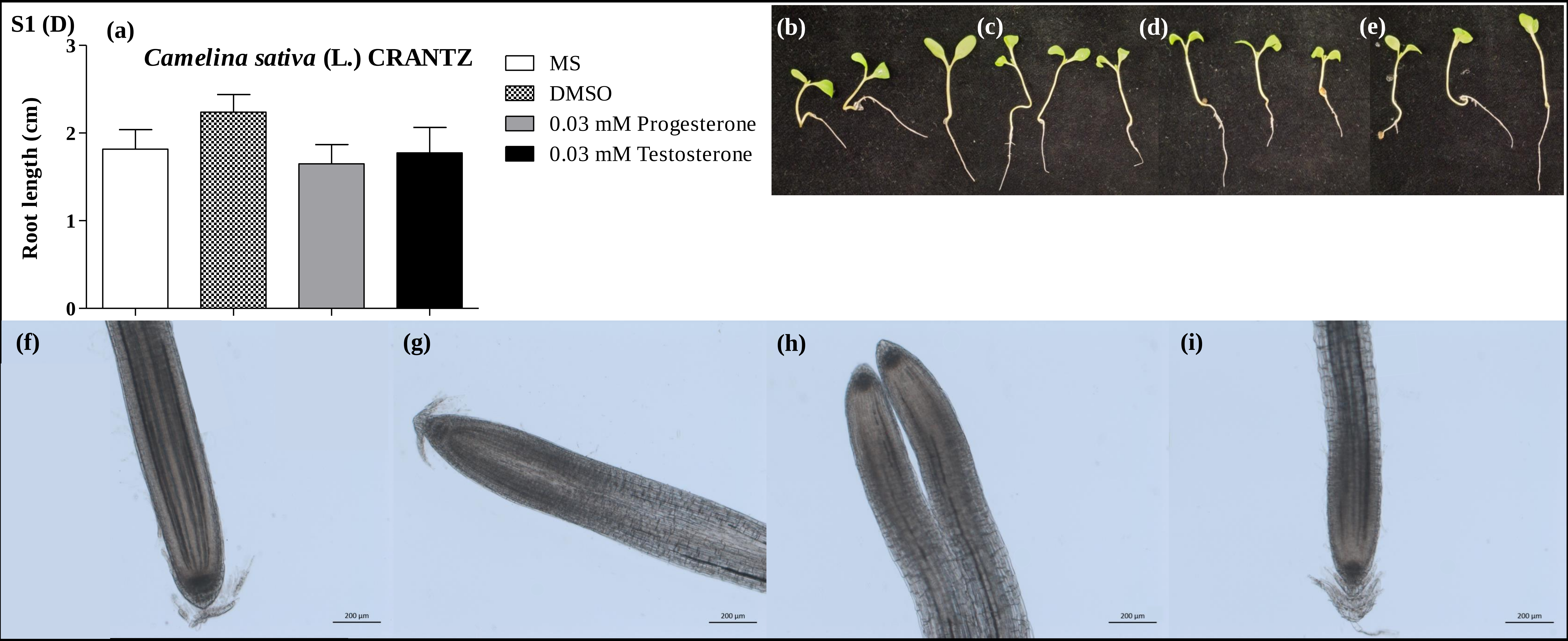

S1 (D)
(a)
Camelina sativa (L.) CRANTZ
Root length (cm)
0
1
2
3
MS
DMSO
0.03 mM Progesterone
0.03 mM Testosterone
(b)
(c)
(d)
(e)
(f)
(g)
(h)
(i)
200 µm
200 µm
200 µm
200 µm

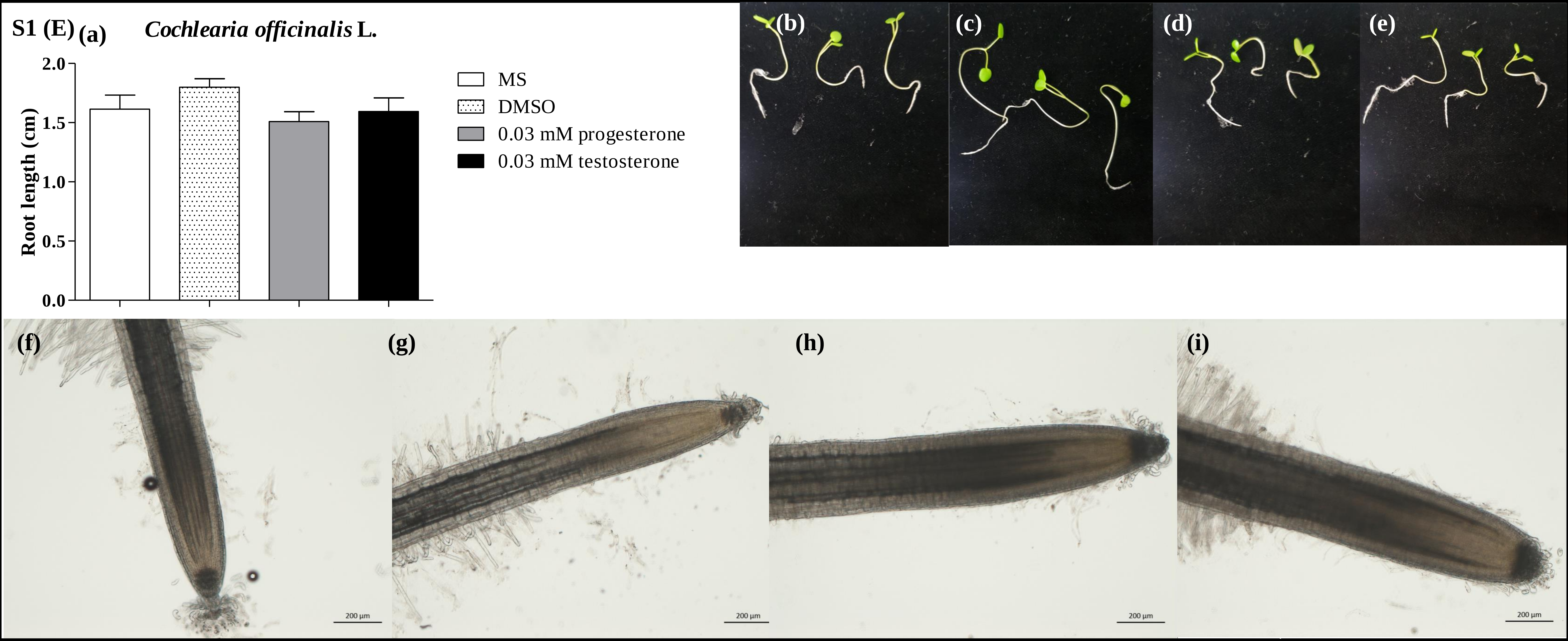

S1 (E) (a) *Cochlearia officinalis* L.
Root length (cm)
2.0
1.5
1.0
0.5
0.0
MS
DMSO
0.03 mM progesterone
0.03 mM testosterone
(b)
(c)
(d)
(e)
(f)
(g)
(h)
(i)
200 µm
200 µm
200 µm
200 µm

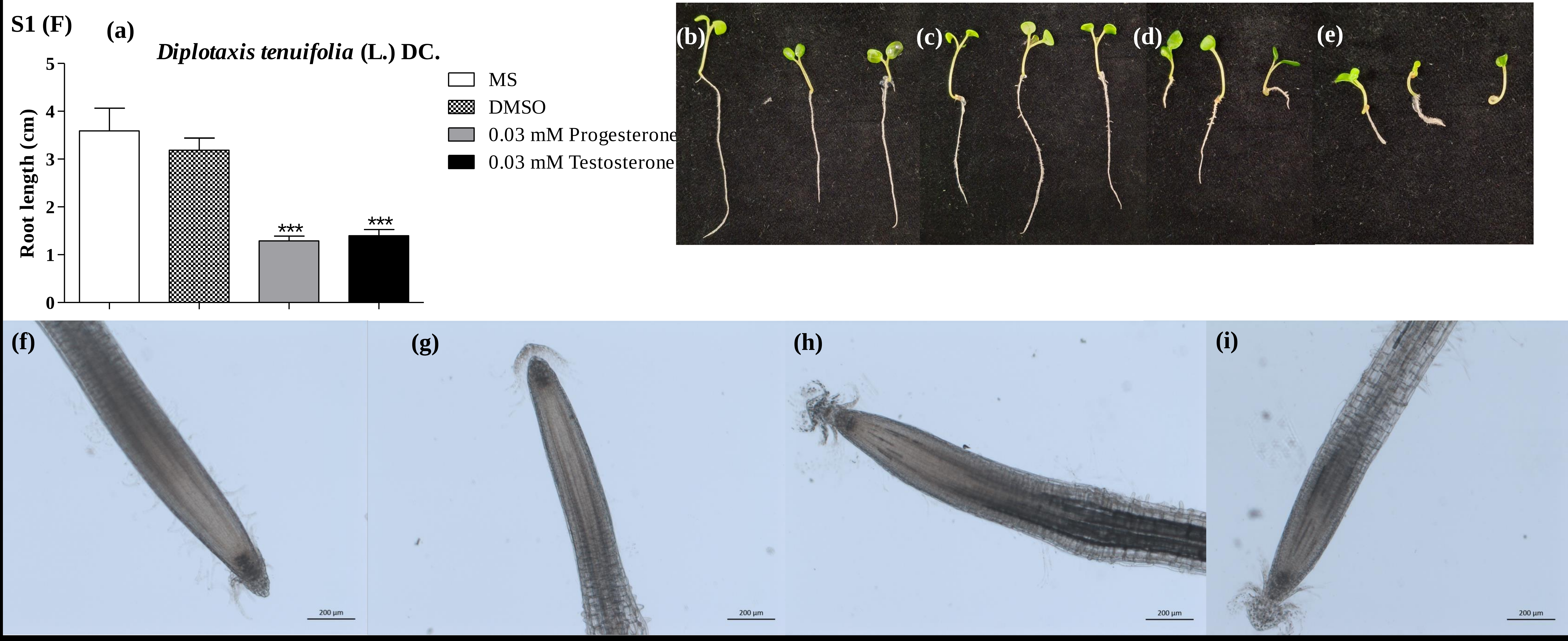
S1 (F)
(a)
Diplotaxis tenuifolia (L.) DC.
Root length (cm)
0
1
2
3
4
5
***
***
MS
DMSO
0.03 mM Progesterone
0.03 mM Testosterone
(b)
(c)
(d)
(e)
(f)
(g)
(h)
(i)
200 µm
200 µm
200 µm
200 µm

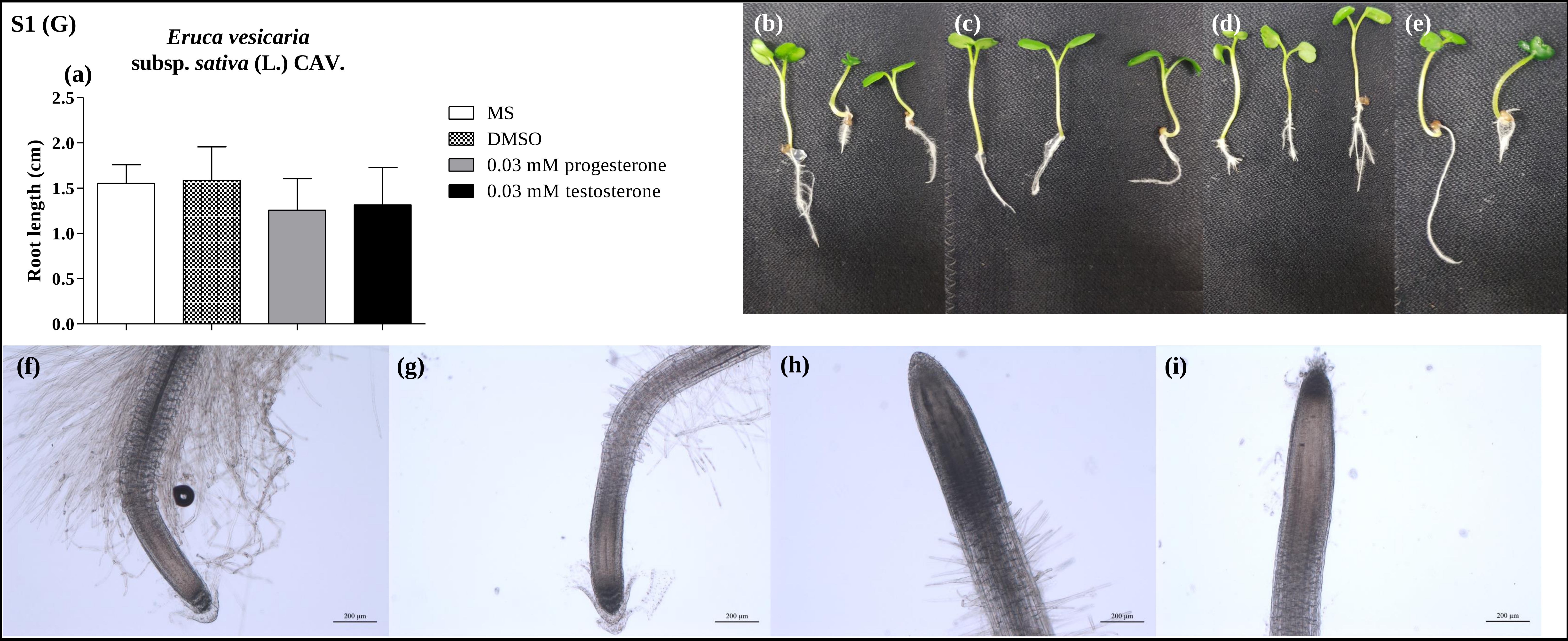
S1 (G)
Eruca vesicaria subsp. *sativa* (L.) CAV.
(a)
Root length (cm)
2.5
2.0
1.5
1.0
0.5
0.0
MS
DMSO
0.03 mM progesterone
0.03 mM testosterone
(b)
(c)
(d)
(e)
(f)
(g)
(h)
(i)
200 µm
200 µm
200 µm
200 µm

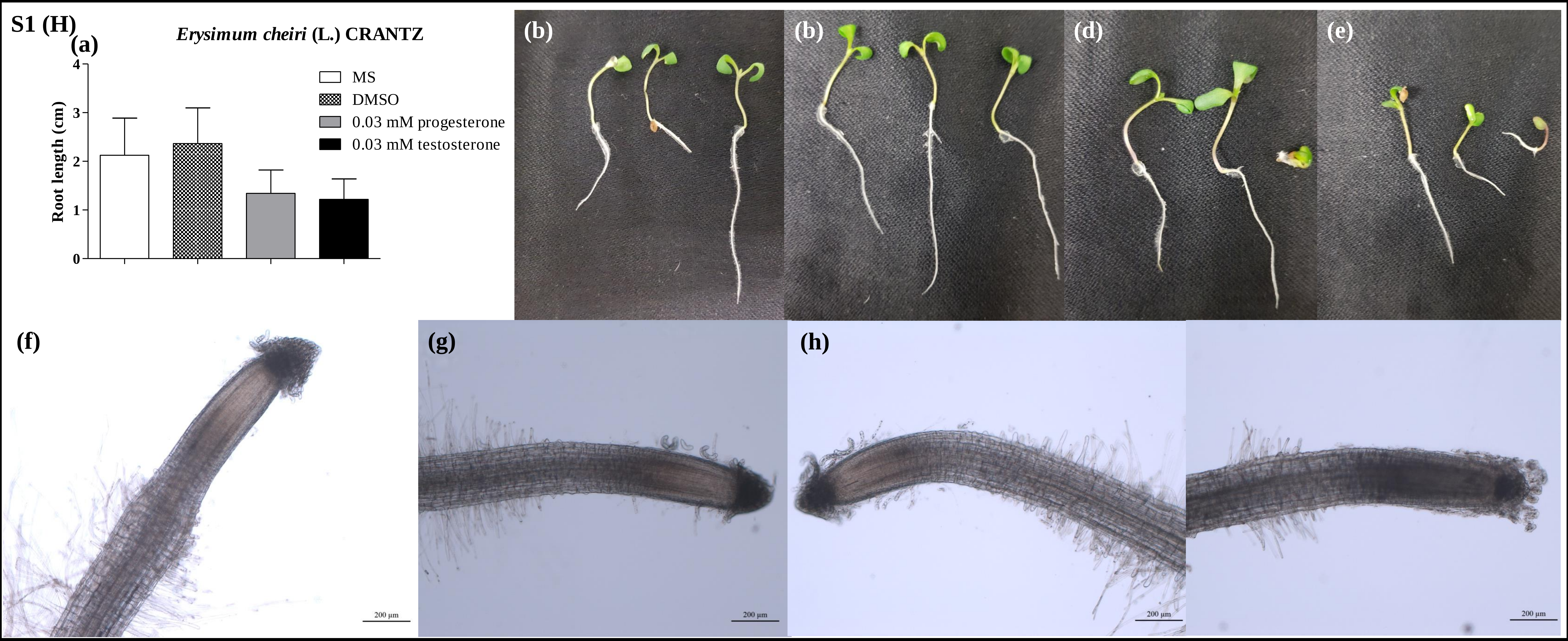
S1 (H)
(a)
Erysimum cheiri (L.) CRANTZ
Root length (cm)
0
1
2
3
4
MS
DMSO
0.03 mM progesterone
0.03 mM testosterone
(b)
(b)
(d)
(e)
(f)
(g)
(h)
200 μm
200 μm
200 μm
200 μm

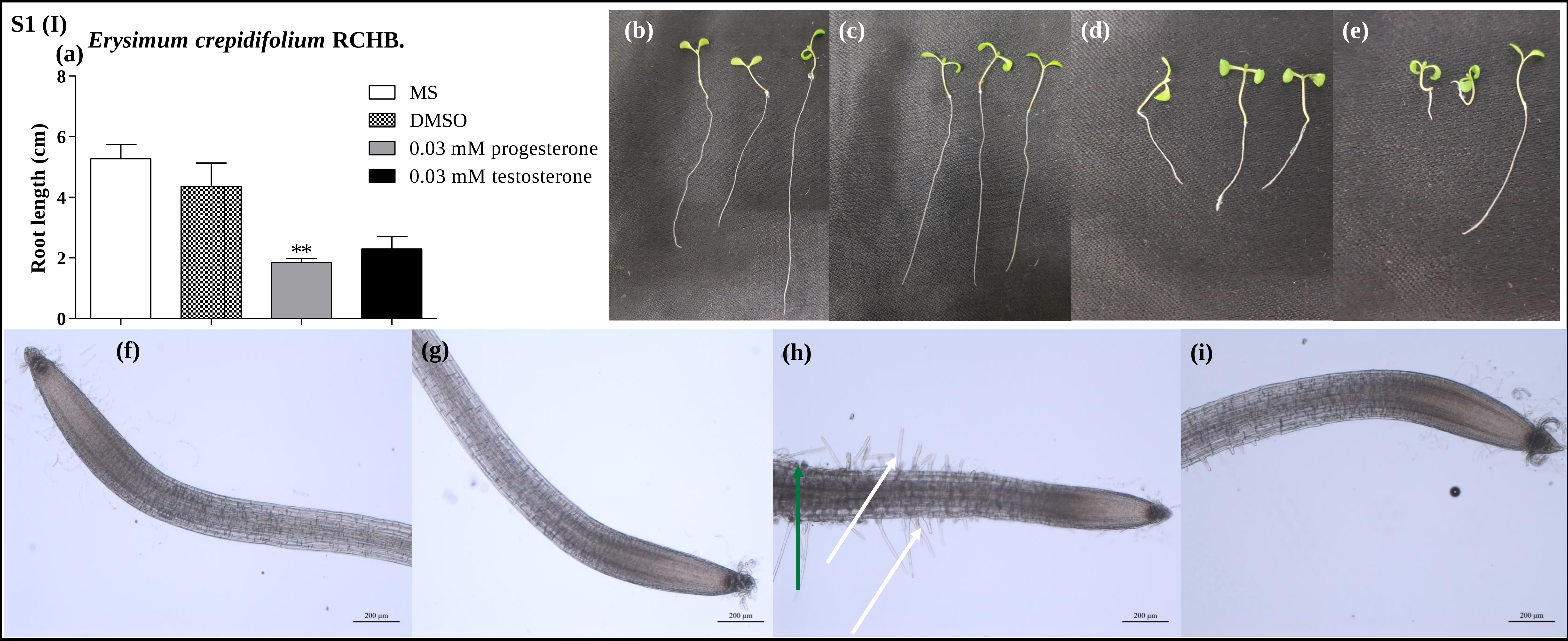
S1 (I)
Erysimum crepidifolium RCHB.
(a)
Root length (cm)
0
2
4
6
8
MS
DMSO
0.03 mM progesterone
0.03 mM testosterone
**
(b)
(c)
(d)
(e)
(f)
(g)
(h)
(i)
200 µm
200 µm
200 µm
200 µm

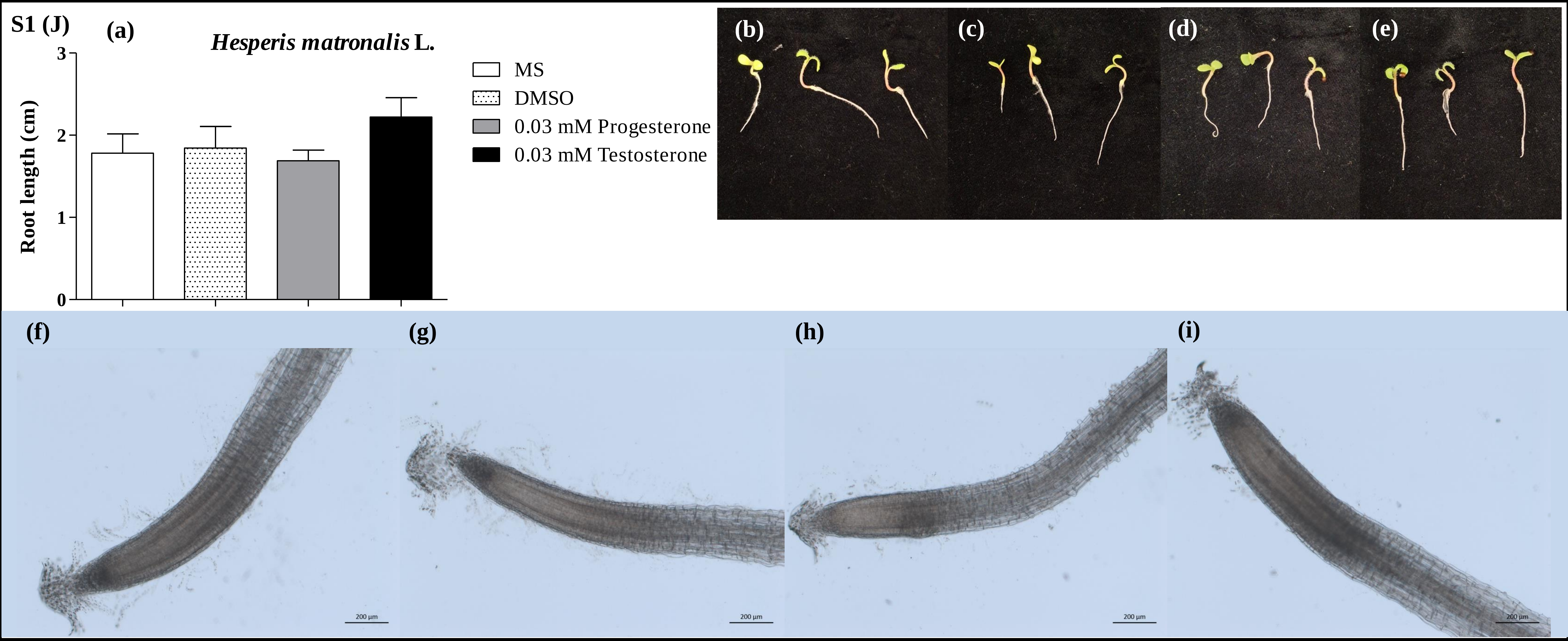
S1 (J)
(a)
Hesperis matronalis L.
Root length (cm)
3
2
1
0
MS
DMSO
0.03 mM Progesterone
0.03 mM Testosterone
(b)
(c)
(d)
(e)
(f)
(g)
(h)
(i)
200 µm
200 µm
200 µm
200 µm

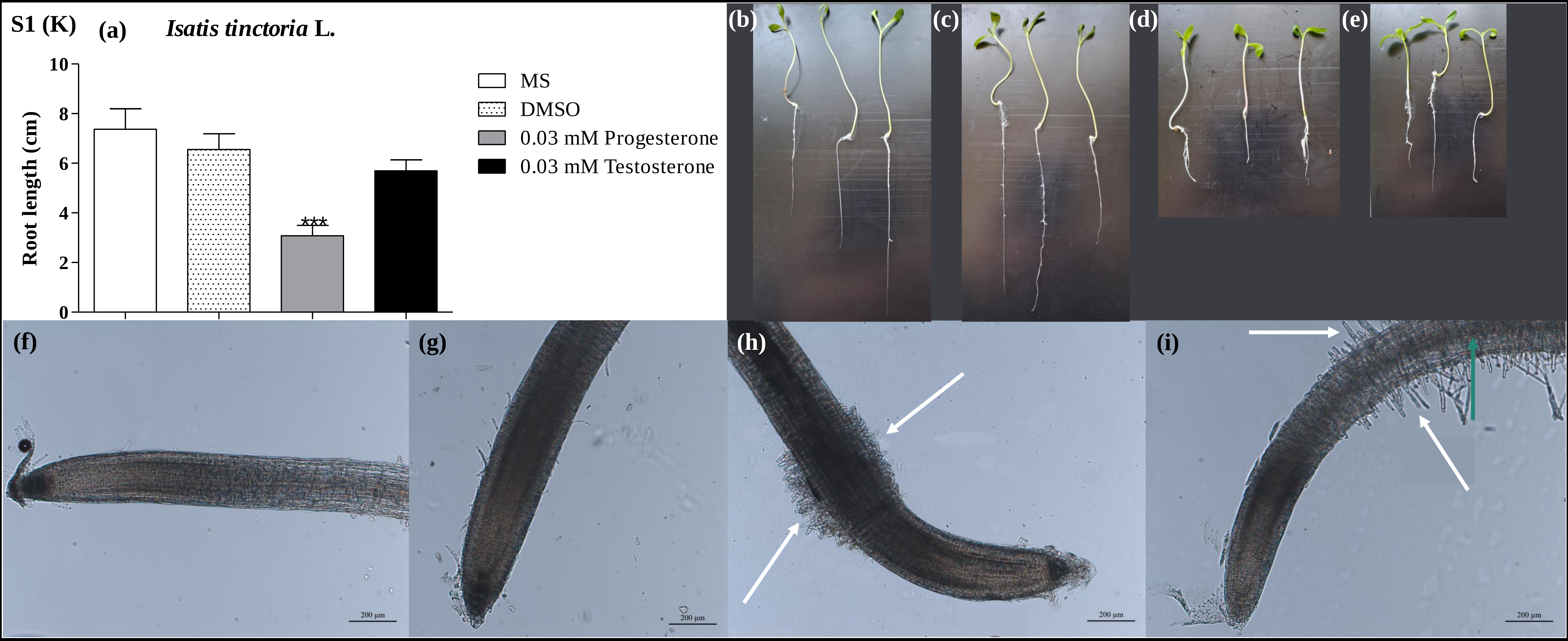
S1 (K)
(a)
Isatis tinctoria L.
Root length (cm)
10
8
6
4
2
0
***
MS
DMSO
0.03 mM Progesterone
0.03 mM Testosterone
(b)
(c)
(d)
(e)
(f)
(g)
(h)
(i)
200 µm
200 µm
200 µm
200 µm

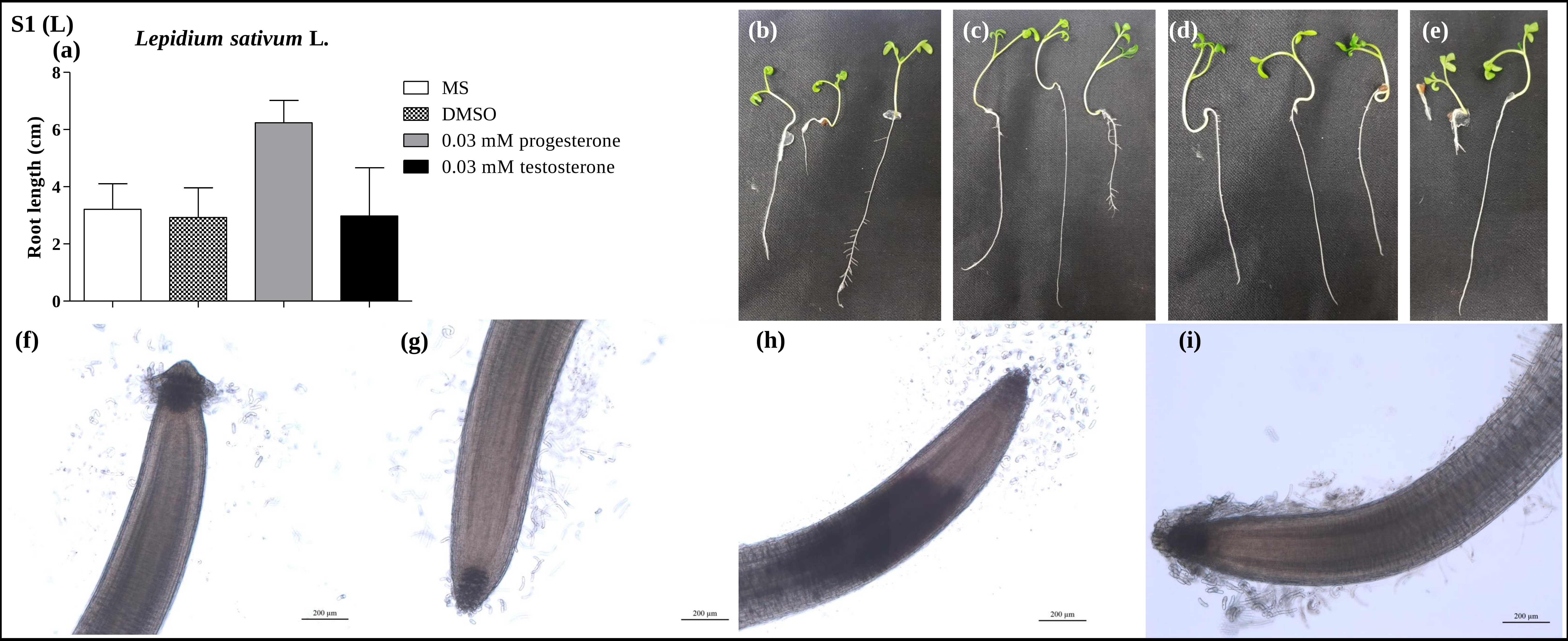

S1 (L)
(a)
Lepidium sativum L.
Root length (cm)
8
6
4
2
0
MS
DMSO
0.03 mM progesterone
0.03 mM testosterone
(b)
(c)
(d)
(e)
(f)
(g)
(h)
(i)
200 µm
200 µm
200 µm
200 µm

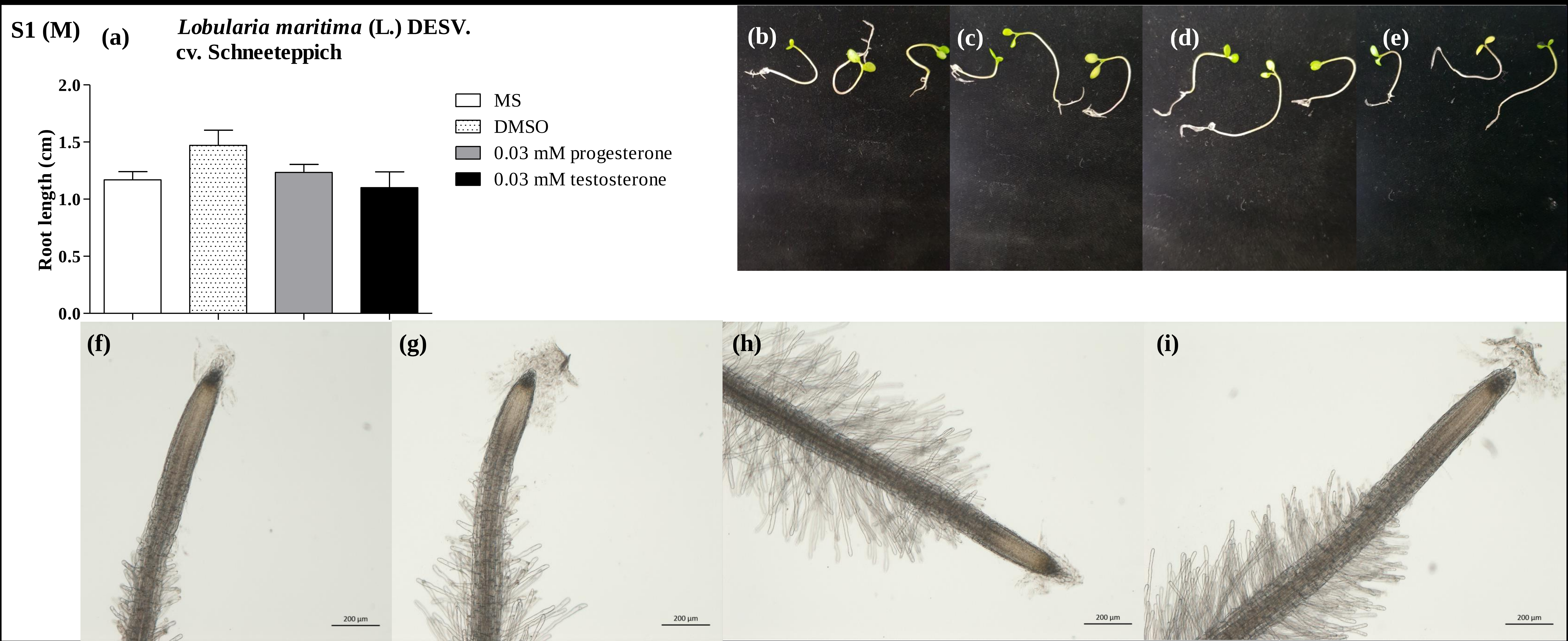
S1 (M)
(a)
Lobularia maritima (L.) DESV.
cv. Schneeteppich
Root length (cm)
2.0
1.5
1.0
0.5
0.0
MS
DMSO
0.03 mM progesterone
0.03 mM testosterone
(b)
(c)
(d)
(e)
(f)
(g)
(h)
(i)
200 µm
200 µm
200 µm
200 µm

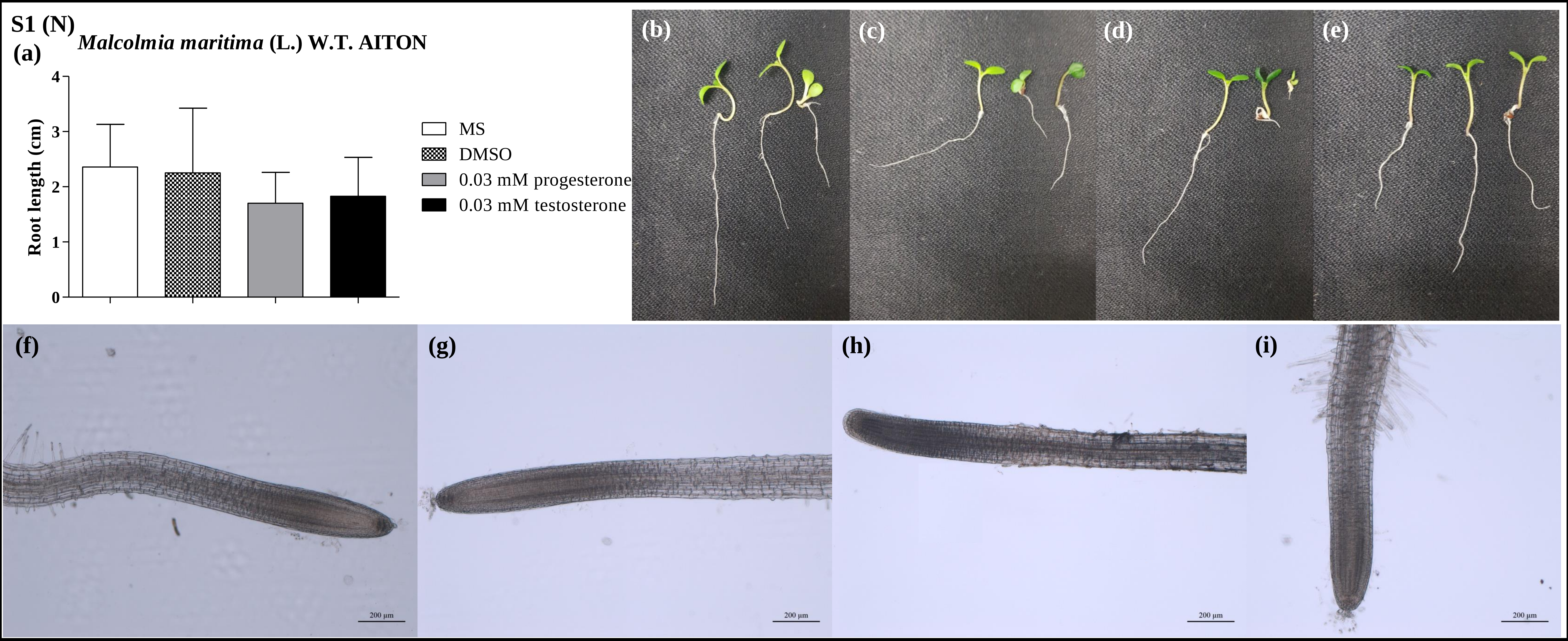
S1 (N)
(a)
Malcolmia maritima (L.) W.T. AITON
Root length (cm)
0
1
2
3
4
MS
DMSO
0.03 mM progesterone
0.03 mM testosterone
(b)
(c)
(d)
(e)
(f)
(g)
(h)
(i)
200 µm
200 µm
200 µm
200 µm

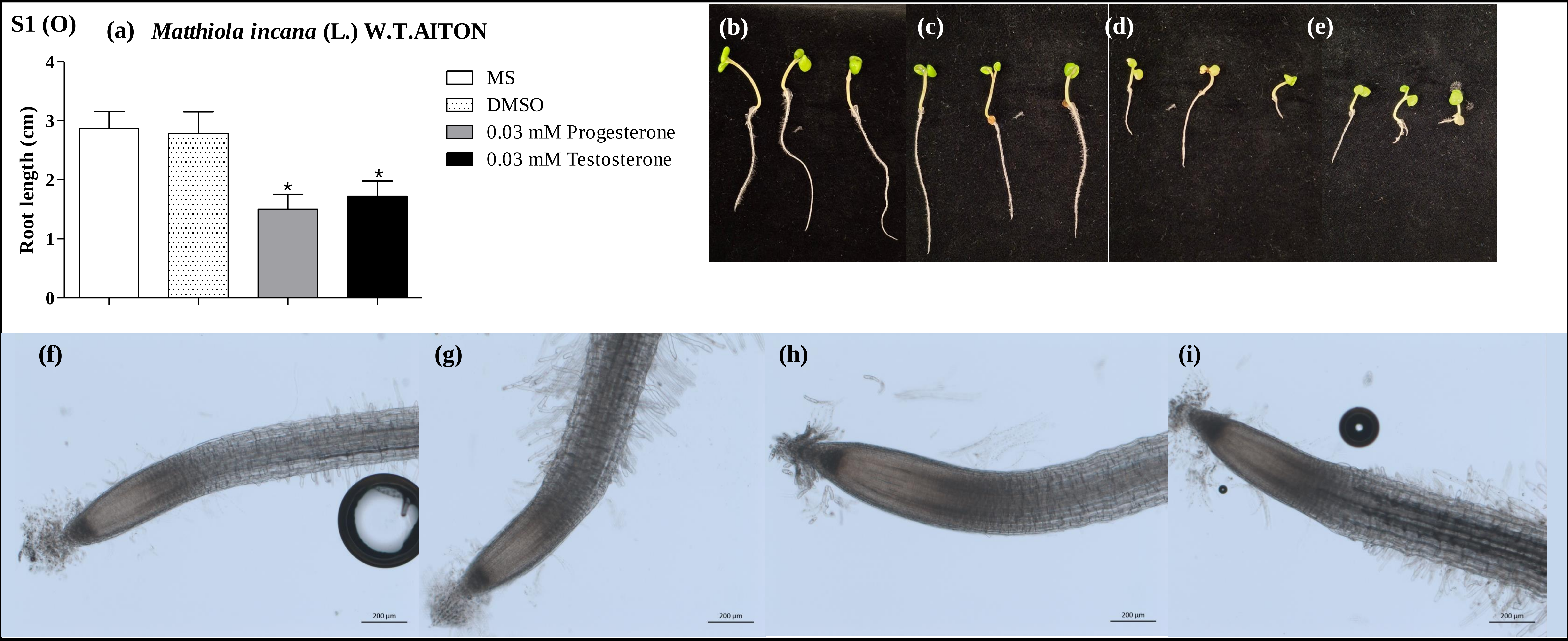

S1 (O)
(a) *Matthiola incana* (L.) W.T.AITON
Root length (cm)
0
1
2
3
4
MS
DMSO
0.03 mM Progesterone
0.03 mM Testosterone
*
*
(b)
(c)
(d)
(e)
(f)
(g)
(h)
(i)
200 µm
200 µm
200 µm
200 µm

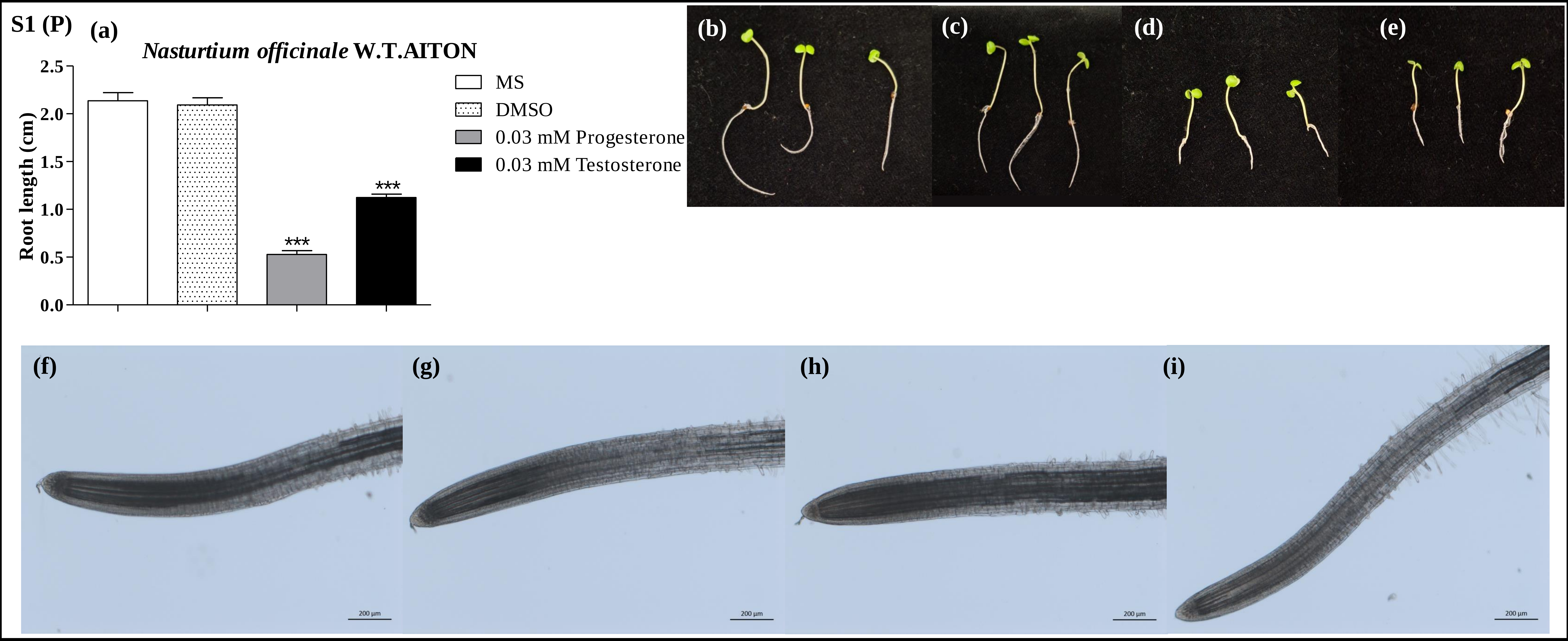
S1 (P)
(a)
Nasturtium officinale W.T.AITON
Root length (cm)
2.5
2.0
1.5
1.0
0.5
0.0
***
***
MS
DMSO
0.03 mM Progesterone
0.03 mM Testosterone
(b)
(c)
(d)
(e)
(f)
(g)
(h)
(i)
200 µm
200 µm
200 µm
200 µm

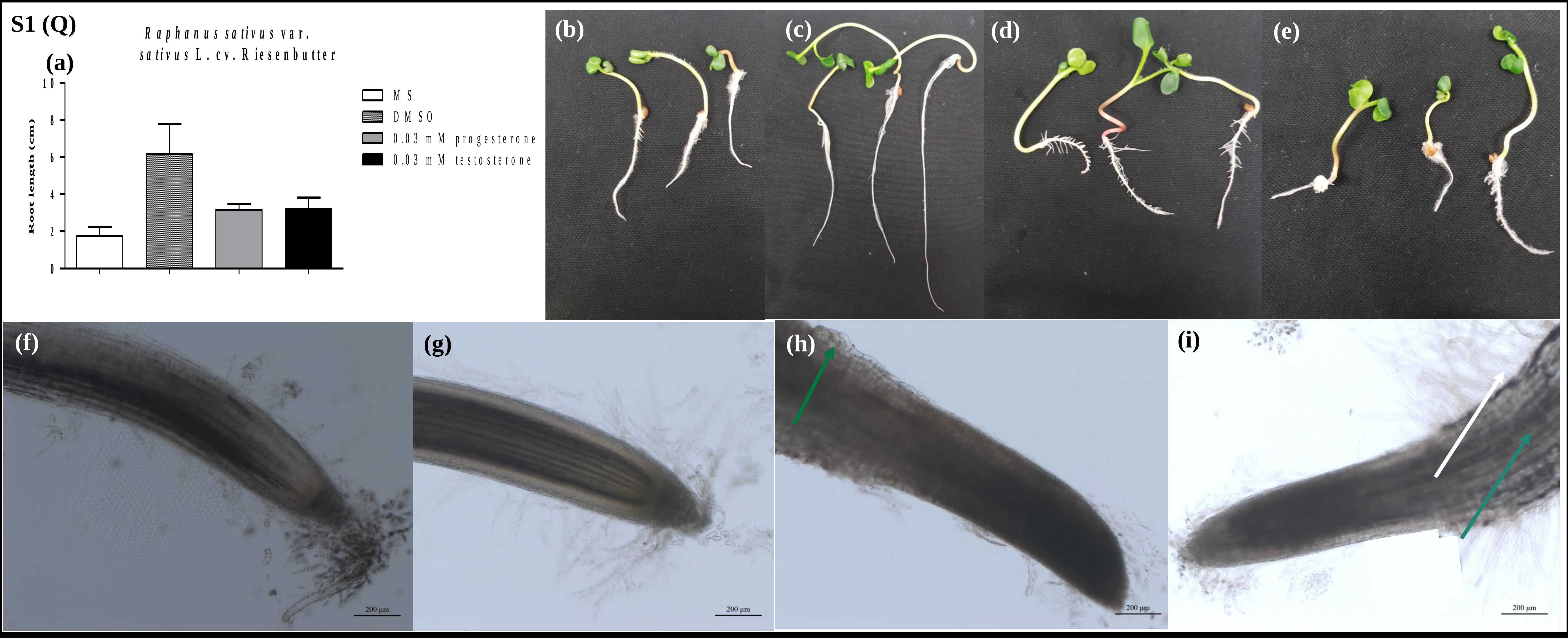
S1 (Q)
Raphanus sativus var. sativus L. cv. Riesenbutter
(a)
Root length (cm)
0
2
4
6
8
10
MS
DMSO
0.03 mM progesterone
0.03 mM testosterone
(b)
(c)
(d)
(e)
(f)
(g)
(h)
(i)
200 µm
200 µm
200 µm
200 µm

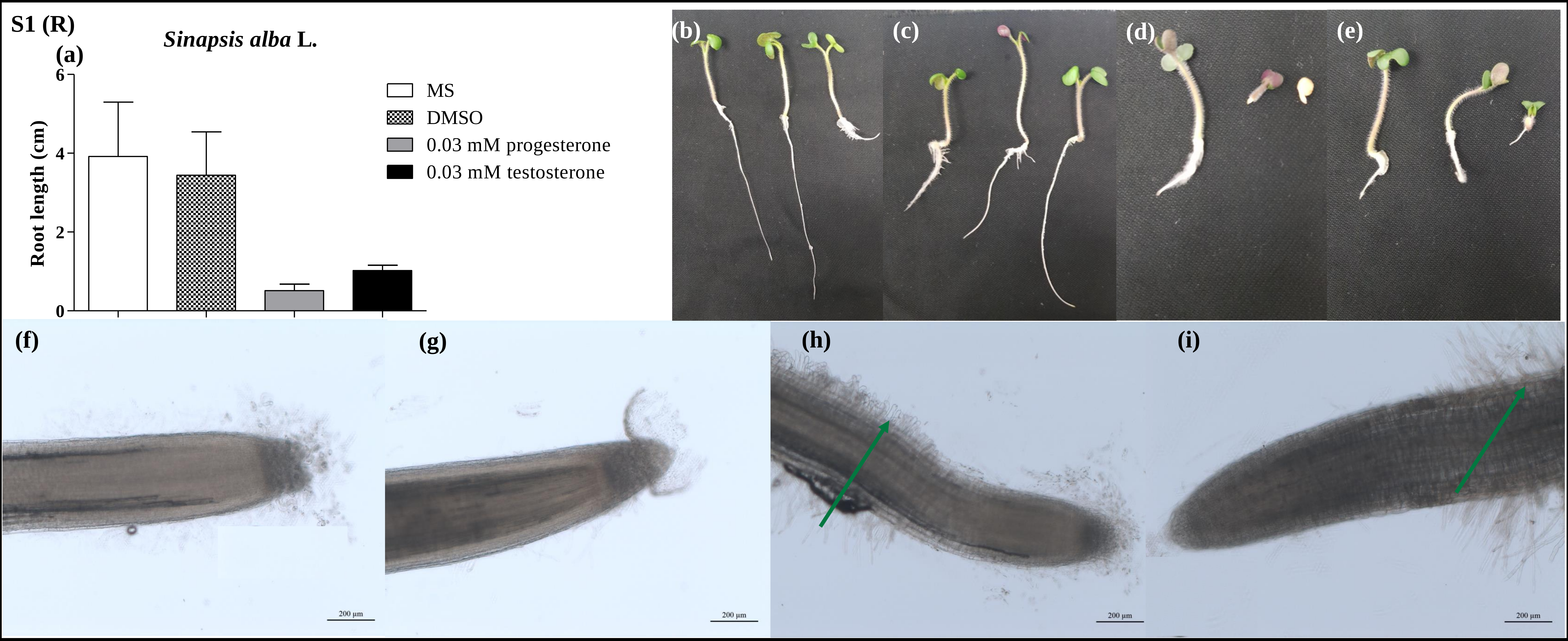

S1 (R)
Sinapsis alba L.
(a)
Root length (cm)
0
2
4
6
MS
DMSO
0.03 mM progesterone
0.03 mM testosterone
(b)
(c)
(d)
(e)
(f)
(g)
(h)
(i)
200 µm
200 µm
200 µm
200 µm

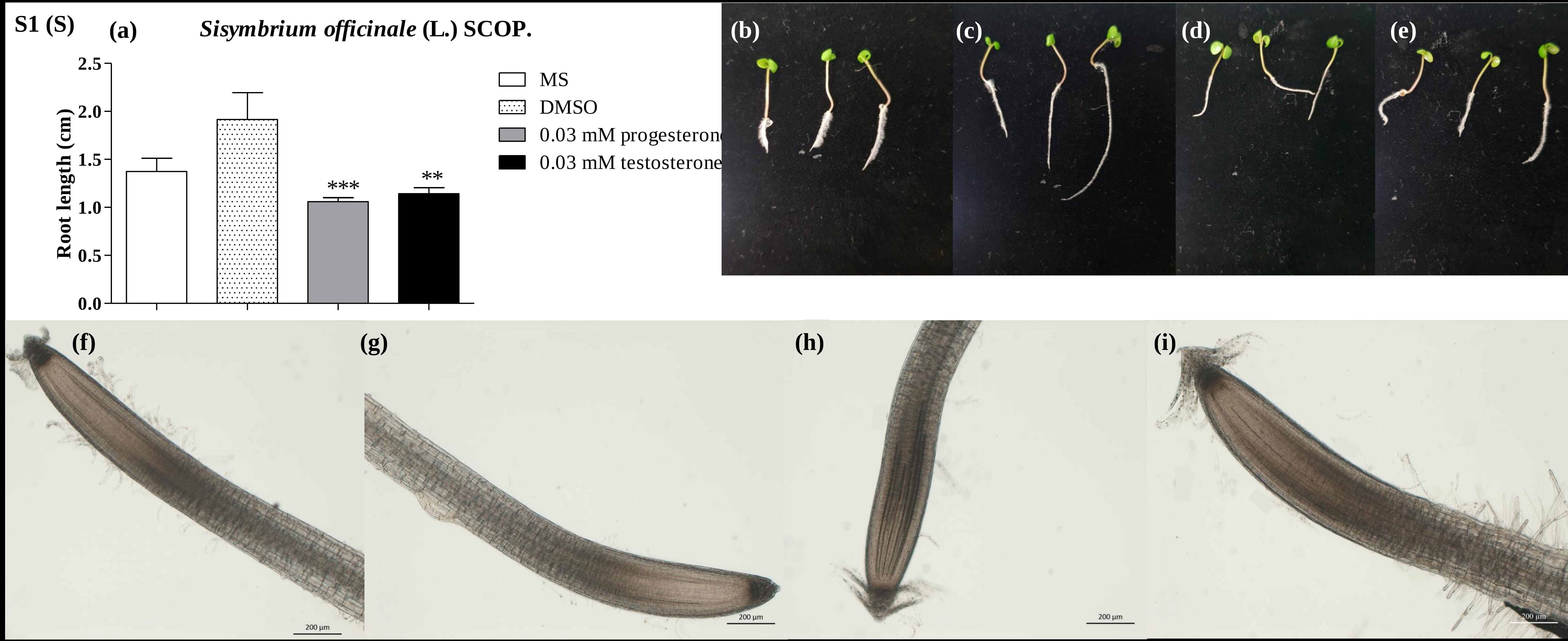
S1 (S)
(a)
Sisymbrium officinale (L.) SCOP.
Root length (cm)
2.5
2.0
1.5
1.0
0.5
0.0
***
**
MS
DMSO
0.03 mM progesterone
0.03 mM testosterone
(b)
(c)
(d)
(e)
(f)
(g)
(h)
(i)
200 µm
200 µm
200 µm
200 µm

SI Figure S2: Seedling and root morphology of progesterone- or testosterone-treated *Brassicaceae* species. The figure depicts the morphology of seedlings and roots of the following *Brassicacea* species: (A) *Aurinia saxatilis* (L.) DESV. (B) *Barbarea vulgaris* W.T.Aiton, (C) *Brassica oleracea* convar. *capitata* var. rubra L. cv. Schwarzkopf, (D) *Camelina sativa* (L.) CRANTZ (E) *Cochlearia officinalis* L., (F) *Diplotaxis tenuifolia* (L.) DC., (G) *Eruca vesicaria* subsp. *sativa* (L.) CAV., (H) *Erysimum cheiri* (L.) CRANTZ, (I) *Erysimum crepidifolium* RCHB., (J) *Hesperis matronalis* L., (K) *Isatis tinctoria* L., (L) *Lepidium sativum* L., (M) *Lobularia maritima* (L.) DESV. cv. Schneeteppich, (N) *Malcolmia maritima* (L.) W.T. Aiton, (O) *Matthiola incana* (L.) W.T.AITON, (P) *Nasturtium officinale* W.T.AITON, (Q) *Raphanus sativus* var. *sativus* L. cv. Riesenbutter, (R) *Sinapsis alba* L., (S*) Sisymbrium officinale* (L.) SCOP. (a) gives the root lengths of the analysed plant as mean ± SEM. Statistical differences, indicated by asterisks (* = $p \leq 0.05$; ** = $p \leq 0.01$; *** = $p \leq 0.001$), were determined by one-way ANOVA and Turkey test. (b – e) are pictures of the morphology of the seedlings. (f – h) show microscopic pictures of the root tips of the analysed plant. b and f = MS control; c and g = DMSO mock treatment; d and h = 30 µM progesterone; e and h = 30 µM testosterone. Green arrows indicate uncoordinated cell growth, while white arrows indicate enhanced root hair development.
