# Supplementary material for: Progestogens and androgens influence root morphology of angiosperms in a brassinosteroid‐independent manner
Source: Plant J. 2025 Sep 9;123(5):e70459. doi: 10.1111/tpj.70459 (PMC12419790; doi:10.1111/tpj.70459)
Supplement: Supplementary file 3 — Figure S3. Seedling and root morphology of progesterone‐ or testosterone‐treated non‐Brassicales angiosperms. The figure depicts the morphology of seedlings and roots of the following Brassicacea species: (A) Nymphaea colorata PETER., (B) Spirodela polyrhiza (L.) SCHLEID., (C) Allium schoenoprasum L. cv. Nelly, (D) Secale cereal L. cv. Dukato, (E) Petroselinum crispum (MILL.) FUSS cv. Mooskrause, (F) Taraxacum officinale L.—wild population Dittelbrunn, (G) Myosotis sylvatica EHRH. EX HOFFM. cv. Heavenly Blue, (H) Beta vulgaris subsp. vulgaris CONDITIVA group L., (I) Cucumis sativus L. cv. Vorgebirgstraube, (J) Valerianella carinata LOISEL., (K) Vaccinium myrtillus L., (L) Trigonella foenum‐graecum L., (M) Centaurium erythraea RAFN., (N) Pelargonium zonale (L.) L'Hér., (O) Digitalis purpurea L., (P) Plantago major L. – wild population Dittelbrunn, (Q) Linum usitatissimum L., (R) Hibiscus sabdariffa L., (S) Oenothera speciosa NUTT., (T) Papaver rhoeas L., (U) Fragaria vesca L. cv. Rügen, (V) Ruta graveolens L. (W) Solanum lycopersicum L. cv. Harzfeuer. (a) gives the root lengths of the analysed plant as mean ± SEM. Statistical differences, indicated by asterisks (*P ≤ 0.05; **P ≤ 0.01; ***P ≤ 0.001), were determined by one‐way ANOVA and Turkey test. (b–e) are pictures of the morphology of the seedlings. (f–h) show microscopic pictures of the root tips of the analysed plant. b and f = MS control; c and g = DMSO mock treatment; d and h = 30 μM progesterone; e and h = 30 μM testosterone. Green arrows indicate uncoordinated cell growth, while white arrows indicate enhanced root hair development. [file TPJ-123-0-s006.pdf]

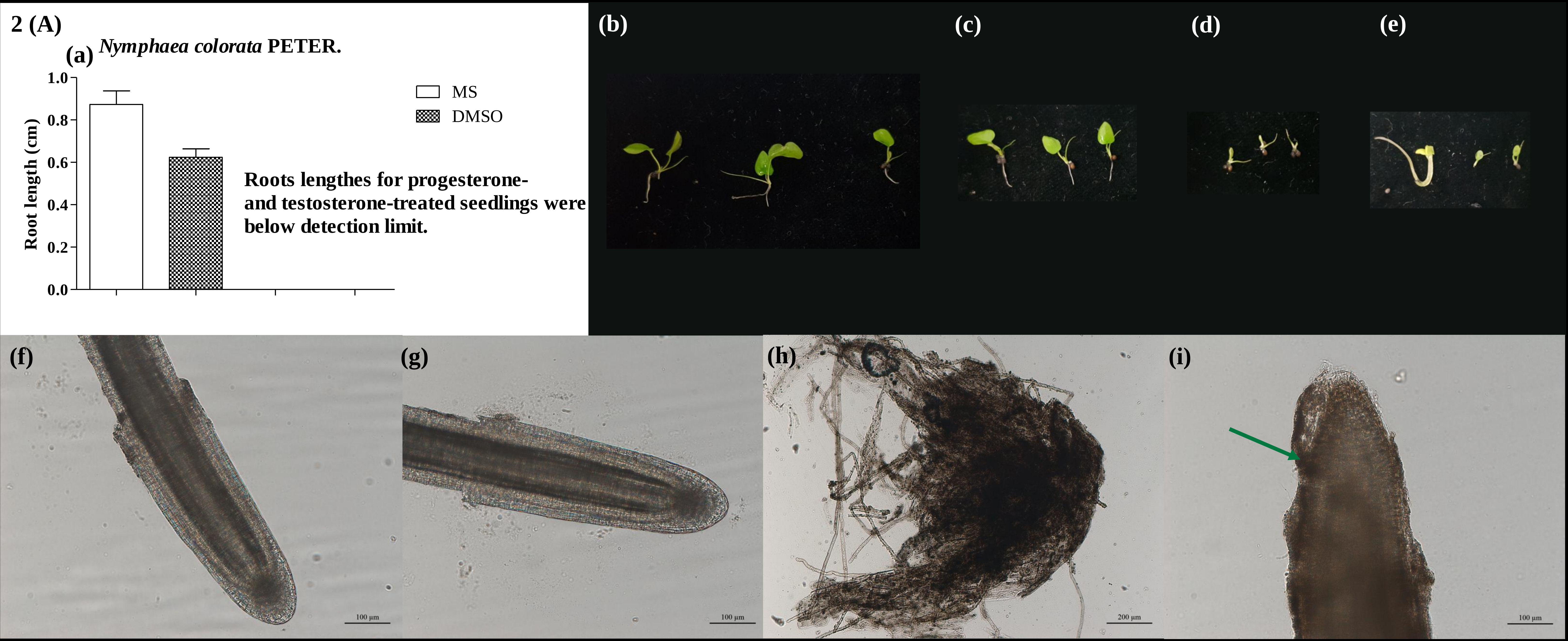
2 (A)
(a) *Nymphaea colorata* PETER.
1.0
0.8
0.6
0.4
0.2
0.0
Root length (cm)
MS
DMSO
Roots lengthes for progesterone- and testosterone-treated seedlings were below detection limit.
(b)
(c)
(d)
(e)
(f)
(g)
(h)
(i)
100 μm
100 μm
200 μm
100 μm

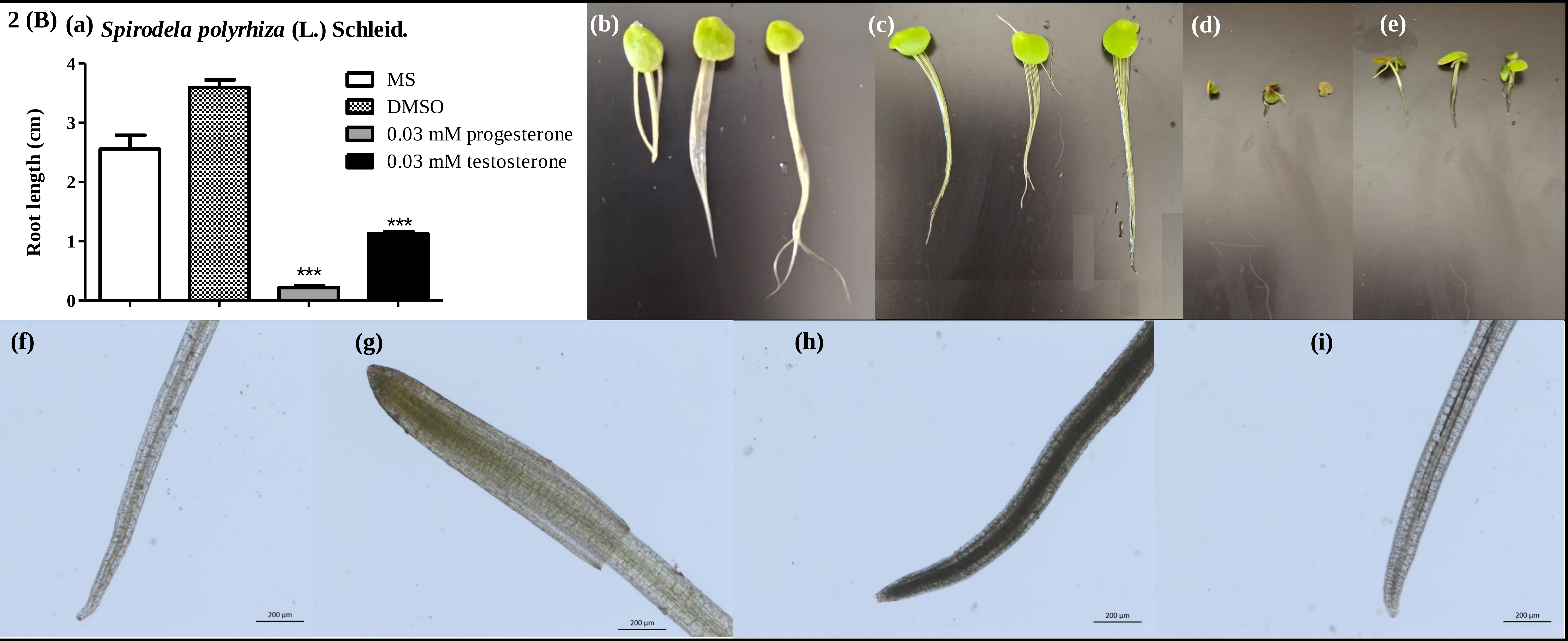
2 (B)
(a) Spirodela polyrhiza (L.) Schleid.
Root length (cm)
0
1
2
3
4
MS
DMSO
0.03 mM progesterone
0.03 mM testosterone
***
***
(b)
(c)
(d)
(e)
(f)
(g)
(h)
(i)
200 µm
200 µm
200 µm
200 µm

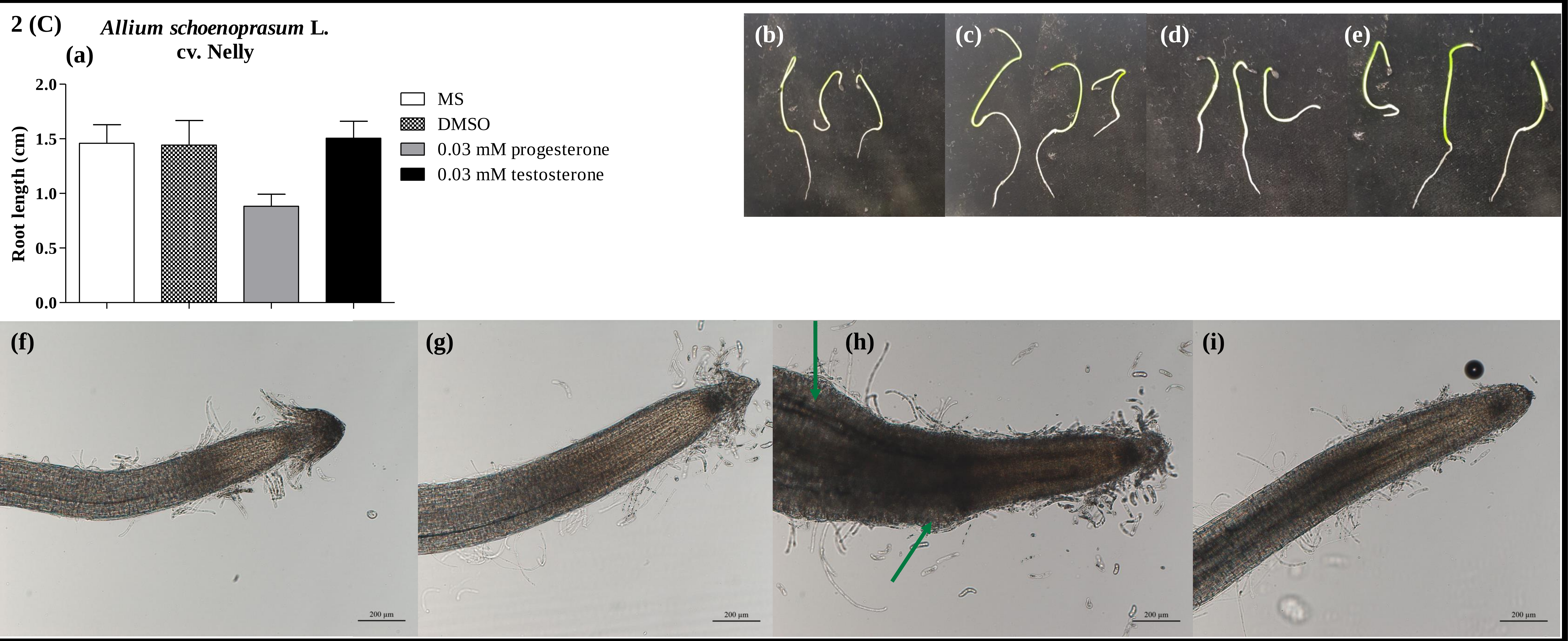
2 (C)
Allium schoenoprasum L.
cv. Nelly
(a)
2.0
1.5
1.0
0.5
0.0
Root length (cm)
MS
DMSO
0.03 mM progesterone
0.03 mM testosterone
(b)
(c)
(d)
(e)
(f)
(g)
(h)
(i)
200 μm
200 μm
200 μm
200 μm

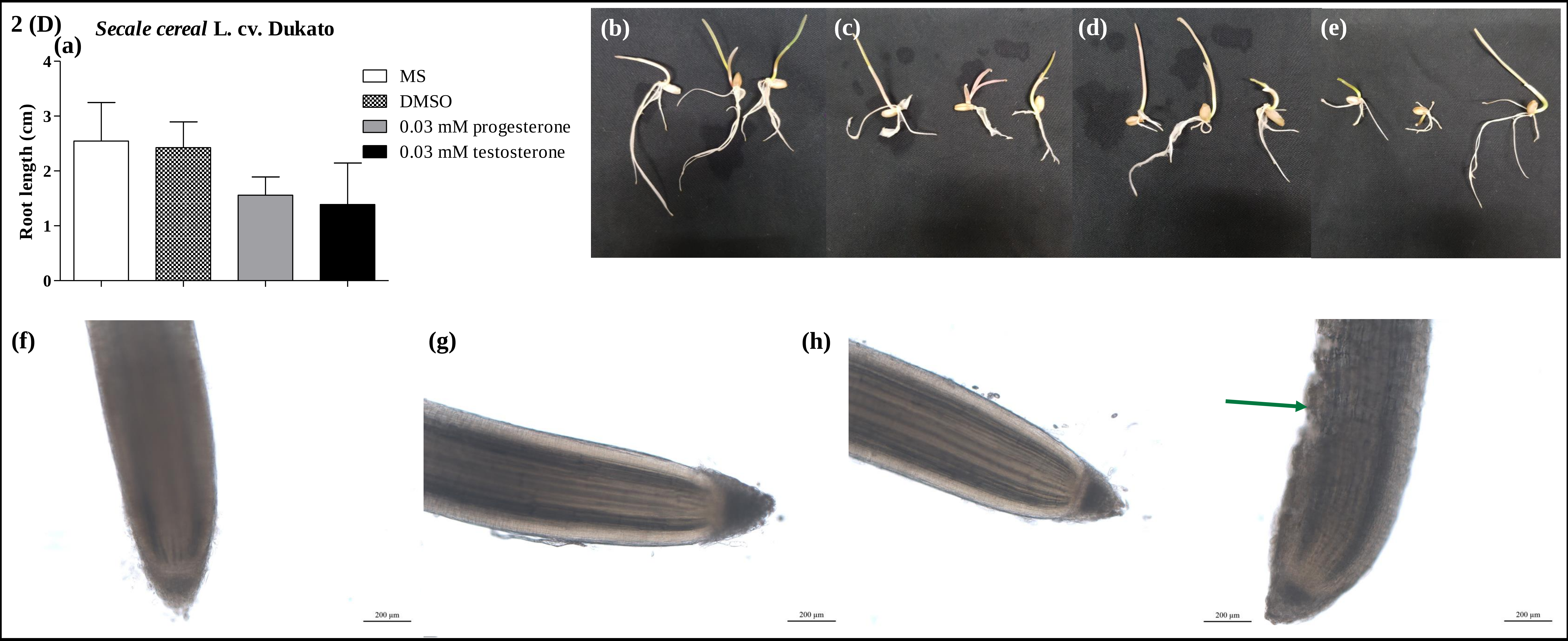

2 (D)
Secale cereal L. cv. Dukato
(a)
Root length (cm)
4
3
2
1
0
MS
DMSO
0.03 mM progesterone
0.03 mM testosterone
(b)
(c)
(d)
(e)
(f)
(g)
(h)
200 µm
200 µm
200 µm
200 µm

2 (E)

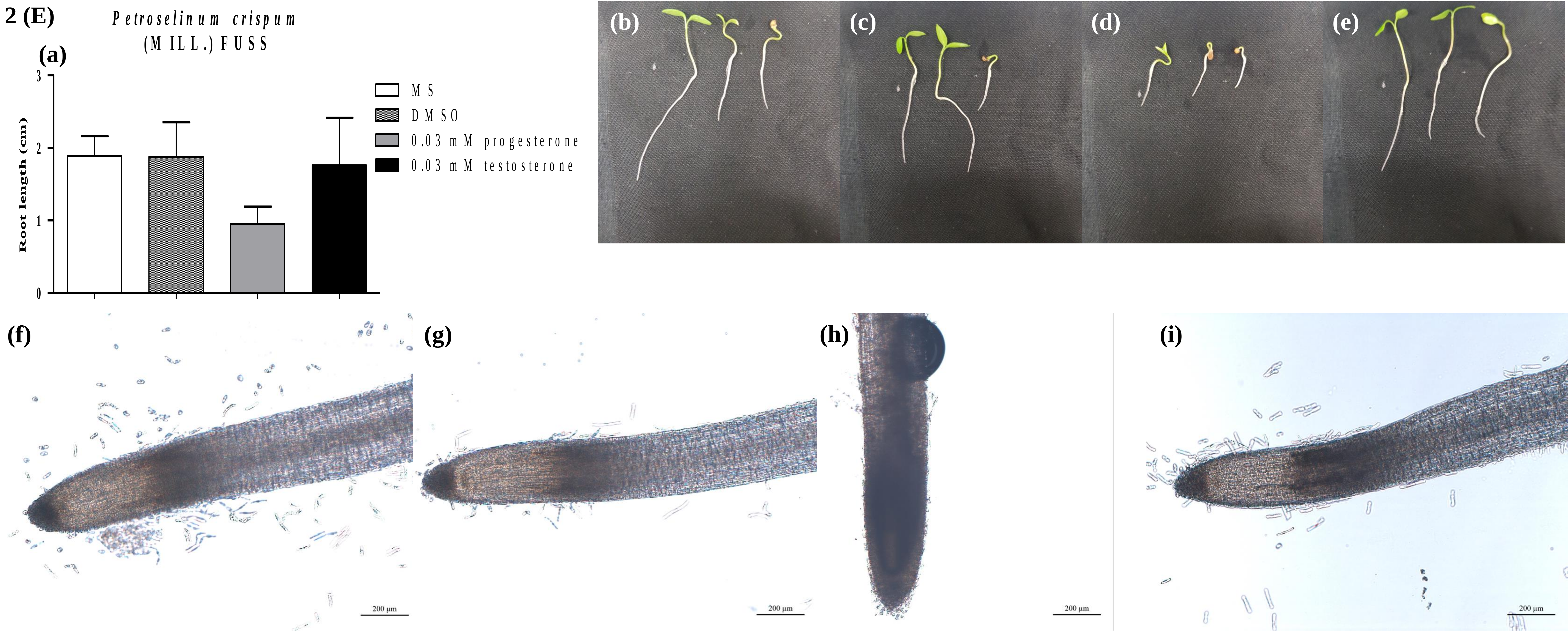

Petroselinum crispum
(MILL.) FUSS
(a)
Root length (cm)
3
2
1
0
MS
DMSO
0.03 mM progesterone
0.03 mM testosterone
(b)
(c)
(d)
(e)
(f)
(g)
(h)
(i)
200 µm
200 µm
200 µm
200 µm

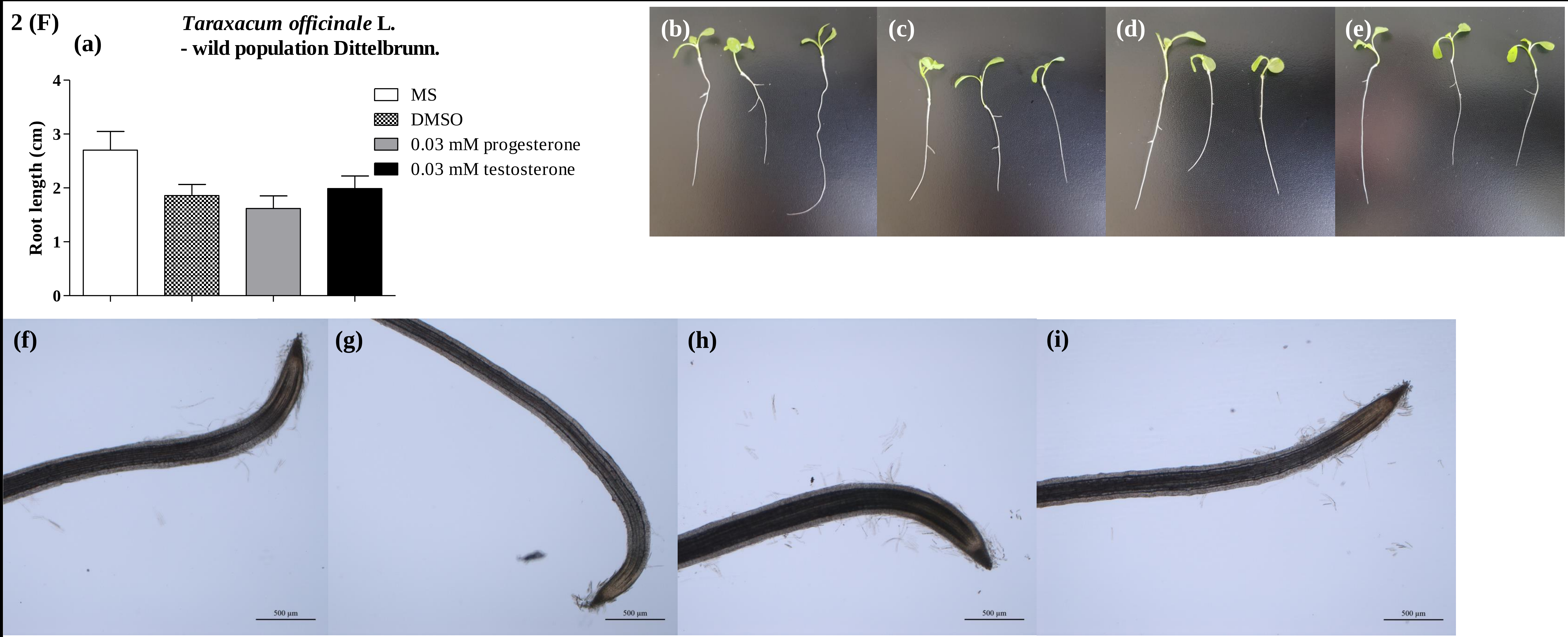

2 (F)
(a)
Taraxacum officinale L.
- wild population Dittelbrunn.
Root length (cm)
4
3
2
1
0
MS
DMSO
0.03 mM progesterone
0.03 mM testosterone
(b)
(c)
(d)
(e)
(f)
(g)
(h)
(i)
500 µm
500 µm
500 µm
500 µm

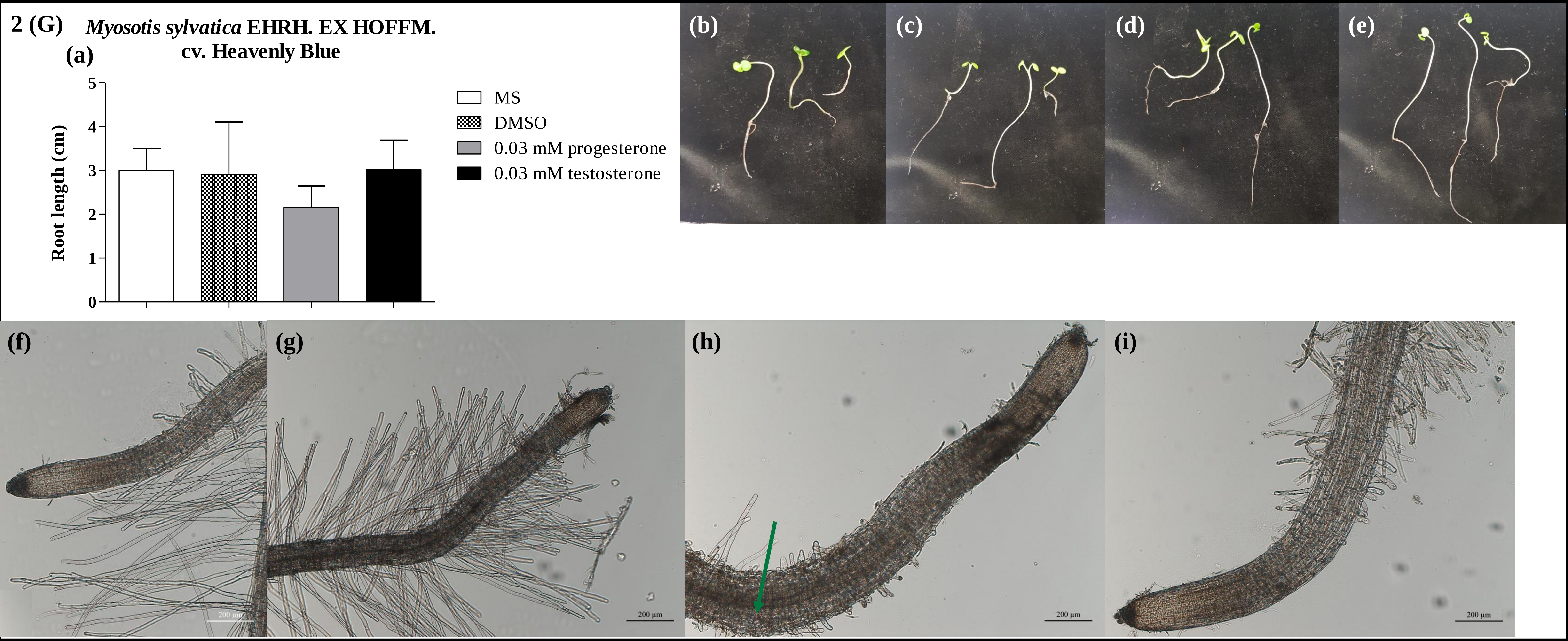
2 (G)
Myosotis sylvatica EHRH. EX HOFFM.
cv. Heavenly Blue
(a)
Root length (cm)
0
1
2
3
4
5
MS
DMSO
0.03 mM progesterone
0.03 mM testosterone
(b)
(c)
(d)
(e)
(f)
(g)
(h)
(i)
200 µm
200 µm
200 µm
200 µm

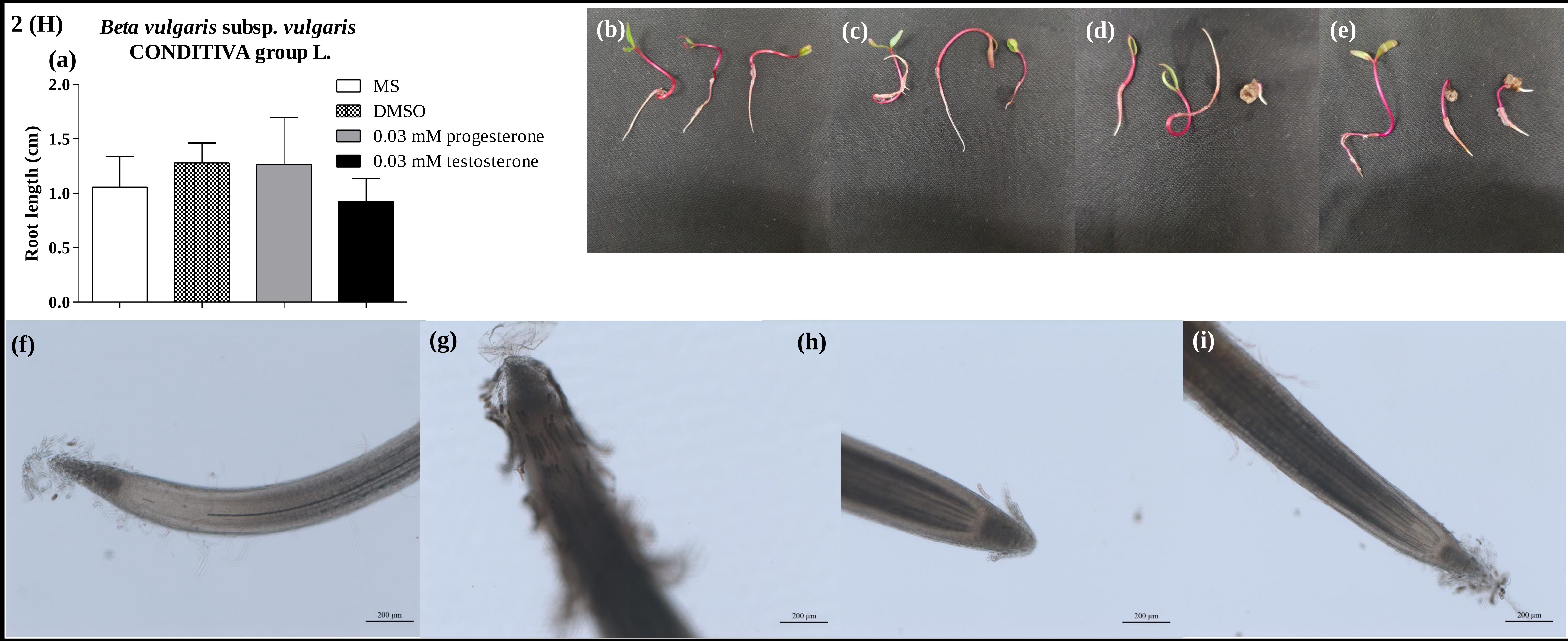

2 (H)
Beta vulgaris subsp. vulgaris
CONDITIVA group L.
(a)
Root length (cm)
2.0
1.5
1.0
0.5
0.0
MS
DMSO
0.03 mM progesterone
0.03 mM testosterone
(b)
(c)
(d)
(e)
(f)
(g)
(h)
(i)
200 µm
200 µm
200 µm
200 µm

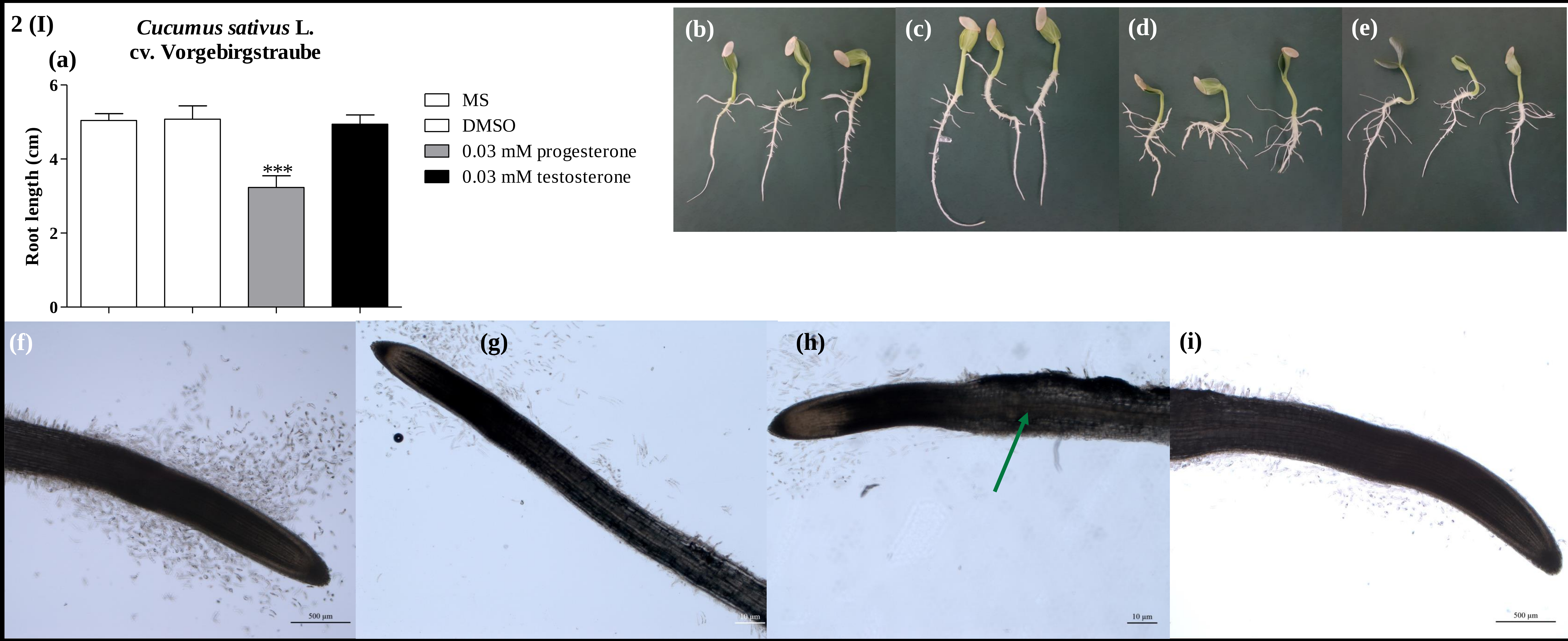
2 (I)
Cucumus sativus L.
cv. Vorgebirgstraube
(a)
Root length (cm)
6
4
2
0
***
MS
DMSO
0.03 mM progesterone
0.03 mM testosterone
(b)
(c)
(d)
(e)
(f)
(g)
(h)
(i)
500 µm
10 µm
10 µm
500 µm

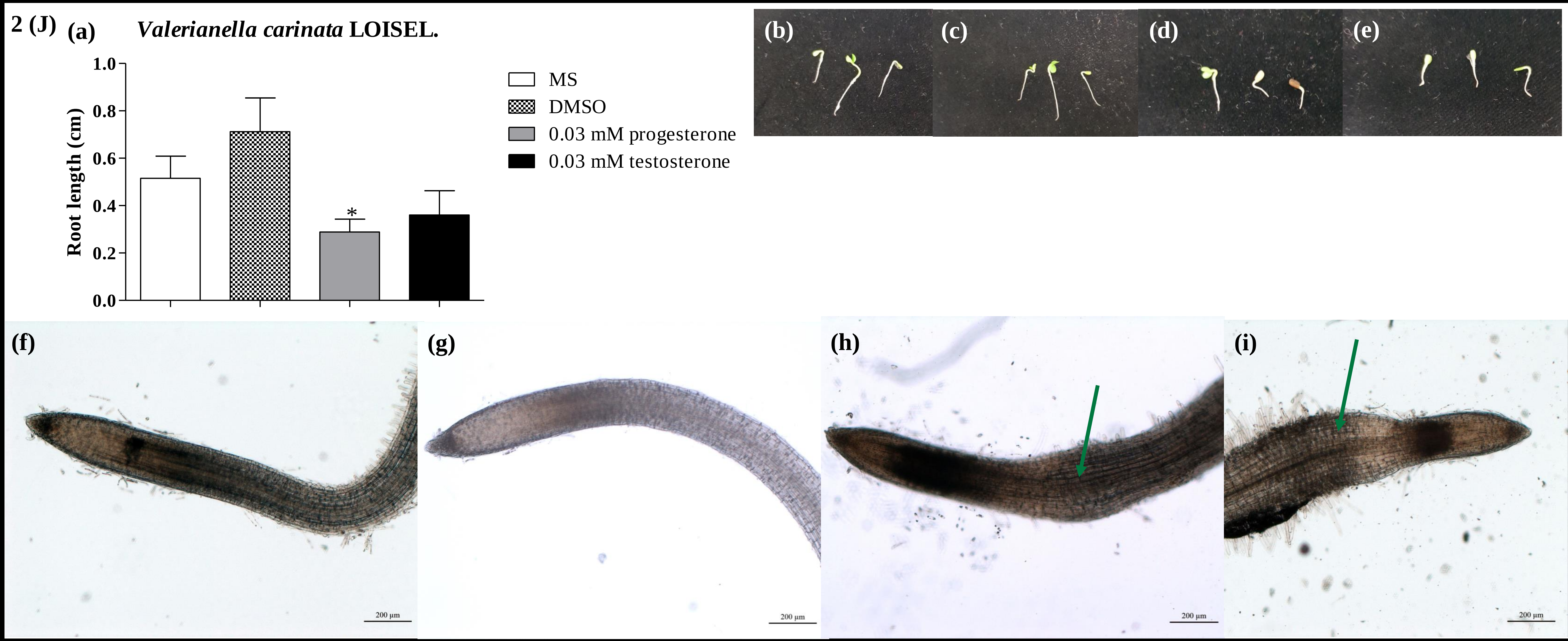

2 (J)
(a)
Valerianella carinata LOISEL.
Root length (cm)
1.0
0.8
0.6
0.4
0.2
0.0
*
MS
DMSO
0.03 mM progesterone
0.03 mM testosterone
(b)
(c)
(d)
(e)
(f)
(g)
(h)
(i)
200 µm
200 µm
200 µm
200 µm

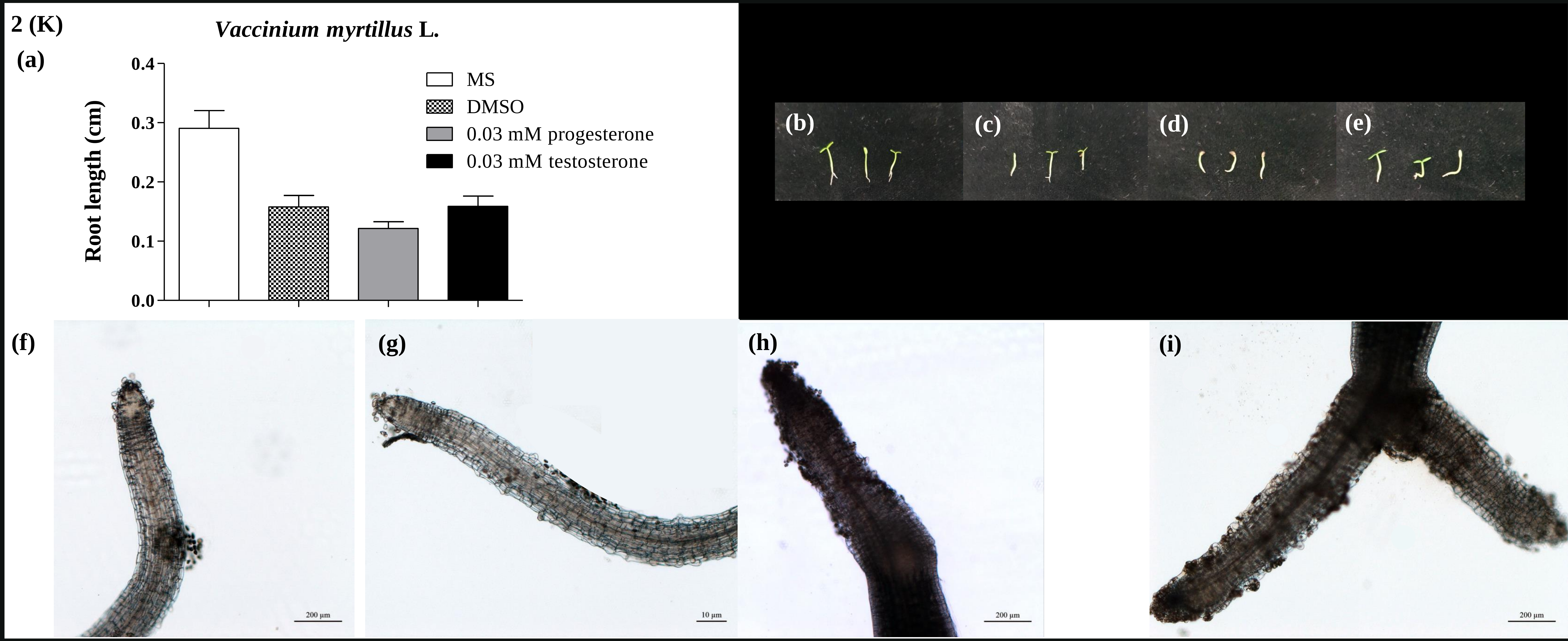

2 (K)
Vaccinium myrtillus L.
(a)
Root length (cm)
0.0
0.1
0.2
0.3
0.4
MS
DMSO
0.03 mM progesterone
0.03 mM testosterone
(b)
(c)
(d)
(e)
(f)
(g)
(h)
(i)
200 µm
10 µm
200 µm
200 µm

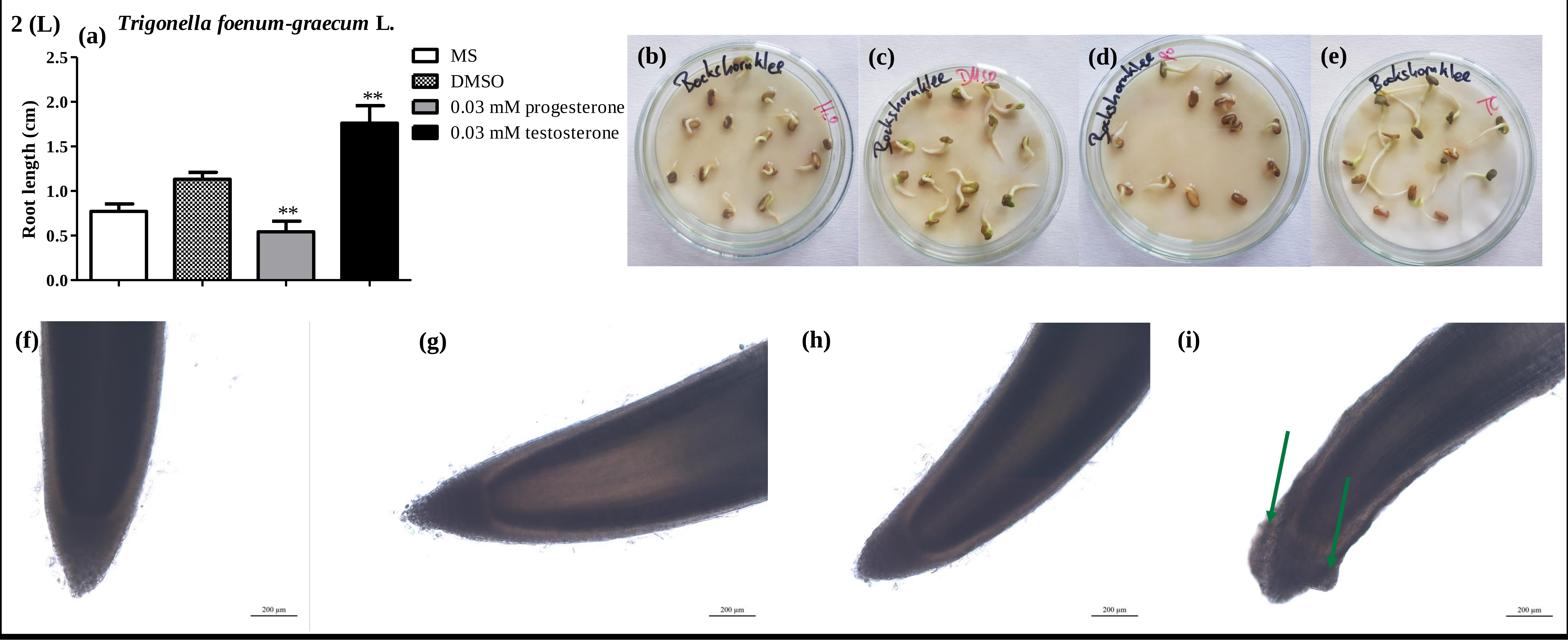

2 (L)
(a)
Trigonella foenum-graecum L.
Root length (cm)
2.5
2.0
1.5
1.0
0.5
0.0
**
**
MS
DMSO
0.03 mM progesterone
0.03 mM testosterone
(b)
(c)
(d)
(e)
(f)
(g)
(h)
(i)
200 µm
200 µm
200 µm
200 µm

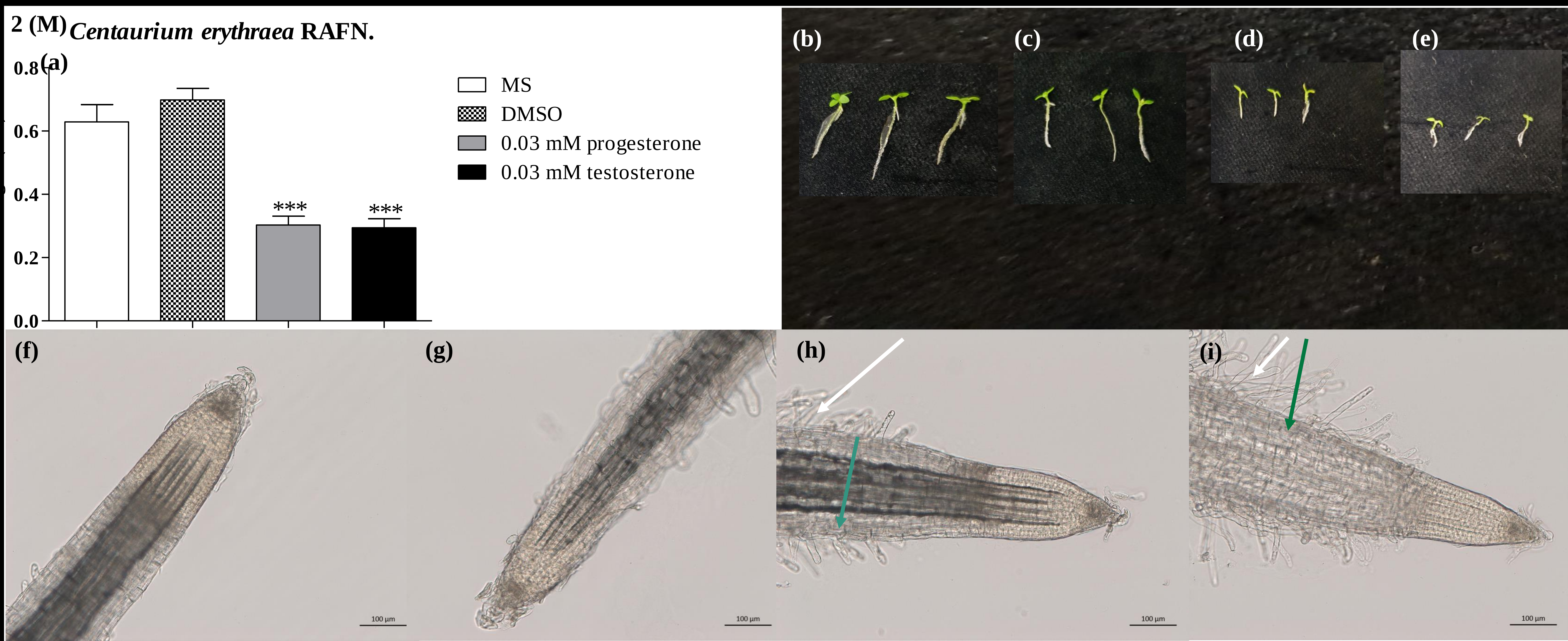

2 (M) Centaurium erythraea RAFN.
(a)
0.8
0.6
0.4
0.2
0.0
***
***
MS
DMSO
0.03 mM progesterone
0.03 mM testosterone
(b)
(c)
(d)
(e)
(f)
(g)
(h)
(i)
100 µm
100 µm
100 µm
100 µm

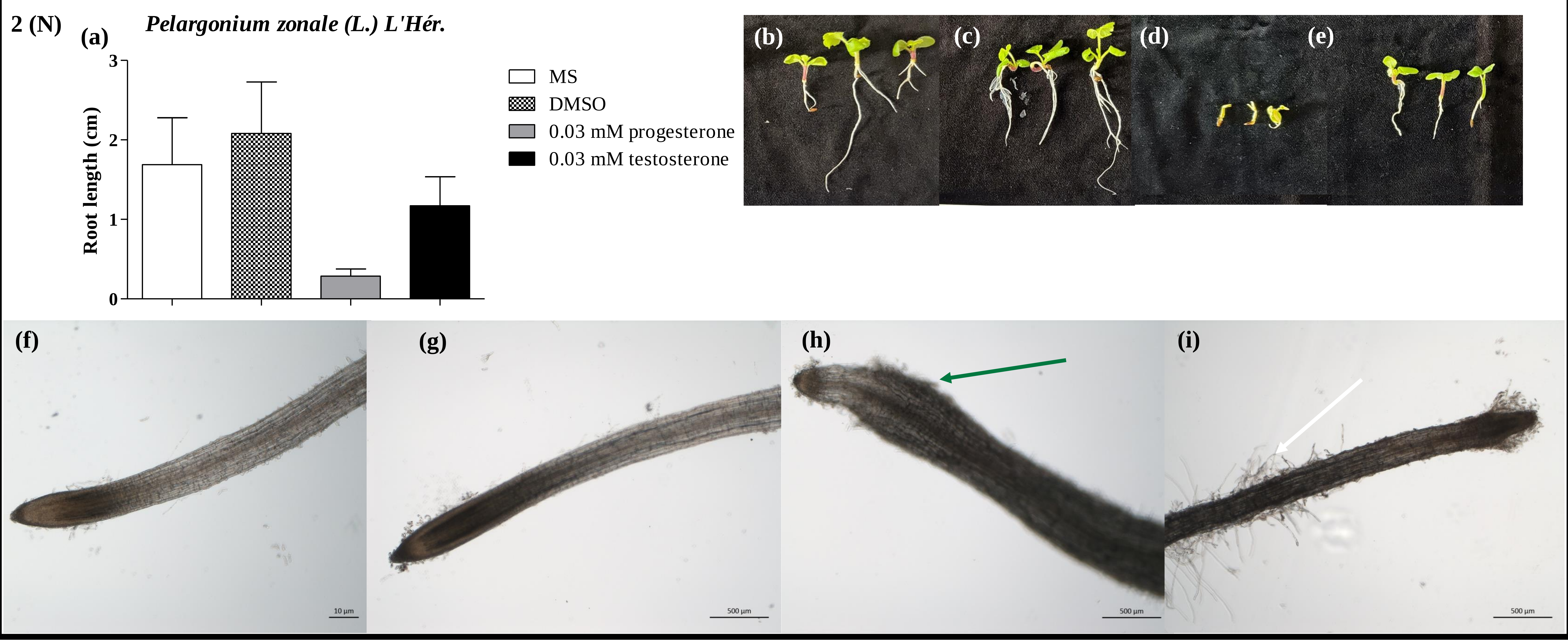
2 (N)
(a)
Pelargonium zonale (L.) L'Hér.
Root length (cm)
0
1
2
3
MS
DMSO
0.03 mM progesterone
0.03 mM testosterone
(b)
(c)
(d)
(e)
(f)
(g)
(h)
(i)
10 µm
500 µm
500 µm
500 µm

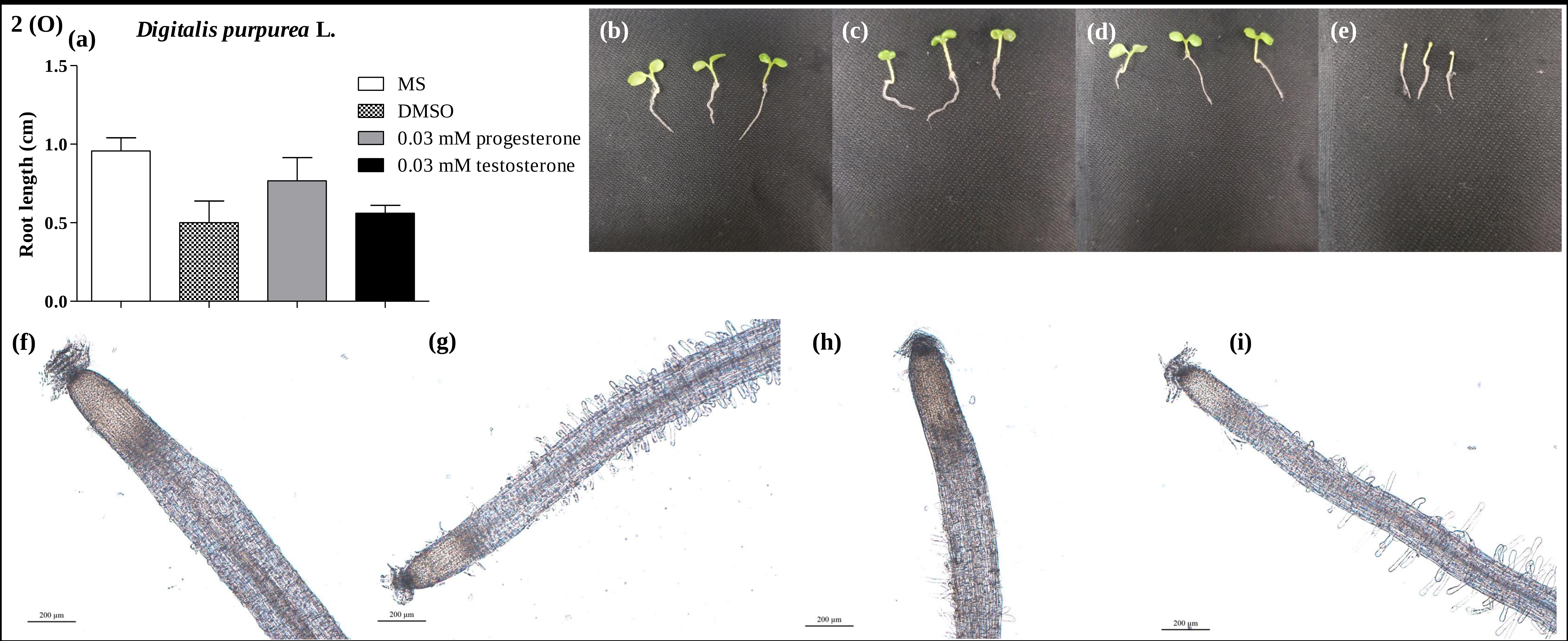
2 (O)
(a)
Digitalis purpurea L.
Root length (cm)
1.5
1.0
0.5
0.0
MS
DMSO
0.03 mM progesterone
0.03 mM testosterone
(b)
(c)
(d)
(e)
(f)
(g)
(h)
(i)
200 µm
200 µm
200 µm
200 µm

2 (P)

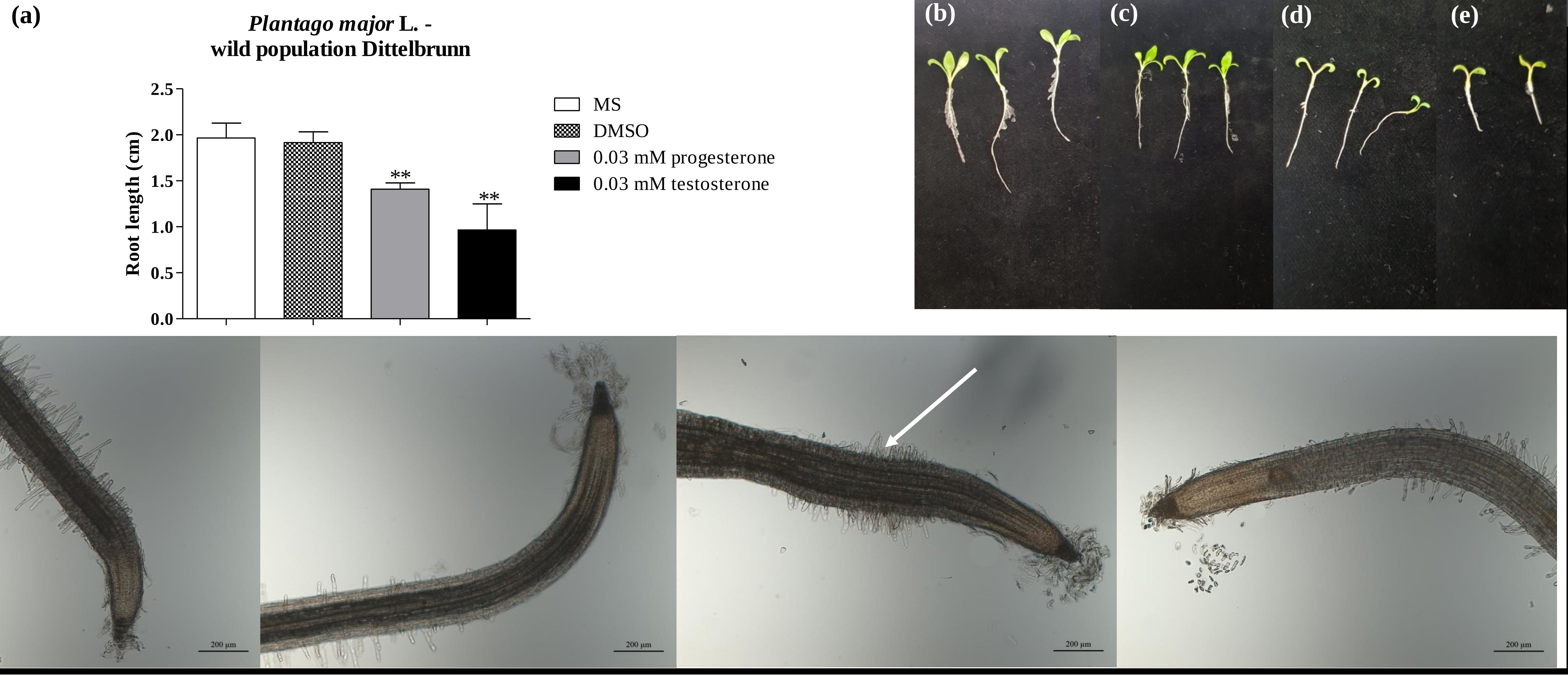

(a)
Plantago major L. -
wild population Dittelbrunn
Root length (cm)
2.5
2.0
1.5
1.0
0.5
0.0
**
**
MS
DMSO
0.03 mM progesterone
0.03 mM testosterone
(b)
(c)
(d)
(e)
200 µm
200 µm
200 µm
200 µm

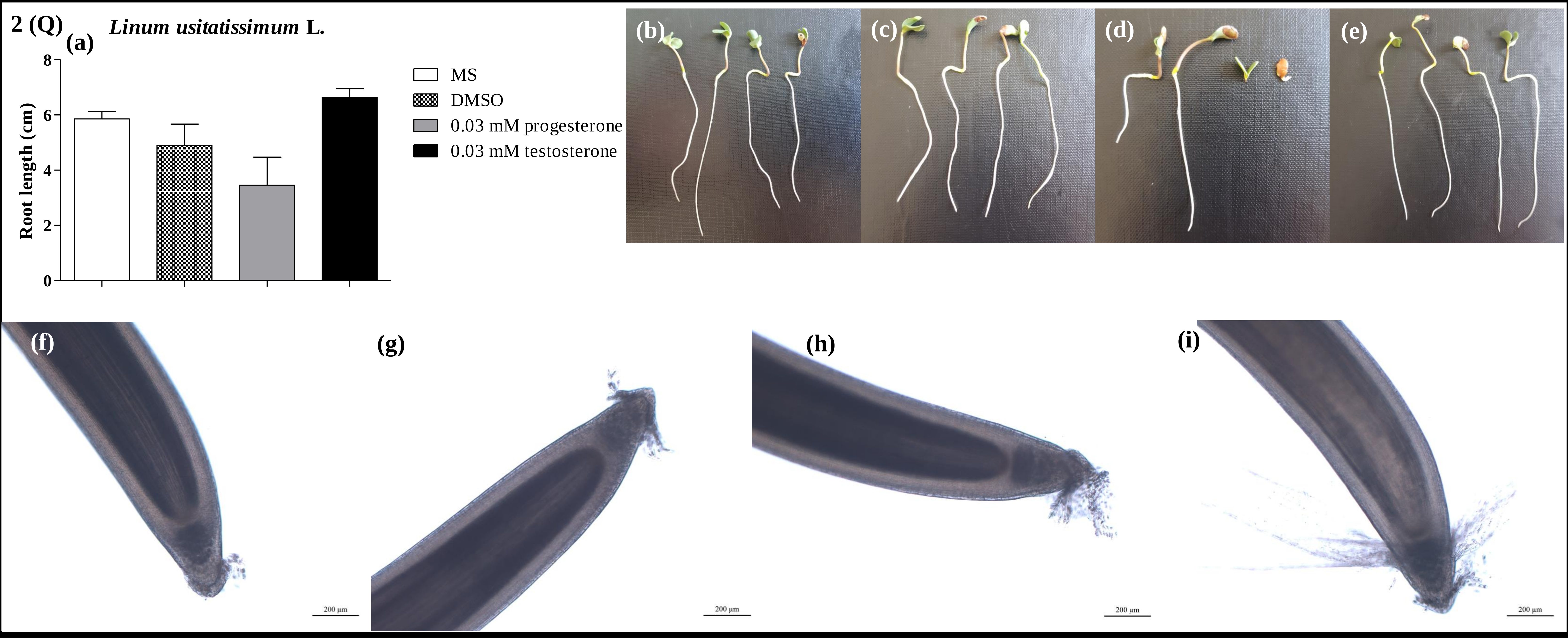
2 (Q)
(a)
Linum usitatissimum L.
Root length (cm)
0
2
4
6
8
MS
DMSO
0.03 mM progesterone
0.03 mM testosterone
(b)
(c)
(d)
(e)
(f)
(g)
(h)
(i)
200 µm
200 µm
200 µm
200 µm

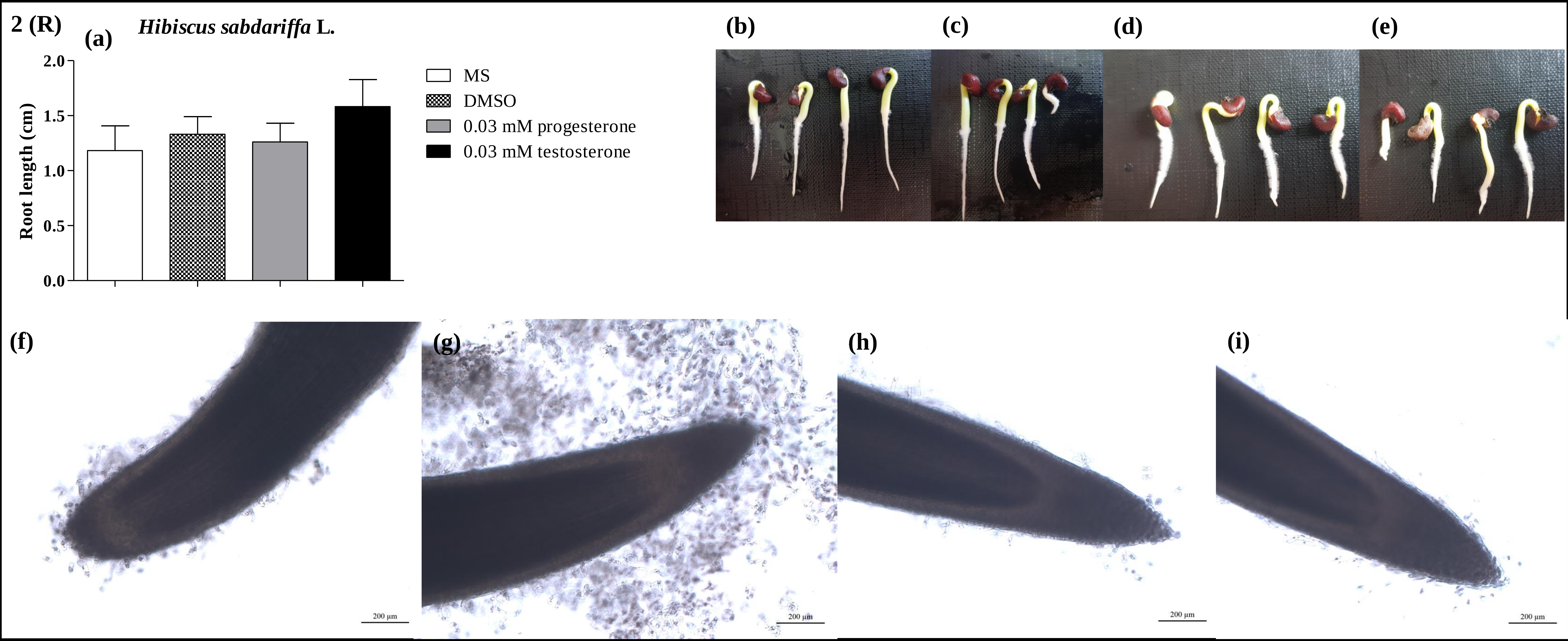
2 (R)
(a)
Hibiscus sabdariffa L.
Root length (cm)
2.0
1.5
1.0
0.5
0.0
MS
DMSO
0.03 mM progesterone
0.03 mM testosterone
(b)
(c)
(d)
(e)
(f)
(g)
(h)
(i)
200 µm
200 µm
200 µm
200 µm

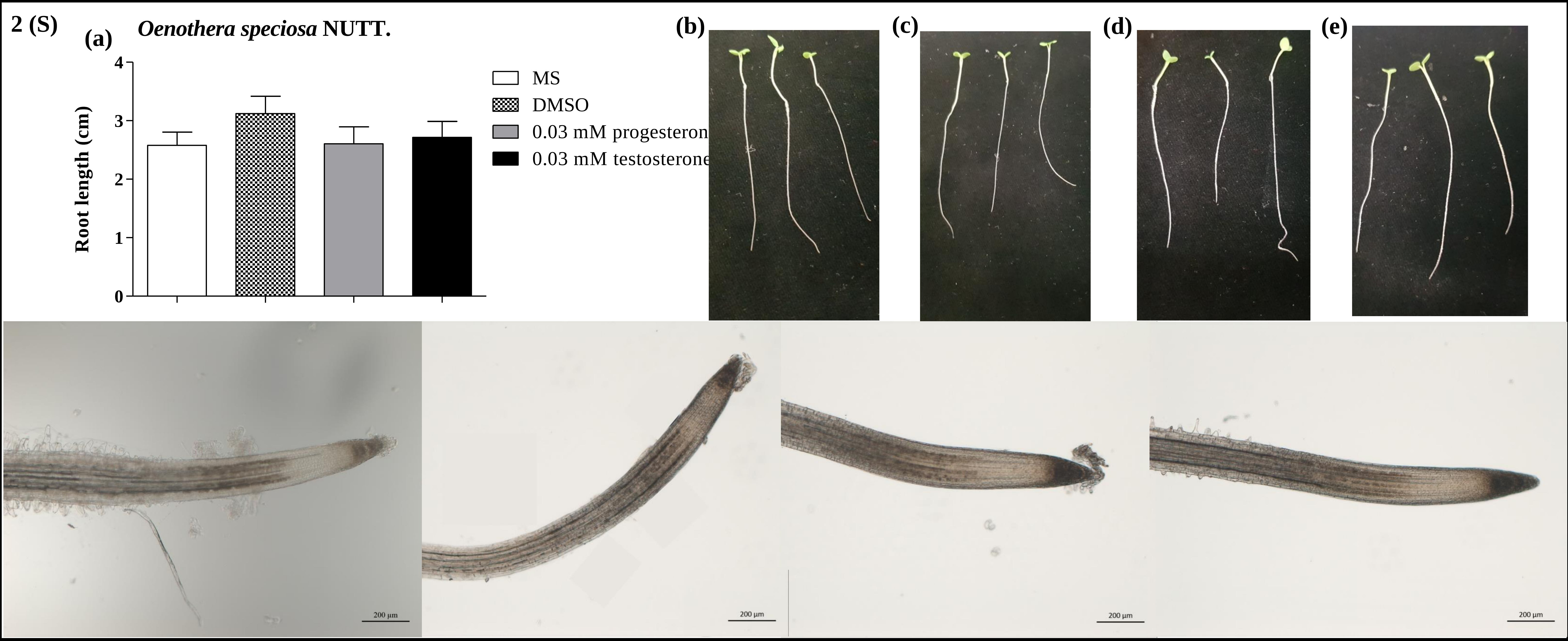
2 (S)
(a)
Oenothera speciosa NUTT.
Root length (cm)
0
1
2
3
4
MS
DMSO
0.03 mM progesteron
0.03 mM testosterone
(b)
(c)
(d)
(e)
200 µm
200 µm
200 µm
200 µm

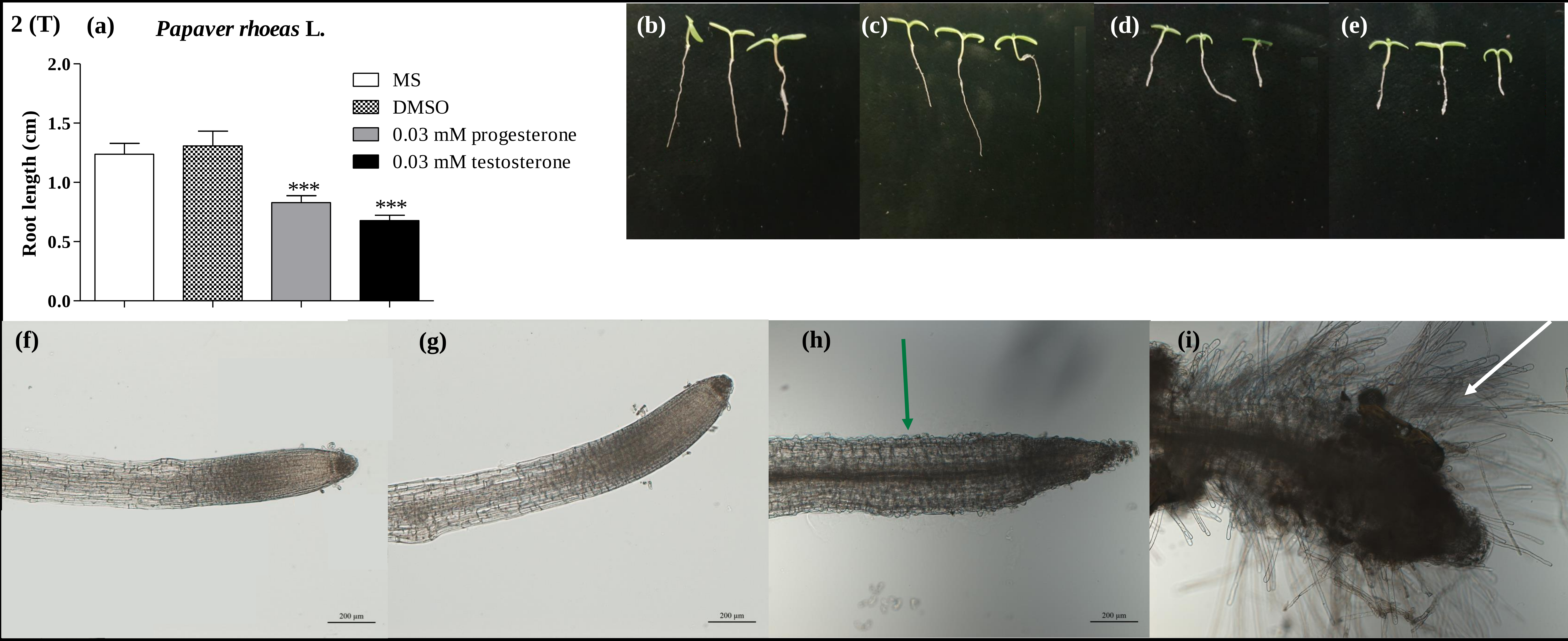
2 (T)
(a)
Papaver rhoeas L.
Root length (cm)
2.0
1.5
1.0
0.5
0.0
MS
DMSO
0.03 mM progesterone
0.03 mM testosterone
***
***
(b)
(c)
(d)
(e)
(f)
(g)
(h)
(i)
200 µm
200 µm
200 µm

2 (U)

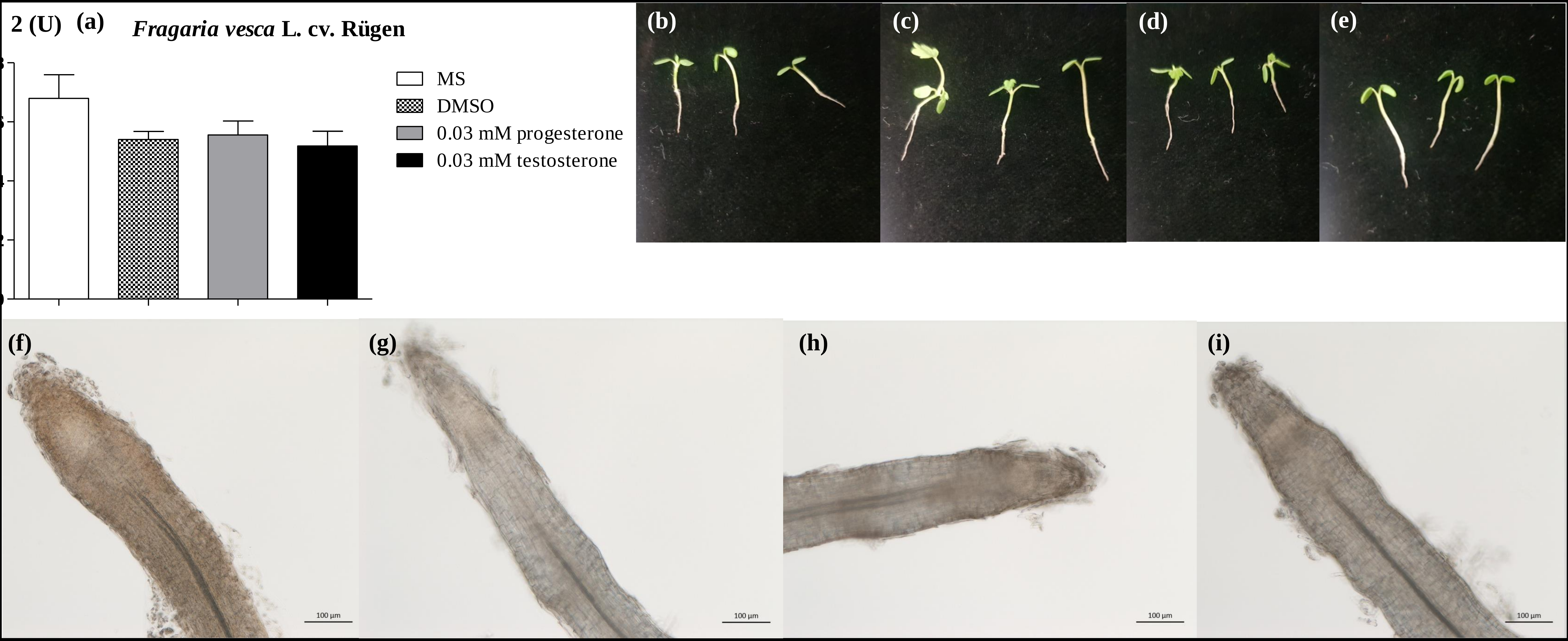

(a)
Fragaria vesca L. cv. Rügen
MS
DMSO
0.03 mM progesterone
0.03 mM testosterone
(b)
(c)
(d)
(e)
(f)
(g)
(h)
(i)
100 µm
100 µm
100 µm
100 µm

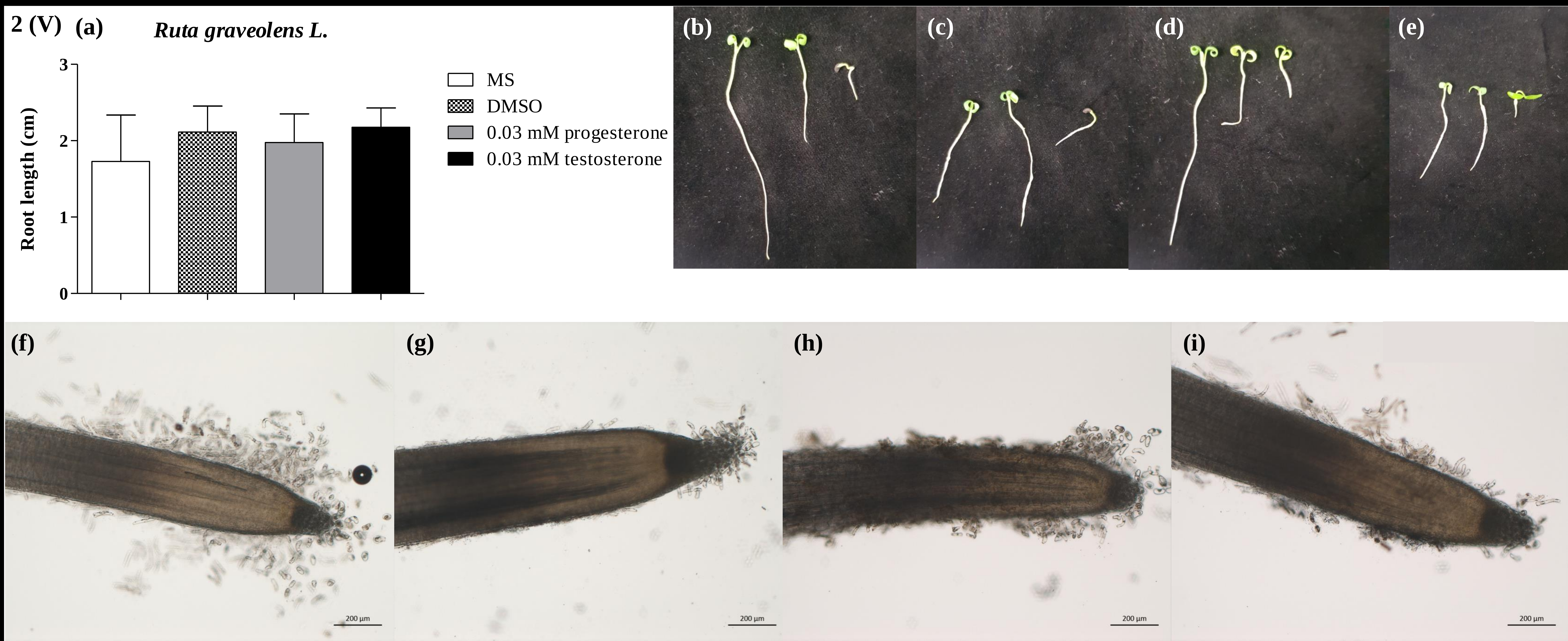
2 (V)
(a)
Ruta graveolens L.
Root length (cm)
3
2
1
0
MS
DMSO
0.03 mM progesterone
0.03 mM testosterone
(b)
(c)
(d)
(e)
(f)
(g)
(h)
(i)
200 µm
200 µm
200 µm
200 µm

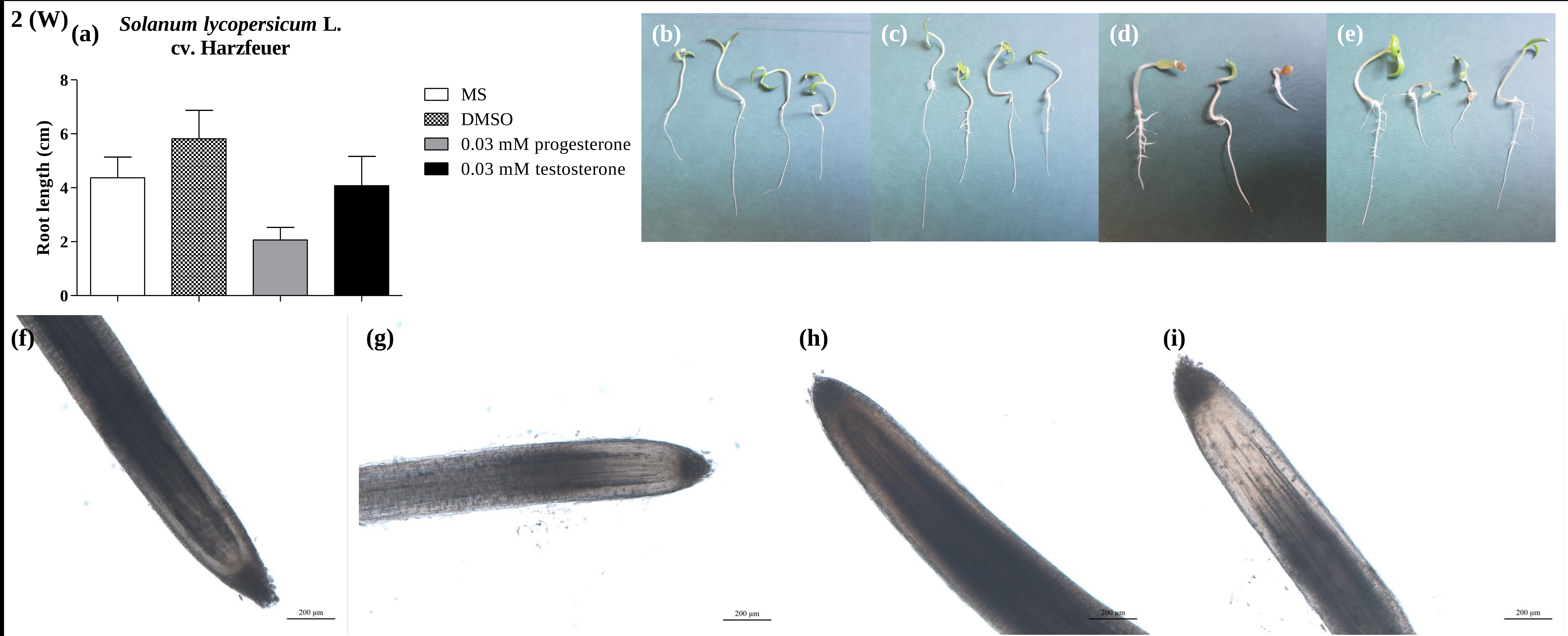
2 (W)
(a)
Solanum lycopersicum L.
cv. Harzfeuer
Root length (cm)
0
2
4
6
8
MS
DMSO
0.03 mM progesterone
0.03 mM testosterone
(b)
(c)
(d)
(e)
(f)
(g)
(h)
(i)
200 µm
200 µm
200 µm
200 µm

Supporting information Figure S3: Seedling and root morphology of progesterone- or testosterone-treated non-Brassicales angiosperms. The figure depicts the morphology of seedlings and roots of the following *Brassicacea* species: (**A**) *Nymphaea colorata* PETER., (**B**) Spirodela polyrhiza (L.) SCHLEID., (**C**) *Allium schoenoprasum* L. cv. Nelly, (**D**) *Secale cereal* L. cv. Dukato, (**E**) *Petroselinum crispum* (MILL.) FUSS cv. Mooskrause, (**F**) *Taraxacum officinale* L. – wild population Dittelbrunn, (**G**) *Myosotis sylvatica* EHRH. EX HOFFM. cv. Heavenly Blue, (**H**) *Beta vulgaris* subsp. *vulgaris* CONDITIVA group L., (**I**) *Cucumis sativus* L. cv. Vorgebirgstraube, (**J**) *Valerianella carinata* LOISEL., (**K**) *Vaccinium myrtillus* L., (**L**) *Trigonella foenum-graecum* L., (**M**) *Centaurium erythraea* RAFN., **(N)** *Pelargonium zonale* (L.) L'Hér., **(O)** *Digitalis purpurea* L., (**P**) *Plantago major* L. – wild population Dittelbrunn, (**Q**) *Linum usitatissimum* L., (**R**) *Hibiscus sabdariffa* L., (**S**) *Oenothera speciosa* NUTT., (**T**) *Papaver rhoeas* L., (**U**) *Fragaria vesca* L. cv. Rügen, (**V**) *Ruta graveolens* L. (**W**) *Solanum lycopersicum* L. cv. Harzfeuer (**a**) gives the root lengths of the analyzed plant as mean ± SEM. Statistical differences, indicated by asterisks (* = $p \leq 0.05$; ** = $p \leq 0.01$; *** = $p \leq 0.001$), were determined by one-way ANOVA and Turkey test. (**b – e**) are pictures of the morphology of the seedlings. (**f – h**) show microscopic pictures of the root tips of the analyzed plant. b and f = MS control; c and g = DMSO mock treatment; d and h = 30 µM progesterone; e and h = 30 µM testosterone. Green arrows indicate uncoordinated cell growth, while white arrows indicate enhanced root hair development.
