# Supplementary material for: Progestogens and androgens influence root morphology of angiosperms in a brassinosteroid‐independent manner
Source: Plant J. 2025 Sep 9;123(5):e70459. doi: 10.1111/tpj.70459 (PMC12419790; doi:10.1111/tpj.70459)
Supplement: Supplementary file 4 — Figure S4. Cloning and overexpression of AtDET2 in A. thaliana. (A) We here show the used cloning strategy to obtain a construct for AtDET2 overexpression. The original insert of pFAU27 was removed by digestion with XbaI and SalI (highlighted in green). These restriction enzymes do not cut the coding sequence of AtDET2. AtDET2 amplificates with XbaI and SalI restriction sites were designed by PCR and inserted into the digested pFAU27. (B) Seeds of floral dip A. thaliana plants were selected using kanamycin. Plants surviving on kanamycin were analysed by PCR against nptII (the T‐DNA located resistance genes). We here show the PCR against nptII for several transgenic plants. (C) The intensity of DET2 expression in T‐DNA containing A. thaliana plants was tested by qPCR. As expected, DET2 expression intensity shows a strong variety between the transgenic plants. Lines 1 and 3 show a tremendous upregulation (factor 200 compared with the wild type), while it is still very strong for line 2 (factor 11). That is why we used these lines for following experiments. [file TPJ-123-0-s011.pdf]

(A)

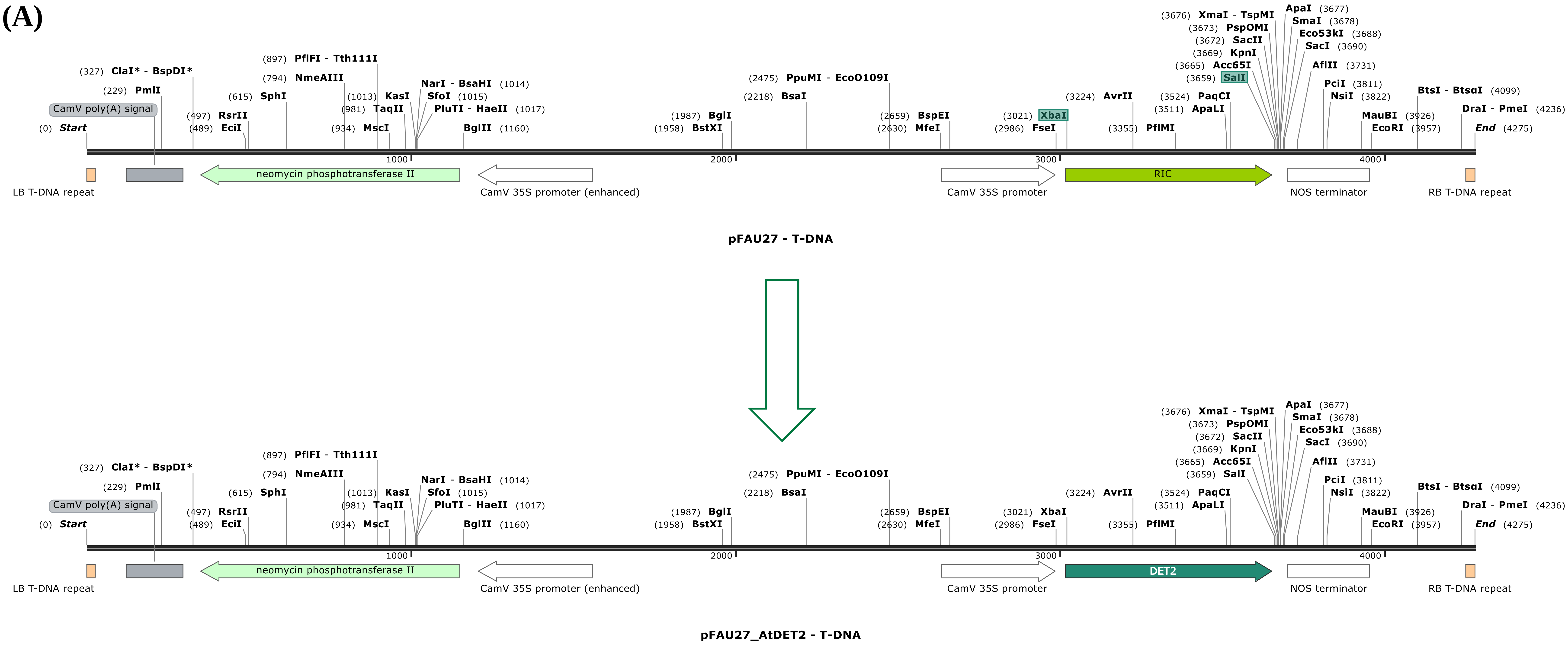

(0) Start
(229) PmlI
(327) ClaI* - BspDI*
(489) EciI
(497) RsrII
(615) SphI
(794) NmeAIII
(897) PflFI - Tth111I
(934) MscI
(981) TaqII
(1013) KasI
NarI - BsaHI (1014)
SfoI (1015)
PluTI - HaeII (1017)
BglII (1160)
(1958) BstXI
(1987) BglI
(2218) BsaI
(2475) PpuMI - EcoO109I
(2630) MfeI
(2659) BspEI
(2986) FseI
(3021) XbaI
(3224) AvrII
(3355) PflMI
(3511) ApaLI
(3524) PaqCI
(3659) SalI
(3665) Acc65I
(3669) KpnI
(3672) SacII
(3673) PspOMI
(3676) XmaI - TspMI
ApaI (3677)
SmaI (3678)
Eco53kI (3688)
SacI (3690)
AflII (3731)
PciI (3811)
NsiI (3822)
MauBI (3926)
EcoRI (3957)
BtsI - BtsαI (4099)
DraI - PmeI (4236)
End (4275)
CamV poly(A) signal
1000
2000
3000
4000
LB T-DNA repeat
neomycin phosphotransferase II
CamV 35S promoter (enhanced)
CamV 35S promoter
RIC
NOS terminator
RB T-DNA repeat
pFAU27 - T-DNA
DET2
pFAU27_AtDET2 - T-DNA

(B)

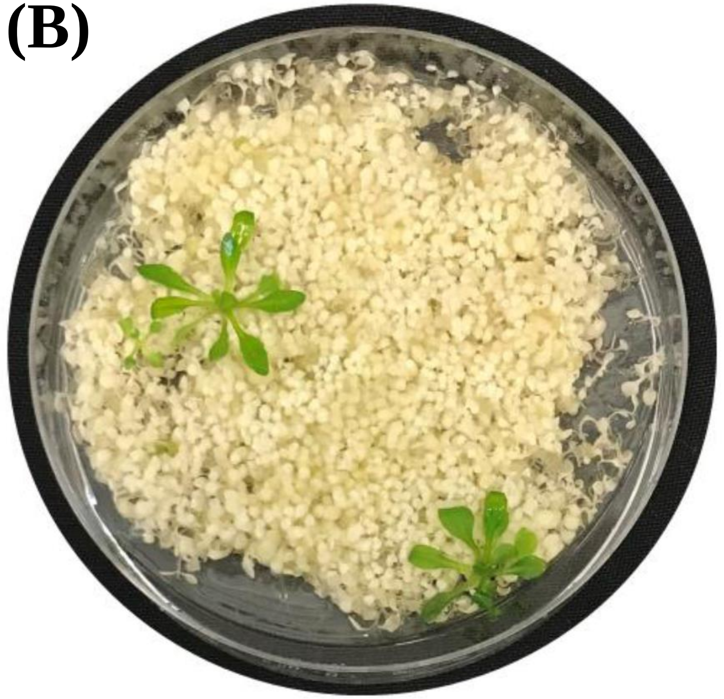

(D)

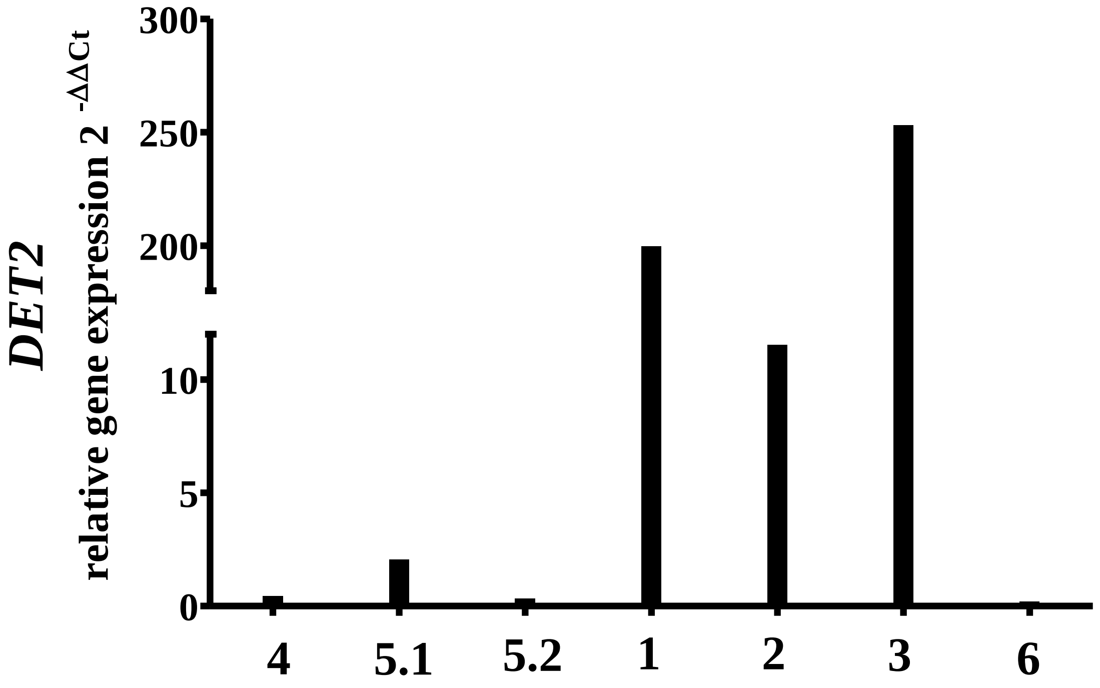
DET2
relative gene expression 2 -ΔΔCt
300
250
200
10
5
0
4
5.1
5.2
1
2
3
6

(C)

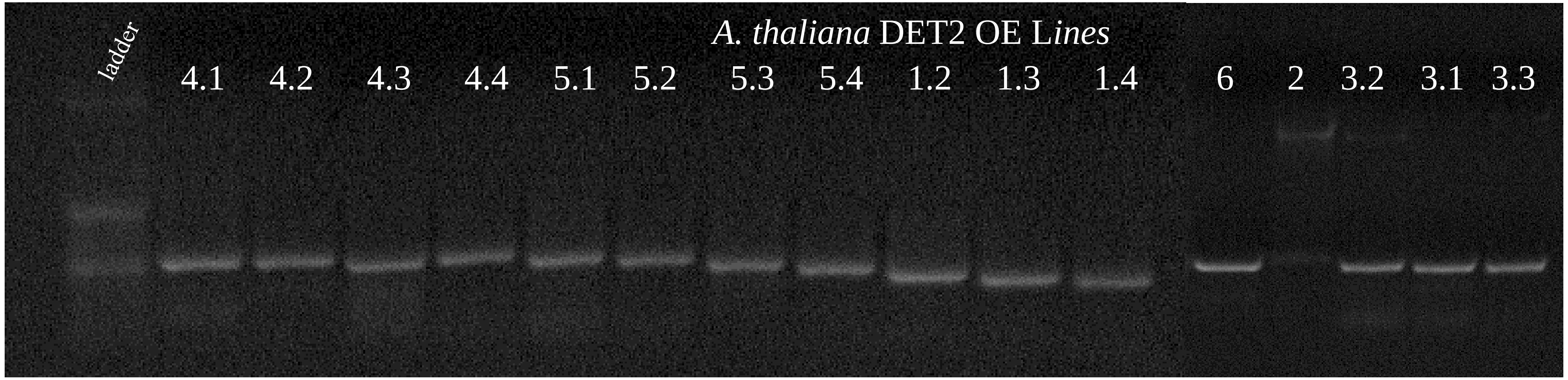
ladder
A. thaliana DET2 OE Lines
4.1
4.2
4.3
4.4
5.1
5.2
5.3
5.4
1.2
1.3
1.4
6
2
3.2
3.1
3.3

SI Figure S4: Cloning and overexpression of AtDET2 in *A. thaliana*. (A) We here show the used cloning strategy to obtain a construct for AtDET2 overexpression. The original insert of pFAU27 was removed by digestion with XbaI and SalI (highlighted in green). These restriction enzymes do not cut the coding sequence of AtDET2. AtDET2 amplificates with XbaI and SalI restriction sites were designed by PCR and inserted into the digested pFAU27. (B) Seeds of floral dip *A. thaliana* plants were selected using kanamycin. Plants surviving on kanamycin, were analysed by PCR against nptII (the T-DNA located resistance genes). We here show the PCR against nptII for several transgenic plants. (C) The intensity of DET2 expression in T-DNA containing *A. thaliana* plants was tested by qPCR. As expected DET2 expression intensity shows a strong variety between the transgenic plants. Lines 1 and 3 show a tremendous upregulation (factor 200 compared to the wildtype), while it is still very strong for line 2 (factor 11). That is why we used these lines for following experiments.
