# Supplementary material for: Progestogens and androgens influence root morphology of angiosperms in a brassinosteroid‐independent manner
Source: Plant J. 2025 Sep 9;123(5):e70459. doi: 10.1111/tpj.70459 (PMC12419790; doi:10.1111/tpj.70459)
Supplement: Supplementary file 5 — Figure S5. The effects of progesterone and testosterone on loss‐of‐function mutants in brassinosteroid signalling. (A) We here show a simplified graphic of brassinosteroid signalling following Kim and Russinova (2020). The brassinosteroid receptor Brassinosteroid‐Insensitive1 (BRI1) remains, in the absence of brassinosteroids, inactive due to the autoinhibitory C‐terminus and its association with BRI1 Kinase Inhibitor1 (BKI1). As a consequence, Brassinosteroid‐Insensitive2 (BIN2) is constitutively active and phosphorylates the transcription factors BRI1‐EMS Suppressor1 (BES1)/Brassinazole‐Resistant1 (BZR1). This phosphorylation promotes their 14‐3‐3‐mediated degradation within the cytosol and thereby inhibits their DNA‐binding activities. In the presence of brassinosteroids, the receptor kinases BRI1 and BAK1 are activated. This activation leads to a dissociation of BKI1 from BRI1, as well as the phosphorylation and activation of BR‐Signalling Kinases (BSKs)/Constitutive Differential Growth (CDGs) and BRI1 Suppressor1 (BSU1). After activation, BSU1 dephosphorylates and inactivates BIN2, which will be degraded. These events result in the accumulation of Protein Phosphatase 2A (PP2A) within the nucleus. PP2A dephosphorylates BES1/BZR1, which results in the binding of BES1/BZR1 to Brassinosteroid Response Element (BRRE)/E‐box‐containing promoters. This binding regulates the expression of numerous BR‐responsive genes crucial for plant growth and development. (B and C) We analysed the effects of progesterone and testosterone on the root length in loss‐of‐function lines of BR1 (bri1), as well as BSK1 (bsk1). Both lines showed the typical dwarf phenotype, caused by inhibited brassinosteroid signalling. When germinated on progesterone‐ or testosterone‐containing medium, these lines showed an additional, and statistically significant, reduction in root length. The graphs give mean ± SEM. Statistical differences, indicated by asterisks (*P ≤ 0.05; **P ≤ 0.01; ***P ≤ 0.001), w [file TPJ-123-0-s013.pdf]

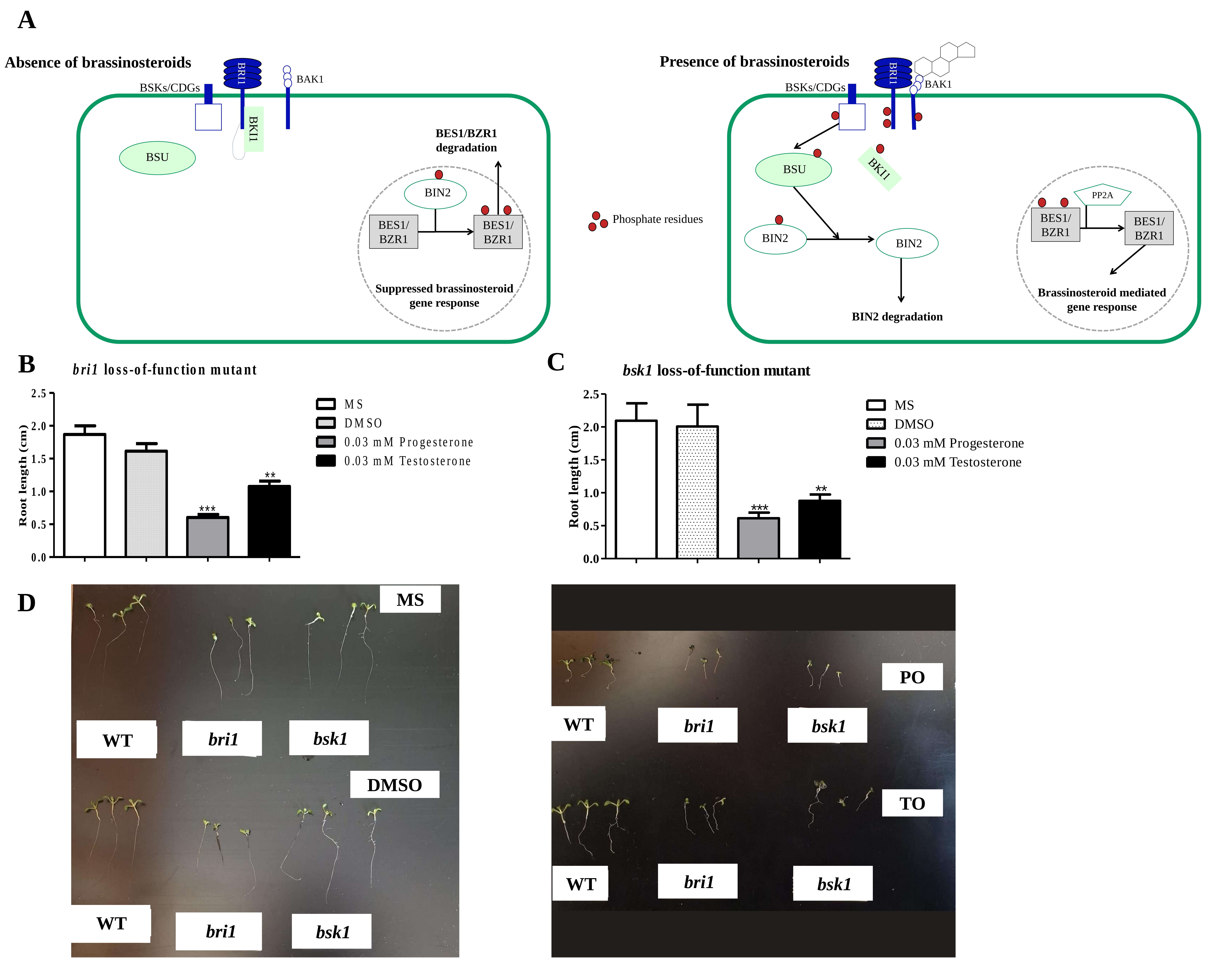
A
Absence of brassinosteroids
BSKs/CDGs
BRI1
BAK1
BKI1
BSU
BES1/BZR1
degradation
BIN2
BES1/
BZR1
BES1/
BZR1
Suppressed brassinosteroid
gene response
Phosphate residues
Presence of brassinosteroids
BSKs/CDGs
BRI1
BAK1
BSU
BKI1
BIN2
BIN2
BIN2 degradation
PP2A
BES1/
BZR1
BES1/
BZR1
Brassinosteroid mediated
gene response
B
bri1 loss-of-function mutant
Root length (cm)
2.5
2.0
1.5
1.0
0.5
0.0
***
**
MS
DMSO
0.03 mM Progesterone
0.03 mM Testosterone
C
bsk1 loss-of-function mutant
Root length (cm)
2.5
2.0
1.5
1.0
0.5
0.0
***
**
MS
DMSO
0.03 mM Progesterone
0.03 mM Testosterone
D
MS
WT
bri1
bsk1
DMSO
WT
bri1
bsk1
PO
WT
bri1
bsk1
TO
WT
bri1
bsk1

SI Figure S5: The effects of progesterone and testosterone on loss-of-function mutants in brassinosteroid signalling. (A) We here show a simplified graphic of brassinosteroid signalling following Kim and Russinova (2020). The brassinosteroid receptor Brassinosteroid-Insensitive1 (BRI1) remains, in the absence of brassinosteroids, inactive due to the autoinhibitory C-terminus and its association with BRI1 Kinase Inhibitor1 (BKI1). As a consequence, the Brassinosteroid-Insensitive2 (BIN2) is constitutively active and phosphorylates the transcription factors BRI1-EMS Suppressor1 (BES1)/Brassinazole-Resistant1 (BZR1). This phosphorylation promotes their 14-3-3-mediated degradation within the cytosol, and thereby inhibits their DNA-binding activities. In the presence of brassinosteroids, the receptor kinases BRI1 and BAK1 are activated. This activation leads to a dissociation of BKI1 from BRI1, as well as the phosphorylation and activation of BR-Signalling Kinases (BSKs)/Constitutive Differential Growth (CDGs) and BRI1 Suppressor1 (BSU1). After activation, BSU1 dephosphorylates and inactivates BIN2, which will be degraded. These events result in the accumulation of the Protein Phosphatase 2A (PP2A) within the nucleus. PP2A dephosphorylates BES1/BZR1, which results in binding of BES1/BZR1 to Brassinosteroid Response Element (BRRE)/E-box-containing promoters. This binding regulates the expression of numerous BR-responsive genes crucial for plant growth and development. **(B and C)** We analysed the effects of progesterone and testosterone on the root length in loss-of-function lines of BR1 (*bri1*), as well as BSK1 (*bsk1*). Both lines showed the typical dwarf phenotype, caused by inhibited brassinosteroid signalling. When germinated on progesterone- or testosterone-containing medium, these lines showed an additional, and statistically significant, reduction in root length. The graphs give mean ± SEM. Statistical differences, indicated by asterisks (* = $p \leq 0.05$; ** = $p \leq 0.01$; *** = $p \leq 0.001$), were determined by one-way ANOVA and the Turkey test. (D) Phenotypes of treated *bsk1* and *bri1* lines, as well as wild type (WT) were documented for progesterone-treated, testosterone-treated, mock-treated, and untreated plants.

Kim, E.-J. and Russinova, E. (2020) Brassinosteroid signalling. *Current biology : CB*, 30, R294-R298.
