# Supplementary material for: Progestogens and androgens influence root morphology of angiosperms in a brassinosteroid‐independent manner
Source: Plant J. 2025 Sep 9;123(5):e70459. doi: 10.1111/tpj.70459 (PMC12419790; doi:10.1111/tpj.70459)
Supplement: Supplementary file 6 — Table S1. Details of analysis of steroids by LC–MS/MS. A volume of 2 μL was injected into an Agilent 1260 Infinity II LC system, consisting of a binary pump G7112B, an autosampler G7167A and a column thermostat G7116A (Agilent Technologies, Santa Clara, CA, USA) without preconcentration or filtering. Chromatographic separation was carried out on a ZORBAX Eclipse XDB‐C18 column (50 × 4.6 mm, 1.8 μm) from Agilent Technologies (Santa Clara, CA, USA). A binary solvent system was used as mobile phase consisting of (A) 0.05% formic acid in water and (B) acetonitrile with a constant flow rate of 1.1 mL/min at 20°C column temperature. The following gradient was applied: 0.00–0.50 min, 60% A; 0.50–5.00 min, 60% to 10% A; 5.00–5.05 min, 10% to 0% A; 5.05–6.50 min, 0% A; 6.50–6.55 min, 0%–60 % A; 6.55–9.00 min, 60% A. The column outlet was connected to a QTRAP 6500+ triple quadrupole mass spectrometer (AB Sciex LLC, Framingham, MA, USA). The Turbo Spray IonDrive ion source was running in positive ionisation mode with 5500 V ion spray voltage and 650 °C turbo gas temperature. The curtain gas was set to 40 psi; the collision gas to ‘medium’ and both ion source gases 1 and 2 were set to 70 psi. Scheduled multiple reaction monitoring (scheduled MRM) was used to monitor analyte parent ion → product ion fragmentations as described in Table 1. Q1 and Q3 quadrupoles were maintained at unit resolution. Analyst 1.6 software (Applied Biosystems) was used for data acquisition and processing. Nona‐deuterated progesterone (PO‐d 9) was used as internal standard (IS) for quantification. The response factors (analyte × standard−1) of individual steroids relative to the internal standard have been experimentally determined. The table shows mass‐to‐charge ratio (m/z), retention time (RT), collision energy (CE) and the response factor to the used internal standard (f). [file TPJ-123-0-s002.pdf]

SI Table S1: Details of analysis of steroids by LC-MS/MS. A volume of 2 µL was injected into an Agilent 1260 infinity II LC system, consisting of a binary pump G7112B, an autosampler G7167A and a column thermostat G7116A (Agilent Technologies, Santa Clara, CA, USA) without preconcentration or filtering. Chromatographic separation was carried out on a ZORBAX Eclipse XDB-C18 column (50 × 4.6 mm, 1.8 µm) from Agilent Technologies (Santa Clara, CA, USA). A binary solvent system was used as mobile phase consisting of A) 0.05% formic acid in water and B) acetonitrile with a constant flow rate of 1.1 mL/min at 20 °C column temperature. The following gradient was applied: 0.00-0.50 min, 60% A; 0.50-5.00 min, 60-10% A; 5.00-5.05 min, 10-0% A; 5.05-6.50 min, 0% A; 6.50-6.55 min, 0-60% A; 6.55-9.00 min, 60% A. The column outlet was connected to a QTRAP 6500+ triple quadrupole mass spectrometer (AB Sciex LLC, Framingham, MA, USA). The Turbo Spray IonDrive ion source was running in positive ionization mode with 5500 V ion spray voltage and 650 °C turbo gas temperature. The curtain gas was set to 40 psi; the collision gas to 'medium' and both ion source gases 1 & 2 were set to 70 psi. Scheduled multiple reaction monitoring (scheduled MRM) was used to monitor analyte parent ion → product ion fragmentations as described in table 1. Q1 and Q3 quadrupoles were maintained at unit resolution. Analyst 1.6 software (Applied Biosystems) was used for data acquisition and processing. Nona-deuterated progesterone (PO-d_9) was used as internal standard (IS) for quantification. The response factors (analyte x standard^{-1}) of individual steroids relative to the internal standard have been experimentally determined. The table shows mass to charge ratio (*m/z*), retention time (RT), collision energy (CE) and the response factor to the used internal standard (*f*).

Compound	Usage:	*m/z:*	RT [min]	CE [V]	*f*
**PR**	Quantifier	299→ 281	4.29	15	0.49
	Qualifier	317→ 299		13	
**PO**	Quantifier	315→ 97	4.43	25	1.23
	Qualifier	315→ 109		30	
**DHP**	Quantifier	317→ 299	5.31	17	0.23
	Qualifier	317→ 281		19	
**17α-OHPR-**	Quantifier	315→ 297	2.80	13	0.11
	Qualifier	333→ 297		13	
**DHEA**	Quantifier	289→ 271	3.01	9	0.08
	Qualifier	289→ 253		15	
**17α-OHPO**	Quantifier	331→ 97	3.15	27	0.57
	Qualifier	331→ 109		30	
**AD**	Quantifier	287→ 211	3.07	27	0.09
	Qualifier	287→ 173		29	
**TO**	Quantifier	289→ 97	2.58	25	1.34
	Qualifier	289→ 109		30	
**DHT**	Quantifier	291→ 255	3.45	21	0.84
	Qualifier	291→ 273		19	
**PO- D_9**	Quantifier	324→ 100	4.43	29	-
