# Supplementary material for: Progestogens and androgens influence root morphology of angiosperms in a brassinosteroid‐independent manner
Source: Plant J. 2025 Sep 9;123(5):e70459. doi: 10.1111/tpj.70459 (PMC12419790; doi:10.1111/tpj.70459)
Supplement: Supplementary file 7 — Table S2. Sequences of steroidogenesis from plants without available genomes. Transcripts from the following NCBI bioprojects were analysed for species without available genome sequences. [file TPJ-123-0-s007.pdf]

SI Table S2: Sequences of steroidogenesis from plants without available genomes. Transcripts from the following NCBI bioprojects were analysed for species without available genome sequences.

Allium schoenoprasum (PRJEB55612 and PRJNA847160):

SCCE:
>*Allium schoenoprasum* TRINITY_DN5701_c1_g1_i8:211-1638
MAHYIAICSLSILIIWLIKLFQRWRNPRCNGKLPPGSLGFPLLGETFQFFAPSTTFDIHPFVKERM
NRYGPIFKTSLVGRPIIVSTDHELNNFVFQQEGKLFQSWYPDTFTEIFGRNNVGELHGFLYKYL
KSLVLKLFGPESLKERLLSDIEKSACINLRTWSLQPSVDLKEGIATMIFDLTAKKLISYDPSTSSE
NLRHNFVAFIKGLISFPVNIPGTAYNKCLQGRRKAMRVLKQMLAERKSRPNRQCNDFFDYVV
EELKKERPVLTESVALDLMFVLLFASFETTSLALTLAIKLIADHPKVLEGLTEEHEAIIRNREDP
ESEITWQEYKSMTFTLQVINETARLANIVPGIFRKVLKEIHVNEYTIPAGWGVMVCPPAVHLN
SEIFEDPLSFNPWRWKNKAELNGGSKNFMAFGGGMRFCVGTDFTKLQMAVFIHCLVTKYRW
KMIKGGNIIRTPGLGFPDGYHVELLSKE*

3β-HSD:
>*Allium schoenoprasum* TRINITY_DN12050_c1_g2_i1:72-896
MSKLRLEGKVAIITGAASGIGETSAELFVANGAIVVIADIQDELGQRVAHRINVSSAFKGEDRC
TYGHCDVTDEKQVQDTVDCTIATYGRLDIVYSNAGILGSPTNIADLDLAELNRIMTVNVGGA
LAIIKHGARAMIAKGIRGSILCTASVAAQRAGLGPVAYTTSKHALLGLVRAAAGEYGPYGIRV
NCVSPFGVATPLSCGLDGVSPDVVESIVESVATLKGVRLKTYHVAEAALFLVSDQSAYISGHD
LVVDGATTTCSSSKFGMSSDVA*

KSI:
>*Allium schoenoprasum* TRINITY_DN25223_c0_g1_i2:417-1589
MSGKEMLQAGRVDQVNINGTCNILDVCHDVGVKRLVYVSTYNVVFGGKEIINGNESLPYFPL
DEHVDNYGRSKSVAEQLVLKCNGRPSKKNNGVRLYTCAIRPAAIYGPGEERHLPRILDLAKM
GLLSFKVGDSHVKTDWVYVDNLVLSLILASMGLLDDIPGRTHPIAAGQAYFICDGSPVNTFDF
IIGPLLKNLEYGLPSITLDVNHAFTLSRVISAFYTLLYPWLNRRWLPNPLLLPAEVYKIGVTHYF
SYLKAREELGYFPMVTPQEGLSKTISYWKERKKEELEGPTLLTWCLVISGMSALFAAAYLPPV
GLLKYLQAIALFMFRSLWLTRLVFVTAVAVHVVEGIYAWFLARRVDPKNSCGWLWQTVAL
GIFSLRFLLKRANIM*

Steroid 5α-reductase:
>*Allium schoenoprasum* TRINITY_DN30024_c0_g1_i11:58-825
MEDYSLYTAALKTLYISAVINVISLPFISAPYGKHFRGGWGPTIPASLAWFLMESPTIFLTICFYP
FGRHVFHPLSLLLVFLYLFHYCNRTLVFPQRLRKGAKGFPVSVAAMAFVFNLLNAYVQTRSL
SHYSVYEERLSIWVMVRVLIGVMVFAWGMWVNVQSDLALLRLKKESGGVYKIPRGGWFEY
VCNPNYMGEAAEWFGWAVVAWSPTAWGFFFYTCSNLVPRARANLRWYREKFGEEYPKSM
KAILPFLF*

Steroid 5β-reductase/ PRISE:
>*Allium schoenoprasum* TRINITY_DN13856_c0_g1_i2:c1642-455
MSWWWAGAIGAARKTLDSSSSSNPNSLPPPQSIALVVGATGIVGTSLLDILPLPDTPGGPWKL
YAVSRRPLVSDNPRIHPIQCDVSDSSQTLDRLSPLSDVTHIFYVAWANCSSEAQNLKVNSSML
RNVLNAVLPNAPNLQHICLLTGRKHYIGAFEFIDKVKPHDPPFYEEMPRLESPNFYYDMEDILF
EELEKKDGKVSWSVHRPTTIFGFSSRAAMNVVQGVCVYAAMCKKEKKVMRWPGSKVTWD
GFSDASDADLVAEHQIWAAVDPYAKNEAFNCSNGDVFKWKQLWKALAEQFEVEWVGYQG
EDTRFSLKEEMKGKEKVWEEVVKENELVETKLEEVGNWWFLDSVLGIDFAHLDSMNKSKEH
GFLGFRNTLNSFNSWVDKMKAFKIVP*

Borago officinalis (PRJEB50033):

SCCE: not detected!

3β-HSD:

>*Borago officinalis* TRINITY_DN148989_c0_g1_i1:37-843
MKIYFDFRLEGKVALITGAASGIGEETVRLFAEQGAYVVGADIQDELGQQVIESIGSDKVSYH
HCDVRDENQVEQTVKYTLQKYGDLNILFSNAGIMGPLSSILELNLSEFDDTLATNVRGVVATI
KHVGKAMVERKTKGSIVCTASVSACMGGTGPHAYTTSKHALIGLIRTACGELGNYGIRVNSIS
PYGVATPLACNSYHLEPAQVEENSCATANLKGVVLKPKHIAEAALFLASDESGYISGHNLVV
DGGFTVFNQSLSKFQEV*

KSI:

>*Borago officinalis* TRINITY_DN50257_c0_g1_i2:514-1698
MSGKEMLQYGRVDQVNINGTCHVIDACIEKGVGRLVYVSTYNVVFGGKEITNGNESLPYFPI
DDHVDPYGRSKAIAEQLVLKSSGRPFKKKSGCLYTCAIRPAAIYGPGEERHLPRIINLAKLDLL
PFKIGGPNVKTDWVYVDNLVLALILASMGLLDDIPGKEGKPVASGQPYFISDGSPVNTFEFLR
PILKSLDYDLPKTTVKVSNALLLGNFFWGLYSLMYPLLSKTWLPQPLLLPAEVYKVGVTHYF
SYLKAKEELGYIPIVSSREGTAATISYWQERKSSTQGPSIYTWLFVVAGMLWVFAASYLPDIGP
APFLRAVALFVFRSMWLLQRVFILSAAAHIGEGIYAWILARKVDPENATGWFWQTFALGIFSL
RYLLKKRKLKAQVDQN*

Steroid 5α-reductase:

>*Borago officinalis* TRINITY_DN6153_c0_g1_i9:c1186-425
MISDKTLFNYAILTLYLSTPPTIISLLFLTAPYGKHRRSGWGPDIPAPLAWFFMEAPTLWLTFLL
FPFGQNYTNSKSFILISPFLFHYLNRTIIYPIRIKNSANRFPLSVALMAFVFNLLNAYIQARWVSH
YADLREDEWFWWRFSGGLVVFIVGMRINVQSDNSLLRLKCQGGGYRIPRGGWFEYVSCPNY
FGEVLEWLGYSVMTWSYAGFAFFVYTCANLVPRARANHQWYLDKFGKDYPKNRKCVIPFL
Y*

Steroid 5β-reductase/ PRISE:

>*Borago officinalis* TRINITY_DN24783_c0_g1_i4:c1787-657_partial
KKLEEDEPPTKYQSVGLIIGVTGIIGNSLAEILPLSDTPGGPWKVYGVARRPRPSWNIDHPIEYI
QCDILDKEDCQNKLSKLTDVTHLFYVTWANGSNEPENCQLNGKMFRNLVDVVIPNCPNLSHI
CLQTGRKHYAGPFESMGKVAHDPPFYEDLARLDVPNYYYVLEDILFSEVEKKEKLTWSVHRP
GSIFGFSPYSMMNIVGGLCVYAAVCKYMNLPLRFPGCKEAWDGYSDCSDADLVAEHQIWAA
VDPYAKNEAFNVSNGDVFKWKHFWRVLAEQFGVECADFEEGTSKFSLKEMMKDKAGVWE
EIVEANGLVATKLEEVGCWWYIDLVLGIAPMLDTMNKSKEHGFLGFRNSKNAFISWIDKVKV
HKIVP*

Myosotis sylvatica (PRJNA451174):

SCCE:

>*Myosotis sylvatica* TRINITY_DN45926_c0_g1_i2:c1246-329_partial
EKYGSIFRTSIVGRPVIVSADSDLNYYIFQQEGQLFQSWYPDTFTEIFGKQNVGSLHGFMYKYH
IFVELKDSTARMIFDLTAKKLISYDSEKSSENLRENFVAFIQGLISFPLDVPGTAYHKCMQGRK
KAMKMLKNLLQERRANPRKEQTDFFDYVVQELKREDTVLTEAIALDLMFVLLFASFETTSLA
ITLAVKFLSENPLVLRQLTEEHEAIIRKRENPDSGVTWQEYKSMTFTFQFINETVRLANIVPAIF
RKTLRDIKFKGYTIPAGWAVMVCPPAVHLNPARYTNPLEFNPCRWEGVELHGVSKNFMAFG
GGMRFCVGTDFTKVQMAVFLHSLVTKYKWKLIRGGQTVRTPGLQFPEGMHIHVTKKDRDAP
AE

3β-HSD:

>*Myosotis sylvatica* TRINITY_DN7591_c0_g1_i2:69-857
MSKLRLEGKVALITGGASGIGEETVRLFVEEGAFVVAADIQDELGQQLIQSISSDKVSYHHCD
VRDEKQVEETVNYTVQKYGELNVLFSNAGIIGPLTSILELNLSEFDDTIATNVRGVLATIKHAG
RAMVERKTKGSIVVTASVAACVGGTGPHAYTTSKHALIGLIRTACGELGNYGIRVNSISPYGV
ATPLTCKAYHLEASDVEENSCATANLKGVVLKATHVAETALFLASDQSAYVSGHNLVVDGG
FTVFNQSLSKY*

KSI:

>*Myosotis sylvatica* TRINITY_DN1104_c0_g1_i3:452-1606
MLQYGRVDEVNINGTCHVIDACVEKGARRLVYVSTYNVVFGGKEITNGNESLPYFPISDHVD
PYGRSKSIAEQLVLKSNGRPFKKKDGCLYTCAIRPAAIYGPGEERHLPRIVNLAKLGILPFTVG
EPHVKTDWVYVDNLVLSLILASMGLLDDIPGKEGKPVASGQPYFISDGSPVNSFEFLRPILNSL
DYDLPKRTLTVSHALLLGNFFWALYSLMYPWLRKTWLPQPLMLPAEVYKVGVTHYFSYLKA
KEELGYTPMVSPREGMAATISYWQERKSSVQGPTIYNWLFSVVGMLWVFAAAFLPDIGPVPF
LRAVGLFFFRSIGALQTLFILAAAAHIGEAIYAWKLAKKIDPANATSWFWQTFALGFFSLRFLL
KKRKSKV*

Steroid 5α-reductase:

>*Myosotis sylvatica* TRINITY_DN23071_c0_g3_i1:92-865
MFSEQTFFNYALLSIYLITPPTIISLRFLTAPYGKHHRAGWGPNIPAPLAWLLMESPTLWLTYLL
FPLGRNQPNPKARLLIAPFLLHYLHRTLLYPLRLLLRRGGPSNAFPVSVSLLAFAFNLLNSYLQ
ARWVSHYADFDTDHWFWPRFVLGLGVFGAGMWINVKCDNELLALKSKGGGYRIPRGGLFE
YVSCPNYFGEVLEWFGWAVMTCSFVGFAFFAYTCANLVPRARDNHKWYLKKFGEDYPENR
KCIIPFIY*

Steroid 5β-reductase/ PRISE:

>*Myosotis sylvatica* TRINITY_DN40999_c0_g1_i1:385-1554
MSWWWSGAIGAAKKKFEEDEQPTKYESVGLIIGVTGIVGNSLAEILPLSDTPGGPWKVYGVA
RRPRPSWNADHPIEYIQCDILDKEDCQSKLSKLVDVTHLFYVTWANKSTESENCEVNGKMFR
NLLDVVIPNCPNLQHICLQTGRKHYLGPFESMDKVAHDPPYYEDLPRLDVPNFYYVLEDILFS
EVEKKEKLTWSVHRPGHIFGFSPYSMMNIVGTLCVYATICKHMNLPFRFPGCKEAWEGYADC
SDADLIAEHHIWAAVDPYAKNEAFNVSNGDVFKWKHFWRVLAEQFGVECAEFEEGEQKLSL
QELMKDKAVVWDEIVEANGLLATELGEVATWWFVDLVLGIPQTLDTMNKSKEHGFLGFRNS
KNAFISWIDKVKVHKVVP*

Plantago lanceolata (PRJNA636383):

SCCE:

>*Plantago lanceolata* TRINITY_DN21777_c0_g1_i1:1-954_partial
YKYFKNMVLSLFGPESLKRMITDVENQSRFTLQNWSNNPSVELKDGIAKMIFGLTAKKLISCD
SEESCDNLRKNFVDFVDGLISFPLNFPGTAYYRCLQGRKKAMKTLKNMFQERRKTPQAIQSD
FFDYVLEELEKKDTILTEAIALDLMFVLLFASYETASLATTMATKFLADNPLALQKLTEEHETI
VNLREDPDSGLTWKEYKSMTFTFQVINETLRLANIAPGIFRKAMVDTKFKEYTIPAGWSVMV
CPPAVHLDPKKYPNPLDFNPWRWDGLDTSVGSRNFMAFGGGMRLCIGADFTKVQMAVFLH
CLVTKYK*

3β-HSD:

>*Plantago lanceolata* TRINITY_DN61292_c0_g1_i1:c1004-105
MTAQVLPELSFAGIQFLNKDMCSPHHPIKRLEGKVAIITGGARGIGEATVKVFASQGARVVIA
DVEDMLGNSLAQSLGPNVTFVHCDVTSEEDIENVVTSTVSKYGKIDILFNNAGVLGDQSKHK
SILDFDADEFDRIMRVNVRGAMLGIKHVARAMIRYGGGGCVISTASVAGVMGGLGPHSYTAS
KHAIVGMTKNAACELGRYGIRVNCISPFGVATKMLVDAWRVGGDNGSAGVTESEVEKIEEF
VRGLGNLKGETLRTKDIAEAALFLASDESRYISGHNLVVDGGVTTSKNCVGL*

KSI:

>*Plantago lanceolata* TRINITY_DN3786_c0_g1_i27:5-1306_partial
RPDSPWSDDLRNRGVRCLQGDVARKEDVEKALRGADCVFHLASYGMSGKEMLRYSRVDEV
NINGTCHILDACLDYGIGRLVYVSTYNVIFGGKEIVNGNESLPYFPLDNHEDPYGRSKSIAEQL
VLKSNGKPFKKKQGKLYTCAIRPAAIYGPGEERHLPRIMNLAKLGLLPFKIGSRNVKSDWVY
VDNLVLSLLLASMGLSDDIPGRVGQPIAAGQPYFISDGSPVNSFEFLQPLLKSFDYDLPQSSLA
VPHALFLGKVFWAIYSLVYPWLRQRWIPQPLILPAEVYKVGVTHYFSFLKAREELHYAPMVS
PQEGMNATIEYWKERKRNEIDGPTIYAWLFVIIGMTMLFCAACMPDVGPFHLCRAIYLFFFRS
MLTVRILFFSSVAAHIGEGVYAWQLAKKADPANAKGWFWQTAAMGYFSLRFLLKKAKK*

Steroid 5α-reductase:

>*Plantago lanceolata* TRINITY_DN35496_c0_g1_i1:139-951
MVFSDEQIYHYALLTLYLITPLTFLSLQFLTAPYGKHNRPGWGPTIPPPIAWCLMESPTVFLSLL
LFPRGRNHLNPRAYLLISFFLLHYLHRTFIYPLRLFLKSISPNSPKPGSVNPGSVKPGFPVSIALT
AFVFNLLNGYLQSRWVSEYAELDSDRWFWYRVVGGGAVFLGGMAANIWSDNFLMRLKESG
GGYRVPRGGLFEWVTSPNYFGEIVEWLGWAFMCWSWAGLGFFLYTCANLVPRAASGRKWY
LEKFGEDFPKHRKAVIPFLY*

Steroid 5β-reductase/ PRISE: Previously published on NCBI (WKF48834.1; Dorfner et al., 2024).

>*Plantago lanceolata* WKF48834.1
MSWWWAGAIGAAKKRSDEDDAPPKHASVALIVGVTGIVGNSLAEILPLADTPGGPWKVYGV
ARRPRPAWNEDNPINYIRCDVSDPEDTKEKLSPLTDITHVFYVTWANRSTESENCEANGKML
KNVLDVVIPNCPDLKHISLQTGRKHYCGPFELLGKIESHDPPFTEDLPRLKCENFYYTQEDLLF
EEVEKREGLTWSVHRPGNIFGFSPYSMMNLVGTLCVYAAICKHEGKVLRFPGCKAAWDGYS
DCSDADLIAEHHIWAAVDPYAKNEAFNVSNGDVFKWKHFWKVLAEQFGVECGEYEEGEDL
KLQDLMKGKEPVWEEIVRENALSPTNLEDVGVWWFSDLILGFPCPLDSMNKSKEHGFLGFRN
SKNSLISWIDKAKAYKIVP*

***Pelargonium zonale* (PRJNA807121):**

SCCE:

>*Pelargonium zonale* TRINITY_DN19848_c0_g1_i38:c1744-299
MLTLQVQQYVVCFVGVVVAGISVWIYKWANPKCNGKLPPGSMGFPFFGETFEFFTPHHFYGI
PPFVRKRISRYGSVFRTSLVGRKVVVSTDPEINYSIFQQEGKSFLIWYTESFIKILGQQSMLAYH
GIVHKYLKNLILHLVSPENLREKLLLEMDSATRRHLQSWATHGSVDVKEATSTMIFEYFSKKL
MSYDEPRALENLKNNYNAFIDGLISFPLNIPGTAYHACLKGRQNAVKVIKDIFRERKRSKIRYN
DFLDHLLEEMEKEGSILDEAIAIDLVFVLLFATYETTSAAITLLTKFISEHQEVVAELMREHDAI
VRSRDQDKDSEVTWKEYKSMTFTHMVINETVRLANIVPGIFRKVVKDVEIKGYTIPAGWVV
MIVPSVVHLNPDKFEDPLAFNPWRWQGQELHAGSKSFMAFGGGLRLCVGADFAKLQMALFL
HHLLTKYRWTVTKGGDITRRPGLVFPTGFHVEIQHKTPIA*

3β-HSD:

>*Pelargonium zonale* TRINITY_DN15032_c0_g1_i1:c910-128
MSTPRLQGKVAIVTGGASGIGEEAARLFCENGAFVVVADVQDELGQSVVASINSKSADRASY
HHCDVRDEKQVEETVSFAVQKYGTLDVMFSNAGIYGPAPGIMEVDLELFDNTMATNVRGVA
ATIKHAARAMVAKKVRGGSIICTASVAASVGGMGPSAYTASKHAVVGLVRAACAELGAHGI
RVNCVSPFGVATPLACTAVNLEASELESGTSALANLKGIVLKARHIAEAALFLASDDSAYTSG
LNLNVDGGFTVVR*

KSI:

>*Pelargonium zonale* TRINITY_DN2732_c1_g1_i3:c1458-274
MSGKEMLQFGRVDEVNINGTCHVLEACLEFEIKRLVYVSSYNVVFGGKEIVNGNESLPYFPID
DHVDSYGRSKSIAEQLVLKSNNRPFKKDTGKCLYTCAVRPAAIYGPGEERHLPRIISLAKLGLL
PFKIGEQSVKQDWIYVDNLILALILASMGLLDDIPGRPKHPVAAGQAYFVSDGSPVNTFEFIRP
LLRSLDYELPKPSLTIHQALILGKFFEVVYTILYPWLNRRWLPQPFILPAEVYKVGVTHYFSFL
KAREELGYVPMVTPQEGMAATVAYWQERRLKTVDGPTIYVWLFCVFGMFQLFCGAFLPPVG
PVRLLRAISLFFFRSLWIVRLVFILAAGAHIGESLYAWRLAKRVDPENSVRWFWQTFALGFFSL
RFLLKKAKQYKRVE*

Steroid 5α-reductase:

>*Pelargonium zonale* TRINITY_DN2186_c2_g1_i2:43-663_partial
AWFLMESPTLWLSLLLLLKHPLNPRSLLLFSPFLLHYLNRTILFPIRLLRLRNRRSPNFPASIAA
MAFAYNVLNSYVQARSVFEFGDYDSDRWFWWRFGAGLVVFSGGMAVNVWADSVLVRLRR
EGGGYRVPIGGWFEVVSCPNYFGEIVEWFGWAVMCGSWAGFGFFVYTCANLVPRARANHR
WYLEKFGEDYPKKRKAVIPFLY

Steroid 5β-reductase/ PRISE:

>*Pelargonium zonale* TRINITY_DN701_c2_g1_i1:294-1469
MSWWWAGAIGAAKKKFDDDEAQLPRTFQSTALVVGVTGIVGNSLAEILPLSDTPGGLWKVY
GVARRPRPNWNADHPIEYIQCDVSDPQDSQSKLSTLTDVTHVFYVAWANRSTEAENCKVNS
DMFRNVLRAVIPNAPNLRHVCLQTGTKHYIGSFESFGRSQPHDPPFTEDIPRLSGPNFYYDLED
VMFQEVAKKEGLTWSVHRPDLIFGFSPYSLMNIVGTLAVYAAICKHEGTPLWFPGSKAAWES
YQVASDADLVAEQHIWAAVDPYARDEAFNCNNGDVFRWKQFWKVLAEQFGVEKHGFEEG
KNVKLSEMMKDKGHVWEEIVRVNQLQPTKLEEVGVWWFADTVLGGEGMLSSMNKSKEHG
FVGFRNSKNSFVSWIDKVKSFKIVP*

Ribes rubrum **(PRJNA1131118):**

SCCE: not detected!

3β-HSD:

>*Ribes rubrum* TRINITY_DN197036_c0_g1_i1:c874-92
MSKPRLEGKVAIITGAASGIGEETVRLFVENGAFVVVADVQDELGRKVVASIGSEKVAFHHC
DVRDEKQVEETINFTIEKYGTLDVLFSNAGVMGPLTGILDLDLNEYDNTMTTNVRGVAATIK
HASRAMVARKTRGSIICTASVAGSIGGAGPLGYTTSKHALVGLVRSACSELGKYGIRVNCISPF
GIATPLSCIAYDLKPSEVENNSCDLANLKGIVLKPKHVSQAAVFLASYESAYISGHNLALDGGF
TVVNHSTF*

KSI:

>*Ribes rubrum* TRINITY_DN1856_c0_g1_i4:c1494-328
MSGKEMLQFGRVDEVSINGTCHILDACVEFGIKRLVYVSTYNVVFGGKEIVSGNETLPYFPLD
DHVDPYGRSKSVAEQLVLKSNGRPTKEKGKCVYTCAIRPGAIYGPGEERHLPRIVSLAKLGLI
PFRIGEANVKTDWVFVDNLVLGLILASMGLLDNIPGKGKHPIAAGQPYFISDGSPVNSIEFLRP
LLTSLDYDLPKASITVPHALVLGRIFSAIYTILYPWLNRWWLPQPLILPAEIYKVGVTHYFSYL
KAKEELGYIPMVTPREGMASTISYWQERKRRMLDGPTIYTWLFCVIGMTSLFAVACLPDFVP
VPLFRSLSLFIFRSIWTVRLLFLFAAAAHIGEAFYAWHLAKRVDPANKRGWFWQTFALGIFSL
RLLLKRARK*

Steroid 5α-reductase:

>*Ribes rubrum* TRINITY_DN6274_c0_g1_i8:415-1182
MDSDQTLFHYSLLTLYITAPPTYISLRFLQAPYGKHHRPGWGPTMSPPLAWFLMESPTVWLTL
FLFPLGRHSSNPKALILILPFLIHYLHRTILYPIRLTRRKTTSGFPVSIALMACGFNLLNSYLQAR
WVSHYNDYEGVGWFWWRFVIGLVVFVSGMAVNIWSDSVLVGLKSGGGGYKVPKGGWFEM
VSCPNYFGEILEWLGWAVMTWSWAGFGFFLYTCANLVPRARANHQWYLEKFKEDYPKGRK
AVIPFLY*

Steroid 5β-reductase/ PRISE:

>*Ribes rubrum* TRINITY_DN313_c0_g1_i33:c1382-210
MNWWWAGAIGAAKKKSEEDEAPRSYQSVALIIGVTGVVGNSLAEILPLSDTPGGLWKVYGV
ARRERPNWNADHPIEYIQCDVSDPEDTMAKLSPLTDVTHIFYVTWTNRLTEIENCKANGAMF
RNVLAAVIPSALDLRHICLQTGTKHYMGPFDSLGKIQPHDPPFTEDLPRLEVPNFYYTLEDILF
ETAEKKEELTWSVHRPQAIFGFSPYSMMNIIGTLCVYAAICKHEGKPLKFPGVKSAWECYSTA
SDADLIAEHHIWAAVDPYAKNEAFNCSNGDVFKWKHLWKVLAEQFGIECVEFDENEPRVTL
QELMKGKEAVWDEIVKENELQPTKLEEVGLWWLSDLVFSGEALLDSMNKSKEHGFLGFRNS
KNSLITWIDKMKAYKIVP*

References:

Dorfner M, Klein J, Senkleiter K, Lanig H, Kreis W, Munkert J. 2024. Addressing the Evolution of Cardenolide Formation in Iridoid-Synthesizing Plants: Site-Directed Mutagenesis of PRISEs (Progesterone-5β-Reductase/Iridoid Synthase-like Enzymes) of Plantago Species. *Molecules* **29**: 5788.
