# Supplementary material for: Progestogens and androgens influence root morphology of angiosperms in a brassinosteroid‐independent manner
Source: Plant J. 2025 Sep 9;123(5):e70459. doi: 10.1111/tpj.70459 (PMC12419790; doi:10.1111/tpj.70459)
Supplement: Supplementary file 8 — Table S3. List of packages needed for conducting the statistical root length analysis in Rstudio. [file TPJ-123-0-s008.pdf]

SI Table 1 - List of packages needed for conducting the statistical root length analysis in Rstudio.

Package	Version	Reference
ARTool	0.11.1	Wobbrock et al. (2011)
car	3.1-2	
carData	3.0-5	
dplyr	1.1.4	Wickham et al. (2023)
emmeans	1.10.1	Lenth (2024)
ggplot2	3.5.1	Wickham (2016)
ggpubr	0.6.0	Kassambara (2023a)
magrittr	2.0.3	Bache and Wickham (2022)
psych	2.4.3	Revelle (2024)
rstatix	0.7.2	Kassambara (2023b)
tidyr	1.3.1	Wickham et al. (2024)

Wickham H (2016): ggplot2: Elegant Graphics for Data Analysis: Springer-Verlag New York. Available online at https://ggplot2.tidyverse.org, [06.05.2024].

Wickham H, Francois R, Henry L, Müller Kl, Vaughan D (2023): dplyr: A Grammar of Data Manipulation. Available online at https://github.com/tidyverse/dplyr, [06.05.2024].

Wickham, H; Vaughan, D; Girlich, M (2024): tidyr: Tidy Messy Data. R package version1.3.1. Available online at https://tidyr.tidyverse.org, [06.05.2024].

Wobbrock J O, Findlater L, Gergle D, Higgins J J (2011): The aligned ranktransform for nonparametric factorial analyses using only anova procedures. In: **Desney T, Geraldine F, Carl G, Bo B, Wendy A K** (Eds.): CHI 2011. Conference proceedings and extended abstracts; the 29th Annual CHI Conference on Human Factors in Computing Systems; Vancouver, BC, May 7 -12, 2011. CHI '11: CHI Conference on Human Factors in Computing Systems. Vancouver BC Canada, 07 05 2011 12 05 2011. New York, NY: ACM, pp. 143–146.

Bache S M,Wickham H (2022): magrittr: A Forward-Pipe Operator for R. Available online at https://magrittr.tidyverse.org [06.05..2024].

Lenth R V (2024): emmeans: Estimated Marginal Means, aka Least-Squares Means. Available online at https://ggplot2.tidyverse.org. [06.05.2024]

Kassambara A (2023a): ggpubr: 'ggplot2' Based Publication Ready Plots. R package version v0.6.0. Available online at https://rpkgs.datanovia.com/ggpubr/, checked on [06.05.2024].

Kassambara A (2023b): rstatix: Pipe-Firendly Framework for Basic Statistical Tests. R package version 0.7.2. Available online at https://rpkgs.datanovia.com/rstatix/, [06.05.2024].

Revelle W (2024): psych: Procedures for Psychological, Psychometric, and Personality Research. Available online at https://cran.r-project.org/web/packages/psych/psych.pdf. [06.05.2024]
