# Supplementary material for: Progestogens and androgens influence root morphology of angiosperms in a brassinosteroid‐independent manner
Source: Plant J. 2025 Sep 9;123(5):e70459. doi: 10.1111/tpj.70459 (PMC12419790; doi:10.1111/tpj.70459)
Supplement: Supplementary file 9 — Table S4. Brassinosteroid profiles of progesterone‐ or testosterone‐treated Arabidopsis thaliana. Here, we show brassinosteroid profiles of shoot and root tissues of wild‐type plants (A. thaliana Columbia‐0) and plants overexpressing DET2 (A. thaliana DET2 OE). In addition to the brassinosteroid profiles of these plant lines, we analysed putative changes in the brassinosteroid profiles caused by progesterone (PO) or testosterone (TO) treatment. For this experiment, we treated wild‐type plants (Col. 0) and the transgenic line (DET2 OE L4) with PO and TO and analysed the resulting brassinosteroid levels after 4 days. Each value represents a separate biological replicate which was methodologically divided into technical replicates (n ≥ 3), resulting in the listed SD. MS—plants grown on MS medium (mock treatment); DMSO—MS medium with DMSO (untreated control); PO—MS medium containing 0.03 mM progesterone; TO—MS medium with 0.03 mM testosterone. Col‐0—A. thaliana Columbia‐0; DET2 OE L2 – L7—A. thaliana DET2 OE lines 2–7; NQ—no quantification possible. [file TPJ-123-0-s004.pdf]

**SI Table S4: Brassinosteroid profiles of progesterone- or testosterone-treated *Arabidopsis thaliana*.** Here we show brassinosteroid profiles of shoot and root tissues of wild-type plants (*A. thaliana* Columbia-0) and plants overexpressing DET2 (*A. thaliana DET2* OE). In addition to the brassinosteroid profiles of these plant lines, we analysed putative changes in the brassinosteroid profiles caused by progesterone (PO) or testosterone (TO) treatment. For this experiment, we treated wild-type plants (Col. 0) and the transgenic line (DET2 OE L4) with PO and TO and analysed the resulting brassinosteroid levels after four days. Each value represents a separate biological replicate which was methodological-wise divided into technical replicates ($n \geq 3$), resulting in the listed SD. MS – plants grown on MS-medium (mock treatment); DMSO – MS-medium with DMSO (untreated control); PO – MS-medium containing 0.03 mM progesterone; TO – MS-medium with 0.03 mM testosterone. Col-0 – *A. thaliana* Columbia-0; DET2 OE L2 – L7 – *A. thaliana DET2* OE lines 2 – 7; NQ – no quantification possible.

## Shoots:

| treatment | organism | 28-norcastasterone pmol/g DW | 28-norcastasterone SD | Castasterone pmol/g DW | Castasterone SD | 28-homocastasterone pmol/g DW | 28-homocastasterone SD | 28-homobrassinolide pmol/g DW | 28-homobrassinolide SD | Homodolicholide pmol/g DW | Homodolicholide SD |
|---|---|---|---|---|---|---|---|---|---|---|---|
| MS | Col-0 | 269,37 | 87,16 | 109,00 | 48,81 | 11,23 | 1,07 | 1500,50 | 427,16 | 21,72 | 6,89 |
| | | 257,84 | 3,15 | 259,01 | 69,12 | 159,46 | 40,69 | 796,65 | 51,73 | 19,12 | 0,93 |
| | | 327,47 | 22,20 | 214,63 | 8,41 | 86,42 | 32,72 | 1294,00 | 5,78 | 14,03 | 2,08 |
| | | 74,24 | 5,95 | 150,00 | 7,67 | 1204,65 | 30,14 | 59,66 | 0,52 | 1,30 | 0,33 |
| | | 81,06 | 1,64 | 81,80 | 0,68 | 1524,01 | 17,80 | 54,52 | 12,04 | 0,84 | 0,22 |
| | DET2 OE L2 | 40,65 | 5,15 | 35,83 | 2,93 | 1282,43 | 57,31 | 173,37 | 19,47 | NQ | NQ |
| | | 32,29 | 3,56 | 46,30 | 8,48 | 1059,12 | 104,70 | 94,60 | 3,86 | 0,54 | 0,16 |
| | DET2 OE L4 | 71,41 | 6,47 | 84,34 | 17,61 | 648,68 | 158,20 | 226,46 | 58,51 | 2,51 | 0,82 |
| | | 76,80 | 2,67 | 144,80 | 5,88 | 625,16 | 56,16 | 131,49 | 30,90 | 2,01 | 0,71 |
| | | 84,30 | 2,11 | 185,54 | 24,33 | 870,88 | 86,07 | 126,60 | 42,06 | 1,59 | 0,62 |
| | | 56,29 | 0,80 | 206,99 | 41,40 | 683,59 | 190,02 | 59,07 | 9,01 | 0,50 | 0,08 |
| | DET2 OE L5 | 297,30 | 16,19 | 229,60 | 11,99 | NQ | NQ | 728,85 | 40,13 | 44,70 | 6,42 |
| | DET2 OE L6 | 236,86 | 13,71 | 407,61 | 23,21 | 39,68 | 6,00 | 876,38 | 53,08 | 38,77 | 8,11 |
| | DET2 OE L7 | 267,00 | 2,03 | 115,02 | 15,49 | 19,59 | 8,20 | 2608,73 | 105,87 | 56,17 | 3,31 |
| DMSO | Col-0 | 57,78 | 8,50 | 102,91 | 18,11 | 1581,93 | 46,53 | 39,94 | 6,04 | NQ | NQ |
| | | 55,71 | 4,07 | 159,77 | 11,15 | 1057,12 | 76,33 | 39,63 | 1,09 | 0,84 | 0,24 |
| | | 199,71 | 19,81 | 382,00 | 28,08 | 127,99 | 23,17 | 950,44 | 55,48 | 21,91 | 6,09 |
| | | 288,01 | 5,01 | 317,97 | 43,07 | 81,80 | 35,34 | 834,89 | 55,25 | 35,89 | 0,78 |
| | DET2 OE L4 | 45,87 | 11,52 | 108,19 | 5,36 | 663,84 | 197,68 | 51,02 | 15,87 | 0,93 | 0,29 |
| | | 93,18 | 5,79 | 146,63 | 7,47 | 800,27 | 52,41 | 36,54 | 5,08 | 3,66 | 1,34 |
| | | 108,10 | 1,16 | 211,03 | 20,92 | 864,47 | 79,11 | 43,69 | 5,45 | 1,70 | 0,59 |
| | | 90,61 | 7,54 | 269,93 | 11,81 | 861,35 | 101,85 | 23,41 | 7,65 | 1,51 | 0,60 |
| PO | Col-0 | 241,68 | 9,27 | 292,18 | 32,01 | 116,91 | 36,14 | 664,39 | 66,03 | 20,08 | 4,52 |
| | | 230,23 | 25,13 | 419,58 | 32,39 | 119,89 | 22,85 | 1032,68 | 66,81 | 31,67 | 6,10 |
| | | 95,73 | 2,71 | 116,25 | 3,54 | 1363,09 | 84,62 | 7,03 | 1,85 | 0,66 | 0,21 |
| | | 97,11 | 3,76 | 65,10 | 9,51 | 1376,41 | 97,82 | 11,21 | 1,46 | 1,22 | 0,65 |
| | DET2 OE L4 | 44,06 | 11,22 | 90,42 | 7,76 | 948,46 | 232,15 | 61,52 | 6,35 | 1,06 | 0,10 |
| | | 90,18 | 6,00 | 72,65 | 12,65 | 861,38 | 116,38 | 64,69 | 7,42 | 2,37 | 0,75 |
| | | 75,75 | 2,62 | 102,54 | 18,60 | 1399,11 | 76,76 | 33,99 | 7,06 | 0,56 | 0,03 |
| | | 89,25 | 4,09 | 95,74 | 11,27 | 1403,78 | 75,59 | 26,54 | 9,33 | 0,88 | 0,27 |
| | | 98,91 | 2,13 | 81,61 | 13,26 | 1047,73 | 43,95 | 175,61 | 16,03 | 1,99 | 0,55 |
| TO | Col-0 | NQ | NQ | 155,77 | 24,00 | 1657,37 | 76,63 | 61,46 | 2,14 | 1,45 | 0,43 |
| | | 54,12 | 9,39 | 114,53 | 5,49 | 1357,34 | 187,85 | 39,88 | 4,80 | 0,87 | 0,35 |
| | | 281,82 | 2,68 | 386,40 | 37,97 | 84,69 | 19,21 | 422,10 | 3,28 | 20,45 | 4,37 |
| | | 202,71 | 3,51 | 285,57 | 18,22 | 58,91 | 22,96 | 930,83 | 31,42 | 24,90 | 6,95 |
| | DET2 OE L4 | 34,32 | 8,73 | 59,09 | 8,24 | 985,61 | 45,51 | 145,28 | 24,43 | 1,95 | 0,42 |
| | | 73,16 | 1,54 | 62,57 | 10,45 | 841,44 | 99,69 | 60,49 | 7,45 | 1,88 | 0,23 |
| | | 80,03 | 9,10 | 87,08 | 10,97 | 1607,98 | 152,03 | 33,70 | 5,32 | 0,76 | 0,16 |
| | | 66,77 | 3,70 | 89,21 | 3,98 | 1799,42 | 142,75 | 42,00 | 14,72 | 1,35 | 0,56 |
| | | 83,61 | 4,01 | 84,53 | 2,65 | 1010,12 | 202,08 | 98,38 | 5,24 | 2,52 | 0,50 |

| | |
|---|---|
| (green) | 0-10 |
| (light green) | 10-50 |
| (light grey) | 50-100 |
| (grey) | 100-500 |
| (lavender) | 500-1000 |
| (white) | >1000 |

# Roots:

| treatment | organism | 28-norcastasterone pmol/g DW | 28-norcastasterone SD | Castasterone pmol/g DW | Castasterone SD | 28-homocastasterone pmol/g DW | 28-homocastasterone SD |
|---|---|---|---|---|---|---|---|
| MS | Col-0 | NQ | NQ | 10,05 | 3,24 | 6306,85 | 601,79 |
| | | 67,30 | 6,67 | NQ | NQ | 261,98 | 94,37 |
| | | 23,83 | 9,29 | 3,76 | 0,76 | 331,90 | 117,54 |
| | DET2 OE L2 | 20,92 | 3,53 | 0,66 | 0,20 | 230,05 | 46,41 |
| | | 23,97 | 6,31 | NQ | NQ | 286,54 | 76,49 |
| | DET2 OE L4 | 19,55 | 4,05 | 6,70 | 1,81 | 639,98 | 182,99 |
| | | NQ | NQ | 6,68 | 2,99 | 299,60 | 37,37 |
| | DET2 OE L6 | NQ | NQ | 207,39 | 18,69 | 277,12 | 21,32 |
| | DET2 OE L7 | 51,34 | 1,28 | NQ | NQ | 168,99 | 30,54 |
| DMSO | Col-0 | NQ | NQ | 9,39 | 2,69 | 2674,52 | 131,96 |
| | | 36,47 | 11,79 | 5,64 | 2,28 | 517,67 | 235,14 |
| | | 172,97 | 46,80 | 53,10 | 9,48 | 171,70 | 34,55 |
| | DET2 OE L4 | NQ | NQ | NQ | NQ | 1825,70 | 1018,76 |
| | | 20,95 | 3,56 | 3,64 | 0,00 | 313,11 | 84,67 |
| PO | Col-0 | 38,35 | 4,27 | 32,78 | 12,42 | 5297,07 | 2235,44 |
| | | 58,98 | 18,79 | 4,98 | 2,25 | 327,99 | 29,60 |
| | DET2 OE L4 | 11,18 | 4,39 | 2,98 | 1,25 | 1337,35 | 70,69 |
| | | 15,69 | 7,41 | NQ | NQ | 2691,73 | 391,22 |
| | | 42,20 | 5,66 | NQ | NQ | 2287,92 | 983,59 |
| TO | Col-0 | NQ | NQ | 6,55 | 4,78 | 8095,29 | 1897,46 |
| | | 99,83 | 24,69 | 17,43 | 5,16 | 556,54 | 246,50 |
| | DET2 OE L4 | NQ | NQ | NQ | NQ | 1585,62 | 286,84 |
| | | 15,73 | 2,06 | NQ | NQ | 1690,72 | 240,20 |
| | | NQ | NQ | NQ | NQ | 4333,59 | 336,71 |

| |
|---|
| 0-10 |
| 10-50 |
| 50-100 |
| 100-500 |
| 500-1000 |
| >1000 |

| treatment | organism | Homodolicholide pmol/g DW | Homodolicholide SD | 28-homobrassinolide pmol/g DW | 28-homobrassinolide SD | Brassinolide pmol/g DW | Brassinolide SD | 28-norbrassinolide pmol/g DW | 28-norbrassinolide SD |
|---|---|---|---|---|---|---|---|---|---|
| MS | Col-0 | 1,11 | 0,57 | 49,75 | 7,25 | 0,28 | 0,13 | 3,25 | 1,06 |
| | | 73,97 | 2,23 | 90,39 | 18,75 | | | | |
| | | | | 3,62 | 1,50 | | | | |
| | DET2 OE L2 | 0,33 | 0,10 | 6,15 | 2,19 | | | | |
| | | 0,15 | 0,02 | 2,53 | 0,74 | | | | |
| | DET2 OE L4 | 0,44 | 0,22 | 5,05 | 2,18 | | | | |
| | | 0,35 | 0,00 | 6,20 | 2,86 | | | | |
| | DET2 OE L6 | 104,44 | 2,15 | 125,79 | 13,81 | | | | |
| | DET2 OE L7 | 97,58 | 9,16 | 43,67 | 18,45 | | | | |
| DMSO | Col-0 | 0,64 | 0,07 | 118,95 | 8,10 | 0,29 | 0,04 | 3,75 | 0,83 |
| | | | | 3,53 | 1,69 | 0,61 | 0,14 | 7,34 | 1,06 |
| | | | | 7,20 | 1,45 | | | | |
| | DET2 OE L4 | 1,52 | 0,52 | 5,64 | 2,25 | | | | |
| | | NQ | NQ | 5,78 | 0,75 | | | | |
| PO | Col-0 | 0,63 | 0,08 | 63,54 | 6,60 | 0,39 | 0,14 | 4,25 | 0,85 |
| | | | | 8,19 | 3,31 | | | | |
| | DET2 OE L4 | 0,57 | 0,24 | 17,80 | 5,21 | | | | |
| | | 1,08 | 0,46 | 14,63 | 5,52 | | | | |
| | | 0,57 | 0,05 | 22,55 | 4,26 | | | | |
| TO | Col-0 | 0,73 | 0,03 | 70,24 | 2,59 | 0,19 | 0,09 | 6,10 | 1,52 |
| | | | | 7,25 | 2,16 | | | | |
| | DET2 OE L4 | 1,16 | 0,32 | 19,94 | 4,98 | | | | |
| | | 0,45 | 0,13 | 15,17 | 0,42 | | | | |
| | | 1,40 | 0,55 | 21,65 | 7,44 | | | | |
