# Supplementary material for: Progestogens and androgens influence root morphology of angiosperms in a brassinosteroid‐independent manner
Source: Plant J. 2025 Sep 9;123(5):e70459. doi: 10.1111/tpj.70459 (PMC12419790; doi:10.1111/tpj.70459)
Supplement: Supplementary file 13 — Table S8. Progestogen and androgen levels in A. thaliana roots after 8 days of treatment. We here give the levels of progestogens and androgens in A. thaliana roots in ng mg−1 dry weight after 8 days of treatment with DMSO (mock control), progesterone (30 μM) and testosterone (30 μM). Data are given as mean ± standard deviation; n ≤ 3. [file TPJ-123-0-s009.pdf]

SI Table S8: Progestogen and androgen levels in *A. thaliana* roots after 8 days of treatment. We here give the levels of progestogens and androgens in *A. thaliana* roots in ng mg^{-1} dry weight after 8 days of treatment with DMSO (mock control), progesterone (30 µM), and testosterone (30 µM). Data are given as mean ± standard deviation; $n \leq 3$.

DMSO control:

	**Shoots**	**Roots**
Pregnenolone	0	0
17α-hydroxypregnenolone	0	0
Progesterone	0	0.2 ± 0.03
17α-hydroxyprogesterone	0	0
5α-dihydroprogesterone	0	3.2 ± 0.2
DHEA	0.6 ± 0.06	1.1 ± 0.06
Androstenedione	0	0
Testosterone	0	1.3 ± 0.15
5α-dihydrotestosterone	0.18 ± 0.03	0.02 ± 0
**Total:**	**0.73**	**5.82**

Treatment with 30 µM progesterone:

	**Shoots**	**Roots**
Pregnenolone	0.2 ± 0.13	1.9 ± 0.12
17α-hydroxypregnenolone	0	0
Progesterone	523 ± 27.4	4476 ± 75
17α-hydroxyprogesterone	0.1 ± 0.01	0.9 ± 0
5α-dihydroprogesterone	8.4 ± 0.4	123 ± 0.6
DHEA	0.4 ± 0	7.4 ± 0.14
Androstenedione	0	0
Testosterone	0.1 ± 0.05	0.6 ± 0.06
5α-dihydrotestosterone	0.2 ± 0.02	0.1 ± 0
**Total:**	**532.4**	**4609.9**

Treatment with 30 µM testosterone:

	**Shoots**	**Roots**
Pregnenolone	0	0.3 ± 0.06
17α-hydroxypregnenolone	0	0
Progesterone	0.2 ± 0.03	2.6 ± 0.1
17α-hydroxyprogesterone	0	0
5α-dihydroprogesterone	0	2.4 ± 0.08
DHEA	0.4 ± 0.03	4.4 ± 0.14
Androstenedione	0	0
Testosterone	50 ± 1.8	103.4 ± 3.3
5α-dihydrotestosterone	0.5 ± 0.06	20.7 ± 0.6
**Total:**	**51.1**	**133.8**
